# Assessing Hospital Surgical Quality

**DOI:** 10.1097/AS9.0000000000000610

**Published:** 2025-09-23

**Authors:** Jeffrey H. Silber, Paul R. Rosenbaum, Joseph G. Reiter, Alexander S. Hill, Lee A. Fleisher, Omar I. Ramadan, Rachel R. Kelz

**Affiliations:** From the * Department of Pediatrics, Perelman School of Medicine, University of Pennsylvania, Philadelphia, PA; † Center for Outcomes Research, Children’s Hospital of Philadelphia, Philadelphia, PA; ‡ The Leonard Davis Institute of Health Economics, The Wharton School of the University of Pennsylvania, Philadelphia, PA; § Department of Health Care Management, The Wharton School of the University of Pennsylvania, Philadelphia, PA; ‖ Department of Statistics and Data Science, The Wharton School of the University of Pennsylvania, Philadelphia, PA; ¶ Department of Anesthesiology and Critical Care, Perelman School of Medicine at the University of Pennsylvania, Philadelphia, PA; # Division of Vascular and Endovascular Surgery, Department of Surgery, Massachusetts General Hospital/Harvard Medical School, Boston, MA; ** Department of Surgery, Perelman School of Medicine at the University of Pennsylvania, Philadelphia, PA.

**Keywords:** hospital grades, hospital quality, hospital report cards, matching, medicare claims, mortality

## Abstract

**Objective:**

Develop a new hospital surgery report card for use in performance improvement.

**Background:**

When evaluating quality, a surgical program is aided by benchmark comparisons with outcomes achieved at other hospitals. To be credible, benchmarking should be based on the same surgical procedures and patient risk, despite there being many types of patients and procedures.

**Methods:**

Using Medicare patients undergoing general, orthopedic, or vascular surgery, each patient in a hospital is closely matched to 10 control patients from typical hospitals and to 10 control patients from well-resourced hospitals throughout the United States. Patients were matched on 200 characteristics, including procedure, comorbidities, socio-demographics, and the presence of multimorbidity. Hospitals were graded based on the differences in outcomes between matched sets of patients. As an illustration, we examine the 20 highest volume hospitals in Pennsylvania and provide detailed report cards on 2 example hospitals.

**Results:**

The hospitals studied differed in quality and grades, with better outcomes than matched controls for Hospital A and significantly worse outcomes than controls for Hospital B, depending on the type of surgery and patient. For the 20 largest hospitals in Pennsylvania, 5 had significantly elevated mortality, and 2 had significantly lower mortality than matched controls.

**Conclusions:**

Surgical programs benefit from knowing how their outcomes compare with those of other hospitals, both their overall outcomes and their outcomes for subsets of patients, such as patients with or without multimorbidity. Detailed reports based on matching can help identify meaningful deficiencies and strengths in programs concerning specific surgeries and patient types.

## INTRODUCTION

Assessing the quality of hospital surgical programs based on outcomes has been attempted for years.^1^ While various organizations have developed methods to do this, often in the form of report card-like outputs,^2–8^ few provide information that is simultaneously transparent, refined, and comprehensive. Some grading systems are proprietary, making it unclear how evaluations are made, while others are detailed but rely on regression models requiring modeling assumptions, which may limit their ability to fairly compare rare procedures.^9^

Several challenges exist in formulating fair report cards for surgical programs. A report card should compare a hospital’s outcomes to those of patients with similar comorbid conditions undergoing similar procedures. It is a common mark of excellence that a surgical program has extensive experience and success with comparatively rare and challenging procedures, but it is difficult to evaluate outcomes for a single rare procedure precisely because the procedure may not be commonly performed at a single hospital. Additionally, surgical departments may perform strikingly different mixtures of procedures, some more challenging than others, and may serve patient populations with very different mixtures of comorbidities.

This article applies matching for many risk factors and procedures to transparently evaluate surgical quality that better compares outcomes for procedures, rare or common. It draws on a growing literature using multivariate matching to evaluate hospitals.^10–14^ Furthermore, we are able to assess quality for surgical patients with and without multimorbidity^15–19^ and better understand how a hospital’s resources influence its outcomes. We apply the method to 90 general, orthopedic, and vascular surgical procedures, creating samples for every hospital in Pennsylvania matched for the 87 distinct procedures plus 110 patient risk factors (including sociodemographic variables), and reporting in detail about the 20 largest hospitals in Pennsylvania. Each hospital receives a detailed report card useful in reviewing its own performance, and we present 2 such report cards, one for a large urban, very major teaching hospital, called Hospital A, and the other for a regional hospital in a diverse, geographically large Hospital Referral Region, called “Hospital B.” The 2 report cards focus on 30-day mortality, readmissions, and revisits, but can easily be expanded to include other outcomes.

## METHODS

### Overview

Using advances in matching and computing methods applied to the vast Centers for Medicare and Medicaid Services (CMS) Virtual Data Resource Center (VDRC), we find 10 control patients for each patient in the hospital of interest, henceforth the “focal hospital.” The 10 controls are from the same surgical procedure group and share the same multimorbidity status;^15,16^ moreover, the match produces a 10-fold larger control group whose patients have a similar mix of comorbid conditions, binary or trinary components of multimorbidity, and similar demographic characteristics, such as age, sex, and sociodemographic variables such as neighborhood poverty. We do this twice, once with 10 patients from typical hospitals and once with 10 other patients from well-resourced hospitals.

A hospital is responsible for its adjusted outcomes, no matter how it produced them, but it is useful to also ask whether a hospital’s outcomes are predictable from its resources or whether its outcomes are inexplicable in terms of its resources. For this purpose only, we also construct an “Analogous” match, that is, one with patients treated at hospitals with similar resources and having similar patients undergoing similar procedures, to the focal hospital.^14^

Matching also permits an evaluation to refocus on specific subpopulations of patients, for instance, patients with or without multimorbidity. For example, one might discover that a focal hospital exhibits superior outcomes for patients with multimorbidity, a fact that might be obscured by failing to distinguish multimorbid patients.^15,16,20^

### Patients and Data

We utilized the CMS VRDC to examine fee-for-service Medicare beneficiaries aged 66 and above admitted to acute care hospitals in the United States from 2017 through 2019. We selected patients who were admitted for surgical procedures in general, orthopedic, and vascular surgery. A complete list of the included procedure codes is provided in Supplemental Tables 1–3, https://links.lww.com/AOSO/A534.

The study was approved by the Children’s Hospital of Philadelphia Institutional Review Board.

### Constructing Control Groups

We consider our hospital of interest our “focal” hospital. This study used 3 matched groups associated with each focal hospital of interest: “Well-Resourced” controls, “Typical” controls, and “Analogous” matched patients. Each patient in each focal hospital was matched to 10 patients treated at well-resourced hospitals, 10 other patients treated at typical hospitals, and 10 other control patients treated at Analogous hospitals. Each of the 3 matches always used the same 200 patient variables; see below and Supplemental Table 4, https://links.lww.com/AOSO/A534 for details.

The first 10 matched controls consisted of patients who had been admitted to well-resourced hospitals, defined as hospitals with all 3 of the following characteristics as developed in previously published work:^16,21^ (1) major or very major teaching hospitals (resident-to-bed ratios ≥0.25); (2) nursing skill-mix [registered nurse/(registered nurse+licensed practical nurse)] above the median; and (3) surgical specialty-specific patient volume above the median.

The second 10 control patients were drawn from a pool of all postoperative general, orthopedic, or vascular surgery patients admitted to any acute care hospital throughout the United States. We call this the “typical” control group because these patients could be from any hospital that treated Medicare patients with any of the surgical procedures included in this study. A control patient who received care at a well-resourced hospital might be in either the well-resourced or typical control group; however, no individual patient appears in both matched-control groups.

The third matched group is the “Analogous” match. Unlike the well-resourced and typical matches, the Analogous match is never used to grade the focal hospital; rather, it helps contextualize why the focal hospital performed as it did. The Analogous patients are similar to patients in the focal hospital, matched on the same 200 patient variables, but the Analogous match also adds in the following hospital characteristics: resident-to-bed ratio, hospital bed count, general surgery volume, orthopedic surgery volume, vascular surgery volume, high-technology status, nurse-to-bed ratio, and nursing skill-mix. Like the other matched-control groups, the Analogous match group contains 10 times more patients than the focal hospital. This is particularly helpful if the focal hospital is small,^22^ as its mortality rates are unstable, making it difficult to assess how similar patients would fare at similar hospitals. The Analogous match answers this question with a 10 times larger sample size. As aforementioned, the focal hospital is never graded in comparison to the Analogous match, because poor performance by the focal hospital is still poor performance that one may want to avoid, even if its poor performance may be attributable to limited resources.

### Constructing the Three Multivariate Matches

We formed 200 patient covariates consistent with Ramadan et al^16,21^ definitions using ICD-10 codes from grouped CMS Chronic Conditions Warehouse variables,^23^ and updated definitions from Silber et al.^24,25^ We matched exactly for 3 surgery types plus 87 clinically-relevant surgical principal procedure groups among general surgery (N = 32 groups), orthopedic surgery (N = 33 groups), and vascular surgery (N = 22 groups) (see Supplemental Table 4, https://links.lww.com/AOSO/A534 for details) for a total of 90 categories. For example, a patient admitted to a focal hospital for a pancreatectomy was only matched to a control patient who also had a pancreatectomy.

Although matching on the 3 surgical types was always exact, matching was almost always exact for the 87 surgical procedures, meaning that in searching the nation for 20 controls, with or without multimorbidity, the first priority was to match for the 87 surgical procedures, and failure to achieve an exact match was exceedingly rare.^26^ For instance, in a total of 41,260 matches (=2063 surgical patients at Hospital A × 10 controls per focal patient × 2 control matches [well-resourced and typical] with 87 procedure groups), there were fewer than 11 instances where a focal patient was matched to a control who was not a member of the same procedure group, that is, ≤11/41,260 = 0.00027, that is, ≤0.027% for Hospital A. We use an N>11 cutoff because CMS forbids discussion of nonzero cell sizes less than 11. For the ≤11 patients in Hospital A who could not be exactly matched within their procedure group, procedures within the same surgical type with similar risk were substituted when making comparisons.

Hospital B had 2251 patients, so there were 45,020 matches (=2,251 patients × 10 controls per patient × 2 control matches), with 0 mismatches.

There were over 2.613 million potential surgical control patients in the VDRC database who might be paired with each patient in a focal hospital. Thus, if we were to match a focal hospital with 2500 surgical patients over 3 years, we would have an enormous matching ratio of 2,600,000/2500 = 1040 potential controls for each focal patient, of whom we must pick 20 (10 controls per patient × 2 control matches). Having selected a balanced control group that is 10 times larger than the focal group, these selected controls are rearranged into 1-to-10 matched sets by minimizing a robust covariate distance.^27^

Match quality was appraised using the difference in covariate means as a fraction of the standard deviation of the covariate. We aimed for a standardized difference below 0.1, though a more common, more lenient target is below 0.2.^28,29^

### Matching Methods

Because we were matching each focal hospital to all Medicare patients undergoing similar inpatient procedures in the United States, the matching algorithm needed to address the enormous asymmetry in the size of these 2 groups. The algorithm required an exact match for procedure group (32 for general surgery, 33 for orthopedic surgery, 22 for vascular surgery) and whether the patient had multimorbidity. The remainder of the 200 patient variables were balanced by a sequential exchange algorithm as described in the Supplemental Table 2, https://links.lww.com/AOSO/A534. For the well-resourced and typical control groups, the algorithm examined a random surgical control patient in Medicare and, if that patient improved a measure of the balance of 200 variables, that control patient was swapped for a control patient currently in the match. The algorithm stopped when all 200 absolute standardized differences were less than 0.01 or the end of all eligible CMS patients was reached. See Supplemental Table 2, https://links.lww.com/AOSO/A534, for details.

### Defining Multimorbidity

In our previous work,^15,16^ we defined multimorbidity for older surgical patients as the presence of at least one cluster of comorbidities—termed qualifying comorbidity sets—confidently associated with at least double the odds of 30-day mortality compared with the typical patient undergoing the same procedure in the same age group. We also incorporate functional status indicators, allowing us to identify particularly high-risk patients.

### Defining Outcomes

Mortality was defined as all-cause, all-location, death within 30 days from admission. Readmission was defined as readmission within 30 days of discharge. Revisit was defined as readmission or a visit to any emergency department within 30 days of discharge. Note that deaths were considered an event for readmissions or revisits to not give credit to a hospital if a patient died before they could be readmitted or visit an emergency department.

### Assigning Grades

For each hospital, outcome rates are compared to its matched controls, yielding a comparison score. The worst 10% received a grade of D, the next 30% received a C (technically, here, a C is still well below the 50th percentile), the next 30% a B, the next 20% an A, and the top 10% an A+ (see Supplemental Table 3, https://links.lww.com/AOSO/A534). Every report card included a focal hospital grade for the well-resourced match and one for the typical match; moreover, each outcome (mortality, readmissions, and revisits) received its own grade. Of course, any division of numerical rates into letter grades is somewhat arbitrary, and we chose a fairly lenient grading curve.

### Benchmarking Methodology

In this article, we will concentrate on 30-day mortality, readmissions, and revisits, but any available outcomes, costs, or payments could have been compared. We will report the difference in the outcome rates between the focal patients and their matched-control patients.

### Statistical Analysis

#### Statistical Methods

We used methods for paired binary data^30^ to construct 95%- and 2/3-confidence intervals for rates and grades. A 2/3-confidence interval is similar to an estimate plus/minus its standard error, and it covers the population rate twice as often as it misses, so it reflects the “preponderance of the evidence”, whereas a 95% interval is stronger evidence.^31^ All *P* values are two-sided. When considering 20 hospitals at once, we additionally report adjustments for multiple testing using the Bonferroni-Holm method.^32^ Although adjustments for multiple testing are relevant in a scientific article that compares all the large hospitals in Pennsylvania, neither patients nor hospital administrators are likely to compare that many hospitals, so these adjustments are too conservative for the typical use of a report card. These issues are discussed in further detail in Supplemental Material 6, https://links.lww.com/AOSO/A534.

## RESULTS

We present report cards on 2 example hospitals in Pennsylvania, labeled Hospital A and Hospital B. Similar results are summarized for the 20 largest hospitals in Pennsylvania.

### Match Quality

Table 1 compares selected risk factors between Focal Hospital A and its two 10-to-1 matched-control groups, and Hospital B and its two matched-control groups, showing that the matching was successful at producing similar groups in terms of these risk factors. An associated table for Hospitals A and B showing all 200 patient variables is in the Supplemental Table 4, https://links.lww.com/AOSO/A534. Table 1 also lists the distribution of select surgical procedures, which was notably very different between Hospitals A and B. Hospital A performed mostly general surgery (53.5% of patients), and only 19.8% of patients underwent orthopedic surgery, whereas only 31.2% of Hospital B’s patients underwent general surgery, while 48.3% had orthopedic surgery. Thus, a report card for Hospital A would need to be compared with very different control patients than those needed for Hospital B, because the patients were so different.

**TABLE 1.** Balance for Focal Hospitals A and B by Matched Controls Treated at Well-Resourced and Typical Hospitals

Variable Labels (49 Out of a Total of 200 Variables on Complete Balance Table, see Supplemental Table 1, https://links.lww.com/AOSO/A534)	Focal Hospital (A)	Matched Controls for Focal Hospital A		Focal Hospital (B)	Matched Controls for Focal Hospital B	
		Well-Resourced Controls	Typical Controls		Well-Resourced Controls	Typical Controls
N patients	2063	20,630	20,630	2251	22,510	22,510
3 Divisions inside each hospital surgery department: divisions of general surgery, orthopedic surgery, and vascular surgery						
General surgery (%)	53.5	53.5	53.5	31.2	31.2	31.2
Orthopedics (%)	19.8	19.8	19.8	48.3	48.3	48.3
Vascular surgery (%)	26.7	26.7	26.7	20.5	20.5	20.5
8 general surgery categories out of 32 general surgery categories provided in supplemental digital content						
Colectomy (%)	12.7	12.7	12.7	7.5	7.5	7.5
Pancreatectomy (%)	6.7	6.7	6.7	1.6	1.6	1.6
Cholecystectomy (%)	4.7	4.7	4.7	6.0	6.0	6.0
Esophagectomy (%)	2.3	2.3	2.3	#	#	#
Gastrectomy (%)	2.3	2.3	2.3	#	#	#
Gastric bypass (%)	1.1	1.1	1.1	#	#	#
Bariatric (%)	0.9	0.9	0.9	0.6	0.6	0.6
Splenectomy (%)	0.8	0.8	0.8	#	#	#
7 orthopedic surgery categories out of 33 orthopedic surgery categories provided in supplemental digital content						
Thoracic/lumbar/sacral fusion (%)	8.8	8.8	8.8	6.3	6.3	6.3
Spinal decompression (%)	3.5	3.5	3.5	1.6	1.6	1.6
Cervical fusion (%)	3.0	3.0	3.0	2.3	2.3	2.3
Spinal cord lesion/excision (%)	1.0	1.0	1.0	#	#	#
Femur repair (%)	0.6	0.6	0.6	8.8	8.8	8.8
Total hip replacement (%)	#	#	#	7.2	7.2	7.2
Total knee replacement (%)	#	#	#	6.9	6.9	6.9
8 vascular surgery categories out of 22 vascular surgery categories provided in supplemental digital content						
Carotid endarterectomy (%)	5.0	5.0	5.0	3.9	3.9	3.9
Endovascular aneurysm repair (%)	4.7	4.7	4.7	2.8	2.8	2.8
Open infrainguinal revascularization (%)	2.6	2.6	2.6	2.0	2.0	2.0
Infrainguinal peripheral vasc interv (%)	2.0	2.0	2.0	1.3	1.3	1.3
Thoracic endovascular aortic repair (%)	1.7	1.7	1.7	#	#	#
Open Abd aortic aneurysm repair (%)	1.6	1.6	1.6	0.6	0.6	0.6
Carotid artery stent (%)	1.5	1.5	1.5	1.6	1.6	1.6
Endo abd artery revascularization (%)	1.1	1.1	1.1	0.7	0.7	0.7
10 comorbidities out of 55 comorbidities + 30 common multimorbidity types =55 + 30=85. (see supplemental digital content)						
Chronic pulmonary diseases (%)	28.2	28.4	28.4	28.5	28.3	28.4
Cardiac arrhythmias (%)	27.8	27.4	27.4	28.6	28.2	28.2
Diabetes with complications (%)	26.1	26.5	26.5	28.1	28.3	28.2
Heart failure (%)	24.7	24.3	24.3	27.1	27.0	26.7
Protein-calorie malnutrition (%)	21.6	21.3	21.3	13.8	13.5	13.5
Diabetes without complication (%)	10.8	11.1	10.9	8.0	8.2	8.1
Morbid obesity (%)	9.0	9.2	9.2	15.8	15.5	15.5
CKD Stage 4–5 or dialysis (%)	8.0	7.8	7.8	8.5	8.3	8.3
Alzheimer’s disease and related dementias (%)	6.2	6.0	5.9	16.5	16.2	16.1
Home oxygen use (%)	4.2	4.0	4.0	6.9	6.7	6.7
3 of 4 summaries using 55 comorbidities and 226 multimorbidity types. (see supplemental digital content)						
#Comorbidities of 55 types (mean)	8.0	8.0	8.0	7.8	7.8	7.8
#Multimorbidities of 226 types (mean)	2.9	2.8	2.8	3.3	3.3	3.3
Any multimorbidity of 226 types (%)	58.1	58.1	58.1	56.2	56.2	56.2
8 socioeconomic and demographic variables out of 21 provided in supplemental digital content						
Age at date of surgery (years, mean)	74.2	74.2	74.2	75.8	75.7	75.7
Age 85+ (%)	7.8	7.4	7.5	13.6	13.3	13.3
Male (%)	50.5	50.4	50.1	44.0	43.5	43.5
White non-Hispanic (%)	84.0	84.1	84.1	97.9	97.6	97.6
Dual-eligible (%)	7.8	8.1	8.0	19.1	18.9	19.0
High poverty status* (%)	7.1	7.0	7.0	1.9	2.1	2.2
Low education status†(%)	5.2	5.5	5.5	3.6	3.9	3.9
Emergent type of admission (%)	29.4	29.0	29.0	32.6	32.2	32.2

All 200 Standardized Differences in covariate means were less than 0.01 in absolute value for each match, that is, extremely small.

* High poverty status.

† Low education status (Less than 80% with high school diploma).

# Not reportable due to CMS cell size requirements.

We achieved an excellent balance between focal hospital patients and their matched controls for both Hospital A and Hospital B. For both Hospitals A and B, the largest absolute standardized difference between the focal patients and well-resourced and typical controls was <0.01, far below our target of 0.10. For Hospital A, the mean patient age was 74.2 years, versus 74.2 years in the typical and well-resourced matches. For Hospital B, the mean age was 75.8 years, and both matched-control groups had mean ages of 75.7. For Hospital A, 24.7% of its patients had a history of heart failure, versus 24.3% for its matched controls. For Hospital B, 27.1% of patients had a history of heart failure, versus 27.0% and 26.7% in its matched controls.

Results of matching for hospital characteristics are in Supplemental Table 5a and 5b with definitions https://links.lww.com/AOSO/A534.

### Hospital A Report Card (Mortality)

**FIGURE 1.**
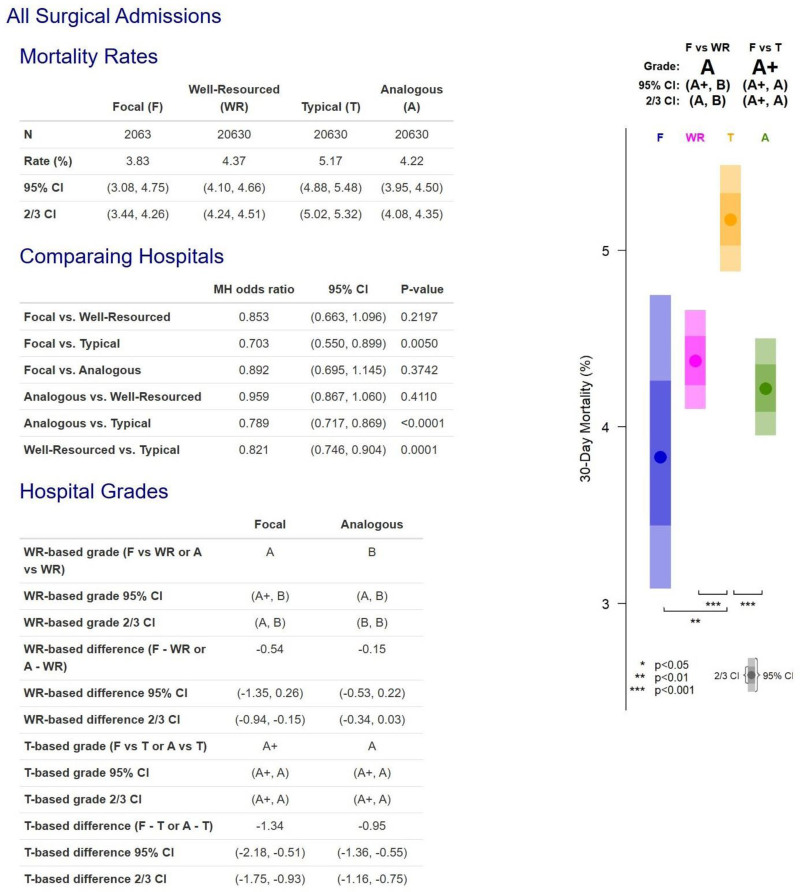
Hospital A report card on 30-day mortality for combined general, orthopedic, and vascular surgery.

Hospital A’s report card on 30-day mortality is presented in Figure 1. Hospital A performed 2063 procedures (combined general, orthopedic, and vascular surgery) over a 3-year period, with a death rate of 3.83% versus 4.37% for 20,630 matched patients from well-resourced hospitals (*P* = 0.22) and 5.17% (*P* = 0.0050) for 20,630 matched patients from typical hospitals throughout the United States. Thus, Hospital A’s death rates were below the 2 control groups, producing grades of A versus well-resourced and A+ versus typical hospitals. Furthermore, 20,630 patients from the Analogous match—patients who were both similar in terms of procedures, demographics, and risk factors and also treated at hospitals with similar characteristics to Hospital A—had a death rate of 4.22%, not significantly different than Hospital A’s death rate (*P* = 0.37). Hospital A appears to be performing excellently and in line with its excellent resources.

### Hospital B Report Card (Mortality)

**FIGURE 2.**
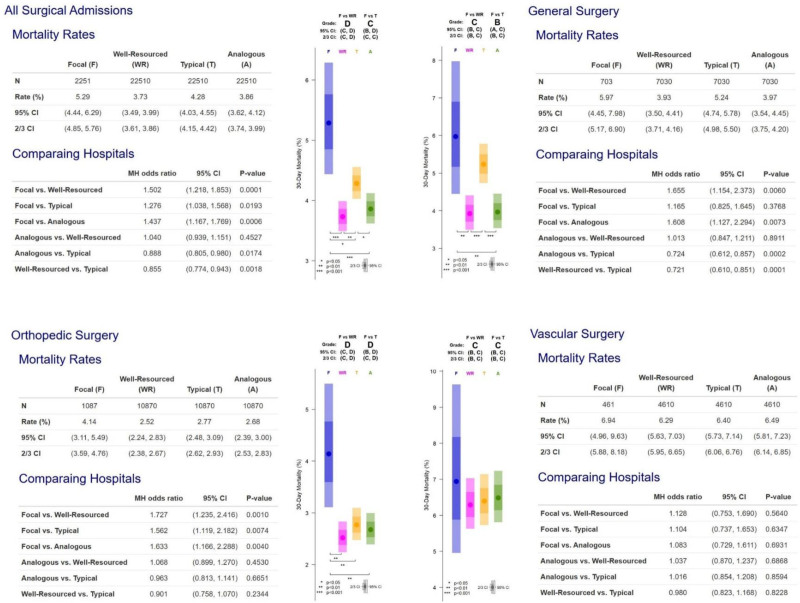
Hospital B report card on 30-day mortality over all divisions (general, orthopedics, and vascular surgery combined), and each division separately.

Hospital B’s report card on 30-day mortality is presented in Figure 2. Its grades were poor, with Hospital B receiving a D versus well-resourced hospitals and a C versus typical hospitals. The death rate for all of Hospital B’s surgical patients was 5.29%, versus 3.73% in patients treated at well-resourced hospitals (*P* = 0.0001) and 4.28% in patients at typical hospitals (*P* = 0.019). Furthermore, patients from the Analogous match had a lower death rate (3.86%, *P* = 0.0006), suggesting that Hospital B is doing worse than would be expected for its same patient mix and hospital resources.

The report card in Figure 2 also provides death rates and comparisons by surgical type. Grades were different across types of surgery. Hospital B received particularly poor grades for orthopedic surgery.

**FIGURE 3.**
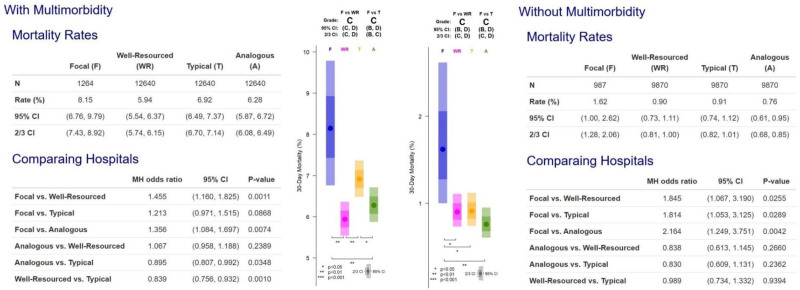
Hospital B report card on 30-day mortality for all surgery combined, and broken down by patients with and without multimorbidity.

Figure 3 provides Hospital B’s grades for patients with and without multimorbidity. Note that the focal hospitals are always compared with patients with similar characteristics in the control groups who underwent the same procedures. While patients with multimorbidity—relative to those without multimorbidity—have a much higher risk of death at any given hospital, the report in Figure 3 demonstrates that Hospital B has comparatively high mortality rates for both patients with and without multimorbidity.

### Readmissions and Revisits Report Cards

Supplemental Figure 1 and Figure 2, https://links.lww.com/AOSO/A534 provide readmissions and revisits for Hospitals A and B. Hospital A received grades of A and A for readmissions and revisits, and Hospital B received grades of C and C.

### Results for the 20 Largest Hospitals in Pennsylvania

Table 2 provides summary results for the 20 largest hospitals in Pennsylvania based on the surgical volume of our study procedures. As seen in Table 2, 5 of the 20 hospitals have substantially and significantly higher surgical mortality than matched control patients from well-resourced hospitals; 2 of the 5 were worse than typical controls. Furthermore, 2 other hospitals performed better than typical controls. Although 20 hospitals were compared, 3 hospitals have significantly higher mortality than well-resourced controls after the conservative Bonferroni-Holm adjustment for multiple testing.

**TABLE 2.** Grades, adjusted odds ratios, and *P*-values for mortality at the 20 largest by volume Pennsylvania hospitals

			Mortality vs. 10 × N Control Patients From Well-Resourced Hospitals in US					Mortality vs. 10 × N Control Patients From Typical Hospitals in US				
Focal Provider	N Focal	Mortality Rate Focal	Mortality Rate WR	Odds Ratio	*P* value	Grade	95% CI	Mortality Rate Typical	Odds Ratio	*P* value	Grade	95% CI
1	4849	2.52%	2.59%	0.966	0.7316	B	(A, B)	3.10%	0.788	0.0176*	A	(A+, B)
2	4728	2.41%	2.03%	1.219	0.0632	C	(B, C)	2.38%	1.016	0.8783	B	(A, C)
3	3994	2.30%	2.51%	0.909	0.4060	B	(A, B)	2.74%	0.825	0.0910	A	(A+, B)
4	3816	2.39%	1.77%	1.402	0.0046*	C	(B, C)	2.24%	1.074	0.5406	B	(A, C)
5	3299	3.85%	3.55%	1.099	0.3518	B	(A, C)	4.20%	0.902	0.3124	A	(A, B)
6	2760	3.44%	2.90%	1.216	0.0930	C	(B, C)	3.16%	1.109	0.3897	B	(A, C)
7	2741	1.50%	1.16%	1.310	0.1121	C	(B, C)	1.45%	1.033	0.8484	B	(A, C)
8	2710	3.47%	3.20%	1.095	0.4334	B	(A, C)	3.76%	0.912	0.4219	B	(A+, B)
9	2628	3.58%	3.30%	1.099	0.4260	B	(A, C)	3.38%	1.069	0.5676	B	(A, C)
10 (Hosp B)	2251	5.29%	3.73%	1.502	0.0001†	D	(C, D)	4.28%	1.276	0.0193*	C	(B, D)
11	2117	2.93%	2.43%	1.232	0.1424	C	(B, C)	2.62%	1.138	0.3737	B	(A, C)
12	2100	4.38%	4.33%	1.014	0.9062	B	(A, C)	5.10%	0.842	0.1369	A	(A+, B)
13 (Hosp A)	2063	3.83%	4.37%	0.853	0.2197	A	(A+, B)	5.17%	0.703	0.0050*	A+	(A+, A)
14	1890	4.07%	3.19%	1.332	0.0297*	C	(B, D)	3.55%	1.174	0.2243	C	(A, C)
15	1869	2.03%	2.28%	0.879	0.4700	A	(A+, C)	2.48%	0.802	0.2118	A	(A+, B)
16	1855	2.16%	1.32%	1.677	0.0025‡	C	(B, D)	1.65%	1.352	0.0932	C	(B, D)
17	1841	2.99%	3.25%	0.908	0.5239	B	(A+, C)	3.73%	0.777	0.0888	A	(A+, B)
18	1758	4.66%	2.91%	1.739	<0.0001†	D	(C, D)	3.32%	1.475	0.0021‡	C	(B, D)
19	1703	1.94%	2.27%	0.839	0.3585	A	(A+, B)	2.50%	0.753	0.1360	A	(A+, B)
20	1650	3.33%	2.63%	1.316	0.0774	C	(B, D)	3.16%	1.062	0.6934	B	(A, C)

N refers to total study procedure volume over 3 years.

Odds ratio refers to Mantel–Haenszel odds ratio.

* Conventional *P* value≤0.05.

† Bonferroni-Holm adjusted *P* value ≤0.01.

‡ Bonferroni-Holm adjusted *P* value ≤0.05.

## DISCUSSION

Detailed, transparent surgical report cards were constructed using multivariate matching using three comparison groups—typical hospitals, well-resourced hospitals, and hospitals with similar resources—with the ability to focus on subsets of patients, such as those with multimorbidity. Using the CMS VDRC, excellent matches were achieved within general, orthopedic, and vascular surgery across 87 procedure groups, controlling for 110 patient risk factors such as multimorbidity status and comorbidities, and sociodemographic variables such as poverty.

Unlike widely used grading schemes based on logistic regression that issue an abstract grade, our report cards are diagnostic. A poor grade may be disappointing, whereas an accurate diagnosis might prompt practical, effective intervention. For instance, a hospital with consistently poor results in orthopedic procedures for patients with multimorbidity might focus its quality improvement efforts, or if that fails, might raise its grades by referring these patients to hospitals with better resources, such as better nursing and a higher volume of similar patients.

Each patient in the focal hospital is matched to 10 controls from each of 3 sources of controls—typical, well-resourced, and Analogous—so almost all of the sampling noise comes from the limited sample size in the focal hospital; therefore, using more controls would negligibly reduce sampling error while making controls less like the focal patient in relevant ways.^33–36^ With 10 controls of each type, we can examine subsets of patients. Here, we selected multimorbid patients for separate examination, and similarly, we separated surgical types. With careful use of modern matching methods, a single match can be reconstructed to examine a wide variety of subsets.^37,38^

We compare a focal hospital’s patients to similar patients at other hospitals. In contrast, the logit models used by CMS and National Surgical Quality Improvement Program judge a hospital using a formula derived from some similar patients and from some dissimilar patients, perhaps severely multimorbid patients who might rarely, if ever, receive surgery at the focal hospital. Chattopadhyay and Zubizarreta,^39^ show that model-based adjustments unaided by matching can attach large yet bizarre weights to these irrelevant patients; see also Rubin.^40^

Though Medicare data is vast, uniform, with all-location mortality, it has limitations. The method could be applied to younger patients using insurance and electronic health records, provided that data are uniformly collected.

Experience and skill with rare and often challenging procedures is one mark of excellence in a surgical program. Hospitals vary in the type of procedures performed. There are 2 problems: our method addresses both. First, for any 1 rare procedure, the volume at most hospitals is too small to provide stable outcome rates. Second, rare procedures are different from each other, so a stable outcome rate cannot reasonably be obtained by merging many rare procedures into one. The matching method addresses both problems by using all of the procedures performed by a focal hospital, rare or common, while comparing the focal hospital’s outcomes to matched controls with the same procedure groups.

Surgical programs, payers, and patients can all benefit from more transparent and more comprehensive report cards that permit attention to refocus on subgroups of patients or procedures and allow for comparison to different relevant control groups. Using multivariate matching, surgical programs can better and more confidently identify areas where quality is strong or where significant improvement is needed.

## ACKNOWLEDGMENTS

J.H.S., P.R.R., J.G.R., L.A.F., and R.R.K. participated in research design. J.H.S., P.R.R., J.G.R., A.S.H., L.A.F., O.I.R., and R.R.K. participated in the writing of the paper, performance of the research, data analysis, reviewed the manuscript, and believe it represents valid work. P.R.R. and J.G.R. contributed new reagents or analytic tools. All authors approve for submission.

## Supplementary Material


