# Supplementary material for: Assessing Hospital Surgical Quality
Source: Ann Surg Open. 2025 Sep 23;6(4):e610. doi: 10.1097/AS9.0000000000000610 (PMC12727418; doi:10.1097/AS9.0000000000000610)
Supplement: Supplementary file 1 [file as9-6-e610-s001.pdf]

Annals of Surgery Open

August 7, 2025

Appendix

Assessing Hospital Surgical Quality

Table of Contents

Supplemental Digital Content 1. Codes for Included General Surgery Procedures 2

Supplemental Digital Content Table 1. List of ICD-10 principal procedure codes for General Surgery, categorized into clinically relevant procedure groups 2

Supplemental Digital Content Table 2. List of ICD-10 principal procedure codes for Orthopedic Surgery, categorized into clinically relevant procedure groups 5

Supplemental Digital Content Table 3. List of ICD-10 principal procedure codes for Vascular Surgery, categorized into clinically relevant procedure groups 13

Supplemental Digital Content 2. Details of the Sequential Exchange Matching Algorithm 24

Supplemental Digital Content 3. Assigning Grades and Cut Points 27

Supplemental Digital Content 4. Balance Tables 28

Supplemental Digital Content Table 4. Balance Table for Focal Hospitals A and B by Matched Control Patients Treated at Well-Resourced and Typical Hospitals 28

Supplemental Digital Content 5. Hospital Balance Tables 36

Supplemental Digital Content Table 5a. Balance Table of Hospital Characteristics for Hospital A 36

Supplemental Digital Content Table 5b. Balance Table of Hospital Characteristics for Hospital B 37

Supplemental Digital Content 6: Discussion of Multiple Testing Issues When Using Report Cards 38

Supplemental Digital Content 7: Results for Readmissions and Revisits For Example Hospitals A and B 39

Supplemental Digital Content Figure 1. Hospital A report cards on Readmission and Revisits for combined general, orthopedics and vascular surgery 39

Supplemental Digital Content Figure 2. Hospital B report cards on Readmission and Revisits for combined general, orthopedics and vascular surgery 40

References 41

Supplemental Digital Content 1. Codes for Included General Surgery Procedures

Supplemental Digital Content Table 1. List of ICD-10 principal procedure codes for General Surgery, categorized into clinically relevant procedure groups
**Adhesiolysis**<br>0DN60ZZ, 0DN64ZZ, 0DN70ZZ, 0DN74ZZ, 0DN80ZZ, 0DN84ZZ, 0DN90ZZ, 0DN94ZZ, 0DNA0ZZ, 0DNA4ZZ, 0DNB0ZZ, 0DNB4ZZ, 0DNC0ZZ, 0DNC4ZZ, 0DNE0ZZ, 0DNE4ZZ, 0DNF0ZZ, 0DNF4ZZ, 0DNG0ZZ, 0DNG4ZZ, 0DNH0ZZ, 0DNH4ZZ, 0DNJ0ZZ, 0DNJ4ZZ, 0DNK0ZZ, 0DNK4ZZ, 0DNL0ZZ, 0DNL4ZZ, 0DNM0ZZ, 0DNM4ZZ, 0DNN0ZZ, 0DNN4ZZ, 0DNS0ZZ, 0DNS4ZZ, 0DNT0ZZ, 0DNT4ZZ, 0DNV0ZZ, 0DNV4ZZ, 0DNW0ZZ, 0DNW4ZZ
**Adrenal**<br>0GB20ZX, 0GB20ZZ, 0GB24ZX, 0GB24ZZ, 0GB30ZX, 0GB30ZZ, 0GB34ZX, 0GB34ZZ, 0GB40ZX, 0GB40ZZ, 0GB44ZX, 0GB44ZZ, 0GC20ZZ, 0GC24ZZ, 0GC30ZZ, 0GC34ZZ, 0GC40ZZ, 0GC44ZZ, 0GT20ZZ, 0GT24ZZ, 0GT30ZZ, 0GT34ZZ, 0GT40ZZ, 0GT44ZZ
**Appendectomy**<br>0DTJ0ZZ, 0DTJ4ZZ
**Bariatric/Gastrectomy[a]**<br>0DB60Z3, 0DB60ZZ, 0DB64Z3, 0DB64ZZ
**Bariatric/Gastric Bypass[a]**<br>0D160ZA, 0D160ZB, 0D164ZA, 0D164ZB, 0D190ZA, 0D190ZB, 0D194ZA, 0D194ZB, 0D1A0ZA
**Bariatric/Stomach-Other[a]**<br>0DP60CZ, 0DP64CZ, 0DV60CZ, 0DV60ZZ, 0DV64CZ, 0DV64ZZ, 0DW60CZ, 0DW64CZ
**Biliary Common Duct**<br>0F190Z3-0F190Z9, 0F190ZB, 0F194Z3-0F194Z9, 0F194ZB, 0FC90ZZ, 0FC94ZZ, 0FJB0ZZ, 0FJB4ZZ
**Biliary Other**<br>0F140Z3-0F140Z9, 0F140ZB, 0F144Z3-0F144Z9, 0F144ZB, 0F150Z3-0F150Z9, 0F150ZB, 0F154Z3-0F154Z9, 0F154ZB, 0F160Z3-0F160Z9, 0F160ZB, 0F164Z3-0F164Z9, 0F164ZB, 0F170Z3-0F170Z9, 0F170ZB, 0F174Z3-0F174Z9, 0F174ZB, 0F180Z3-0F180Z9, 0F180ZB, 0F184Z3-0F184Z9, 0F184ZB
**Cholecystectomy**<br>0FB40ZX, 0FB40ZZ, 0FB44ZX, 0FB44ZZ, 0FT40ZZ, 0FT44ZZ
**Colectomy**<br>0DBE0ZX, 0DBE0ZZ, 0DBE4ZX, 0DBE4ZZ, 0DBE7ZZ, 0DBF0ZX, 0DBF0ZZ, 0DBF4ZX, 0DBF4ZZ, 0DBF7ZZ, 0DBG0ZX, 0DBG0ZZ, 0DBG4ZX, 0DBG4ZZ, 0DBG7ZZ, 0DBH0ZX, 0DBH0ZZ, 0DBH4ZX, 0DBH4ZZ, 0DBH7ZZ, 0DBK0ZZ, 0DBK4ZZ, 0DBK7ZZ, 0DBL0ZX, 0DBL0ZZ, 0DBL4ZX, 0DBL4ZZ, 0DBL7ZZ, 0DBM0ZX, 0DBM0ZZ, 0DBM4ZX, 0DBM4ZZ, 0DBM7ZZ, 0DBN0ZX, 0DBN0ZZ, 0DBN4ZX, 0DBN4ZZ, 0DBN7ZZ, 0DTE0ZZ, 0DTE4ZZ, 0DTE7ZZ, 0DTF0ZZ, 0DTF4ZZ, 0DTF7ZZ, 0DTG0ZZ, 0DTG4ZZ, 0DTG7ZZ, 0DTH0ZZ, 0DTH4ZZ, 0DTH7ZZ, 0DTK0ZZ, 0DTK4ZZ, 0DTK7ZZ, 0DTL0ZZ, 0DTL4ZZ, 0DTL7ZZ, 0DTM0ZZ, 0DTM4ZZ, 0DTM7ZZ, 0DTN0ZZ, 0DTN4ZZ, 0DTN7ZZ
**Diagnostic Lap**<br>0DJU4ZZ, 0DJV4ZZ, 0DJW4ZZ, 0FJ04ZZ, 0FJ44ZZ, 0FJD4ZZ, 0FJG4ZZ, 0WJF4ZZ, 0WJG4ZZ, 0WJJ4ZZ, 0WJP4ZZ, 0WJR4ZZ

**Enterectomy**<br>0DB80ZX, 0DB80ZZ, 0DB84ZX, 0DB84ZZ, 0DB90ZX, 0DB90ZZ, 0DB94ZX, 0DB94ZZ, 0DBA0ZX, 0DBA0ZZ, 0DBA4ZX, 0DBA4ZZ, 0DBB0ZX, 0DBB0ZZ, 0DBB4ZX, 0DBB4ZZ, 0DBC0ZZ, 0DBC4ZZ, 0DT80ZZ, 0DT84ZZ, 0DT90ZZ, 0DT94ZZ, 0DTA0ZZ, 0DTA4ZZ, 0DTB0ZZ, 0DTB4ZZ, 0DTC0ZZ, 0DTC4ZZ
**Esophagectomy**<br>0DB10ZZ, 0DB20ZZ, 0DB30ZZ, 0DB40ZZ, 0DB44ZZ, 0DB50ZX, 0DB50ZZ, 0DB54ZX, 0DB54ZZ, 0DQ50ZZ, 0DQ54ZZ, 0DT10ZZ, 0DT14ZZ, 0DT20ZZ, 0DT24ZZ, 0DT30ZZ, 0DT34ZZ, 0DT40ZZ, 0DT44ZZ, 0DT50ZZ, 0DT54ZZ
**Esophagomyotomy**<br>0D840ZZ, 0D844ZZ
**Gastrectomy**<br>0D130Z9, 0D130ZA, 0D130ZB, 0D134Z9, 0D134ZA, 0D134ZB, 0D160Z9, 0D164Z9, 0DB60ZX, 0DB64ZX, 0DB70ZZ, 0DB74ZZ, 0DT60ZZ, 0DT64ZZ, 0DT70ZZ, 0DT74ZZ
**Hernia Abdomen**<br>0WQF0ZZ, 0WQF4ZZ, 0WUF07Z, 0WUF0JZ, 0WUF0KZ, 0WUF47Z, 0WUF4JZ, 0WUF4KZ
**Hernia Diaphragm**<br>0BQR0ZZ, 0BQR4ZZ, 0BQS0ZZ, 0BQS4ZZ, 0BQT0ZZ, 0BQT4ZZ, 0BUR07Z, 0BUR0JZ, 0BUR0KZ, 0BUR47Z, 0BUR4JZ, 0BUR4KZ, 0BUS07Z, 0BUS0JZ, 0BUS0KZ, 0BUS47Z, 0BUS4JZ, 0BUS4KZ
**Hernia Groin**<br>0YQ50ZZ, 0YQ54ZZ, 0YQ60ZZ, 0YQ64ZZ, 0YQ70ZZ, 0YQ74ZZ, 0YQ80ZZ, 0YQ84ZZ, 0YQA0ZZ, 0YQA4ZZ, 0YQE0ZZ, 0YQE4ZZ, 0YU507Z, 0YU50JZ, 0YU50KZ, 0YU547Z, 0YU54JZ, 0YU54KZ, 0YU607Z, 0YU60JZ, 0YU60KZ, 0YU647Z, 0YU64JZ, 0YU64KZ, 0YU707Z, 0YU70JZ, 0YU70KZ, 0YU747Z, 0YU74JZ, 0YU74KZ, 0YU807Z, 0YU80JZ, 0YU80KZ, 0YU847Z, 0YU84JZ, 0YU84KZ, 0YUA07Z, 0YUA0JZ, 0YUA0KZ, 0YUA47Z, 0YUA4JZ, 0YUA4KZ, 0YUE07Z, 0YUE0JZ, 0YUE0KZ, 0YUE47Z, 0YUE4JZ, 0YUE4KZ
**Large Bowel-Other**<br>0D1H0ZH, 0D1H0ZK, 0D1H0ZL, 0D1H0ZM, 0D1H0ZN, 0D1H0ZP, 0D1H4ZH, 0D1H4ZK, 0D1H4ZL, 0D1H4ZM, 0D1H4ZN, 0D1H4ZP, 0D1K0ZK, 0D1K0ZL, 0D1K0ZM, 0D1K0ZN, 0D1K0ZP, 0D1K4ZK, 0D1K4ZL, 0D1K4ZM, 0D1K4ZN, 0D1K4ZP, 0D1L0ZL, 0D1L0ZM, 0D1L0ZN, 0D1L0ZP, 0D1L4ZL, 0D1L4ZM, 0D1L4ZN, 0D1L4ZP, 0D1M0ZM, 0D1M0ZN, 0D1M0ZP, 0D1M4ZM, 0D1M4ZN, 0D1M4ZP, 0D1N0ZN, 0D1N0ZP, 0D1N4ZN, 0D1N4ZP, 0DME0ZZ, 0DME4ZZ, 0DMF0ZZ, 0DMF4ZZ, 0DMG0ZZ, 0DMG4ZZ, 0DMH0ZZ, 0DMH4ZZ, 0DMK0ZZ, 0DMK4ZZ, 0DML0ZZ, 0DML4ZZ, 0DMM0ZZ, 0DMM4ZZ, 0DMN0ZZ, 0DMN4ZZ, 0DMP4ZZ, 0DWE07Z, 0DWE47Z
**Liver**<br>0F500ZF, 0F500ZZ, 0F504ZF, 0F504ZZ, 0F510ZF, 0F510ZZ, 0F514ZF, 0F514ZZ, 0F520ZF, 0F520ZZ, 0F524ZF, 0F524ZZ, 0FB00ZX, 0FB00ZZ, 0FB04ZX, 0FB04ZZ, 0FB10ZX, 0FB10ZZ, 0FB14ZX, 0FB14ZZ, 0FB20ZX, 0FB20ZZ, 0FB24ZX, 0FB24ZZ, 0FT10ZZ, 0FT14ZZ, 0FT20ZZ, 0FT24ZZ
**Lymphadenectomy**<br>07T50ZZ, 07T54ZZ, 07T60ZZ, 07T64ZZ
**Mastectomy**<br>0HBT0ZZ, 0HBU0ZZ, 0HBV0ZZ, 0HBY0ZZ, 0HTT0ZZ, 0HTU0ZZ, 0HTV0ZZ
**Ostomy Creation/Reversal**<br>0D190Z4, 0D194Z4, 0D1A0Z4, 0D1A4Z4, 0D1B0Z4, 0D1B4Z4, 0DQ84ZZ, 0DQ90ZZ, 0DQ94ZZ, 0DQA0ZZ, 0DQA4ZZ, 0DQB0ZZ, 0DQB4ZZ, 0DQE0ZZ, 0DQE4ZZ, 0DQE7ZZ, 0DQF0ZZ, 0DQF4ZZ, 0DQF7ZZ, 0DQG0ZZ, 0DQG4ZZ, 0DQG7ZZ, 0DQH0ZZ, 0DQH4ZZ, 0DQH7ZZ, 0DQK0ZZ, 0DQK4ZZ, 0DQK7ZZ, 0DQL0ZZ, 0DQL4ZZ, 0DQL7ZZ, 0DQM0ZZ, 0DQM4ZZ, 0DQM7ZZ, 0DQN0ZZ, 0DQN4ZZ, 0DQN7ZZ, 0WBFXZ2, 0WQFXZ2, 0D1H0Z4, 0D1H4Z4, 0D1K0Z4, 0D1K4Z4, 0D1L0Z4, 0D1L4Z4, 0D1M0Z4, 0D1M4Z4, 0D1N0Z4, 0D1N4Z4, 0DQ80ZZ

**Pancreas-Other**<br>0F1D0Z3, 0F1D0ZB, 0F1D0ZC, 0F1D4Z3, 0F1D4ZB, 0F1D4ZC, 0F1F0Z3, 0F1F0ZB, 0F1F0ZC, 0F1F4Z3, 0F1F4ZB, 0F1F4ZC, 0F1G0Z3, 0F1G0ZB, 0F1G0ZC, 0F1G4Z3, 0F1G4ZB, 0F1G4ZC, 0F5D0ZZ, 0F5F0ZZ, 0F5F4ZZ, 0F5G0ZZ, 0F5G4ZZ, 0FBD0ZZ, 0FBD4ZZ, 0FBF0ZZ, 0FBF4ZZ, 0FTD0ZZ, 0FTD4ZZ, 0FTF0ZZ, 0FTF4ZZ
**Pancreatectomy**<br>0FBG0ZX, 0FBG0ZZ, 0FBG4ZX, 0FBG4ZZ, 0FTG0ZZ, 0FTG4ZZ
**Proctectomy**<br>0D1A4ZQ, 0D1B0ZQ, 0D1B4ZQ, 0D1A0ZQ, 0DTP0ZZ, 0DTP4ZZ, 0DTP7ZZ, 0DTQ7ZZ
**Proctopexy**<br>0DSP0ZZ, 0DSP4ZZ, 0DSP7ZZ, 0DUP0JZ, 0DUP4JZ
**Small Bowel-Other**<br>0D190Z9, 0D190ZL, 0D194Z9, 0D194ZL, 0D1A0ZB, 0D1A0ZH, 0D1A0ZK, 0D1A0ZL, 0D1A0ZM, 0D1A0ZN, 0D1A0ZP, 0D1A4ZB, 0D1A4ZH, 0D1A4ZK, 0D1A4ZL, 0D1A4ZM, 0D1A4ZN, 0D1B0ZB, 0D1B0ZH, 0D1B0ZK, 0D1B0ZL, 0D1B0ZM, 0D1B0ZN, 0D1B0ZP, 0D1B4ZB, 0D1B4ZH, 0D1B4ZK, 0D1B4ZL, 0D1B4ZM, 0D1B4ZN, 0D1B4ZP, 0DM80ZZ, 0DM84ZZ, 0DM90ZZ, 0DM94ZZ, 0DMA0ZZ, 0DMA4ZZ, 0DMB0ZZ, 0DMB4ZZ, 0DQC0ZZ, 0DQC4ZZ, 0DV80CZ, 0DV80ZZ, 0DV84CZ, 0DV84ZZ, 0DV90CZ, 0DV90ZZ, 0DV94CZ, 0DV94ZZ, 0DVA0CZ, 0DVA0ZZ, 0DVA4CZ, 0DVA4ZZ, 0DVB0CZ, 0DVB0ZZ, 0DVB4CZ, 0DVB4ZZ, 0DVC0CZ, 0DVC0ZZ, 0DVC4CZ, 0DVC4ZZ, 0DW807Z, 0DW847Z
**Splenectomy**<br>07BP0ZX, 07BP0ZZ, 07BP4ZX, 07BP4ZZ, 07TP0ZZ, 07TP4ZZ
**Stomach Anti-Reflux**<br>0DV40CZ, 0DV40ZZ, 0DV44CZ, 0DV44ZZ
**Stomach-Other**<br>0D1607L, 0D160ZL, 0D1647L, 0D164ZL, 0D870ZZ, 0D874ZZ, 0D970ZZ, 0D974ZZ, 0DM60ZZ, 0DM64ZZ, 0DV70CZ, 0DV70ZZ, 0DV74CZ, 0DV74ZZ
**Thyroid/Parathyroid**<br>0GBG0ZX, 0GBG0ZZ, 0GBG4ZX, 0GBG4ZZ, 0GBH0ZX, 0GBH0ZZ, 0GBH4ZX, 0GBH4ZZ, 0GBJ0ZX, 0GBJ0ZZ, 0GBJ4ZX, 0GBJ4ZZ, 0GBL0ZX, 0GBL0ZZ, 0GBL4ZX, 0GBL4ZZ, 0GBM0ZX, 0GBM0ZZ, 0GBM4ZX, 0GBM4ZZ, 0GBN0ZX, 0GBN0ZZ, 0GBN4ZX, 0GBN4ZZ, 0GBP0ZX, 0GBP0ZZ, 0GBP4ZX, 0GBP4ZZ, 0GBQ0ZX, 0GBQ0ZZ, 0GBQ4ZX, 0GBQ4ZZ, 0GBR0ZX, 0GBR0ZZ, 0GBR4ZX, 0GBR4ZZ, 0GCG0ZZ, 0GCG4ZZ, 0GCH0ZZ, 0GCH4ZZ, 0GCK0ZZ, 0GCK4ZZ, 0GTG0ZZ, 0GTG4ZZ, 0GTH0ZZ, 0GTH4ZZ, 0GTJ0ZZ, 0GTJ4ZZ, 0GTJ4ZZ, 0GTK0ZZ, 0GTK4ZZ, 0GTL0ZZ, 0GTL4ZZ, 0GTM0ZZ, 0GTM4ZZ, 0GTN0ZZ, 0GTN4ZZ, 0GTP0ZZ, 0GTP4ZZ, 0GTQ0ZZ, 0GTQ4ZZ, 0GTR0ZZ, 0GTR4ZZ
**Ulcer**<br>0DQ60ZZ, 0DQ64ZZ, 0DQ70ZZ, 0DQ74ZZ, 0W3P0ZZ, 0W3P4ZZ

To distinguish bariatric from non-bariatric gastric procedures, we checked whether patients' inpatient bills included a Diagnosis Related Group field for bariatric surgery (619, 620, or 621) or an ICD-10 principal diagnosis code related to obesity. If either was present, the procedure was classified as bariatric; if neither was present, it was considered non-bariatric.

**Supplemental Digital Content Table 2. List of ICD-10 principal procedure codes for Orthopedic Surgery, categorized into clinically relevant procedure groups**
**Ankle procedures**<br>0LMS0ZZ, 0LMS4ZZ, 0LMT0ZZ, 0LMT4ZZ, 0LQS0ZZ, 0LQS3ZZ, 0LQS4ZZ, 0LQT0ZZ, 0LQT3ZZ, 0LQT4ZZ, 0MBQ0ZZ, 0MBQ3ZZ, 0MBQ4ZZ, 0MBR0ZZ, 0MBR3ZZ, 0MBR4ZZ, 0MDQ0ZZ, 0MDQ3ZZ, 0MDQ4ZZ, 0MDR0ZZ, 0MDR3ZZ, 0MDR4ZZ, 0MTQ0ZZ, 0MTQ4ZZ, 0MTR0ZZ, 0MTR4ZZ, 0SBF0ZZ, 0SBF3ZZ, 0SBF4ZZ, 0SBG0ZZ, 0SBG3ZZ, 0SBG4ZZ, 0SGF04Z, 0SGF05Z, 0SGF07Z, 0SGF0JZ, 0SGF0KZ, 0SGF0ZZ, 0SGF34Z, 0SGF35Z, 0SGF37Z, 0SGF3JZ, 0SGF3KZ, 0SGF3ZZ, 0SGF44Z, 0SGF45Z, 0SGF47Z, 0SGF4JZ, 0SGF4KZ, 0SGF4ZZ, 0SGG04Z, 0SGG05Z, 0SGG07Z, 0SGG0JZ, 0SGG0KZ, 0SGG0ZZ, 0SGG34Z, 0SGG35Z, 0SGG37Z, 0SGG3JZ, 0SGG3KZ, 0SGG3ZZ, 0SGG44Z, 0SGG45Z, 0SGG47Z, 0SGG4JZ, 0SGG4KZ, 0SGG4ZZ, 0SPF08Z, 0SPF0JZ, 0SPF38Z, 0SPF3JZ, 0SPF48Z, 0SPF4JZ, 0SPG08Z, 0SPG0JZ, 0SPG38Z, 0SPG3JZ, 0SPG48Z, 0SPG4JZ, 0SRF07Z, 0SRF0J9, 0SRF0JA, 0SRF0JZ, 0SRF0KZ, 0SRG07Z, 0SRG0J9, 0SRG0JA, 0SRG0JZ, 0SRG0KZ, 0STF0ZZ, 0STG0ZZ, 0SUF07Z, 0SUF0JZ, 0SUF0KZ, 0SUF37Z, 0SUF3JZ, 0SUF3KZ, 0SUF47Z, 0SUF4JZ, 0SUF4KZ, 0SUG07Z, 0SUG0JZ, 0SUG0KZ, 0SUG37Z, 0SUG3JZ, 0SUG3KZ, 0SUG47Z, 0SUG4JZ, 0SUG4KZ
**Cervical fusion**<br>0RG0070-0RG0071, 0RG007J, 0RG00A0-0RG00A1, 0RG00AJ, 0RG00J0-0RG00J1, 0RG00JJ, 0RG00K0-0RG00K1, 0RG00KJ, 0RG00Z0-0RG00Z1, 0RG00ZJ, 0RG0370-0RG0371, 0RG037J, 0RG03A0-0RG03A1, 0RG03AJ, 0RG03J0-0RG03J1, 0RG03JJ, 0RG03K0-0RG03K1, 0RG03KJ, 0RG03Z0-0RG03Z1, 0RG03ZJ, 0RG0470-0RG0471, 0RG047J, 0RG04A0-0RG04A1, 0RG04AJ, 0RG04J0-0RG04J1, 0RG04JJ, 0RG04K0-0RG04K1, 0RG04KJ, 0RG04Z0-0RG04Z1, 0RG04ZJ, 0RG1070-0RG1071, 0RG107J, 0RG10A0-0RG10A1, 0RG10AJ, 0RG10J0-0RG10J1, 0RG10JJ, 0RG10K0-0RG10K1, 0RG10KJ, 0RG10Z0-0RG10Z1, 0RG10ZJ, 0RG1370-0RG1371, 0RG137J, 0RG13A0-0RG13A1, 0RG13AJ, 0RG13J0-0RG13J1, 0RG13JJ, 0RG13K0-0RG13K1, 0RG13KJ, 0RG13Z0-0RG13Z1, 0RG13ZJ, 0RG1470-0RG1471, 0RG147J, 0RG14A0-0RG14A1, 0RG14AJ, 0RG14J0-0RG14J1, 0RG14JJ, 0RG14K0-0RG14K1, 0RG14KJ, 0RG14Z0-0RG14Z1, 0RG14ZJ, 0RG2070-0RG2071, 0RG207J, 0RG20A0-0RG20A1, 0RG20AJ, 0RG20J0-0RG20J1, 0RG20JJ, 0RG20K0-0RG20K1, 0RG20KJ, 0RG20Z0-0RG20Z1, 0RG20ZJ, 0RG2370-0RG2371, 0RG237J, 0RG23A0-0RG23A1, 0RG23AJ, 0RG23J0-0RG23J1, 0RG23JJ, 0RG23K0-0RG23K1, 0RG23KJ, 0RG23Z0-0RG23Z1, 0RG23ZJ, 0RG2470-0RG2471, 0RG247J, 0RG24A0-0RG24A1, 0RG24AJ, 0RG24J0-0RG24J1, 0RG24JJ, 0RG24K0-0RG24K1, 0RG24KJ, 0RG24Z0-0RG24Z1, 0RG24ZJ, 0RG4070-0RG4071, 0RG407J, 0RG40A0-0RG40A1, 0RG40AJ, 0RG40J0-0RG40J1, 0RG40JJ, 0RG40K0-0RG40K1, 0RG40KJ, 0RG40Z0-0RG40Z1, 0RG40ZJ, 0RG4370-0RG4371, 0RG437J, 0RG43A0-0RG43A1, 0RG43AJ, 0RG43J0-0RG43J1, 0RG43JJ, 0RG43K0-0RG43K1, 0RG43KJ, 0RG43Z0-0RG43Z1, 0RG43ZJ, 0RG4470-0RG4471, 0RG447J, 0RG44A0-0RG44A1, 0RG44AJ, 0RG44J0-0RG44J1, 0RG44JJ, 0RG44K0-0RG44K1, 0RG44KJ, 0RG44Z0-0RG44Z1, 0RG44ZJ, XRG0092, XRG00F3, XRG1092, XRG10F3, XRG2092, XRG20F3, XRG4092, XRG40F3
**Elbow procedures**<br>0MB30ZZ, 0MB33ZZ, 0MB34ZZ, 0MB40ZZ, 0MB43ZZ, 0MB44ZZ, 0MD30ZZ, 0MD33ZZ, 0MD34ZZ, 0MD40ZZ, 0MD43ZZ, 0MD44ZZ, 0MQ30ZZ, 0MQ33ZZ, 0MQ34ZZ, 0MQ40ZZ, 0MQ43ZZ, 0MQ44ZZ, 0MT30ZZ, 0MT34ZZ, 0MT40ZZ, 0MT44ZZ, 0RBL0ZZ, 0RBL3ZZ, 0RBL4ZZ, 0RBM0ZZ, 0RBM3ZZ, 0RBM4ZZ, 0RPL08Z, 0RPL0JZ, 0RPL38Z, 0RPL3JZ, 0RPL48Z, 0RPL4JZ, 0RPM08Z, 0RPM0JZ, 0RPM38Z, 0RPM3JZ, 0RPM48Z, 0RPM4JZ, 0RQL0ZZ, 0RQL3ZZ, 0RQL4ZZ, 0RQLXZZ, 0RQM0ZZ, 0RQM3ZZ, 0RQM4ZZ, 0RQMXZZ, 0RRL07Z, 0RRL0JZ, 0RRL0KZ, 0RRM07Z, 0RRM0JZ, 0RRM0KZ, 0RSL3ZZ, 0RSL4ZZ, 0RSLXZZ, 0RSM3ZZ, 0RSM4ZZ, 0RSMXZZ, 0RUL07Z, 0RUL0JZ, 0RUL0KZ, 0RUL37Z, 0RUL3JZ, 0RUL3KZ, 0RUL47Z, 0RUL4JZ, 0RUL4KZ, 0RUM07Z, 0RUM0JZ, 0RUM0KZ, 0RUM37Z, 0RUM3JZ, 0RUM3KZ, 0RUM47Z, 0RUM4JZ, 0RUM4KZ, 0RWL0JZ, 0RWL3JZ, 0RWL4JZ, 0RWM0JZ, 0RWM3JZ, 0RWM4JZ
**Femur repair**<br>0QH604Z, 0QH605Z, 0QH606Z, 0QH60BZ, 0QH60CZ, 0QH60DZ, 0QH634Z, 0QH635Z, 0QH636Z, 0QH63BZ, 0QH63CZ, 0QH63DZ, 0QH644Z, 0QH645Z, 0QH646Z, 0QH64BZ, 0QH64CZ, 0QH64DZ, 0QH704Z, 0QH705Z, 0QH706Z, 0QH70BZ, 0QH70CZ, 0QH70DZ, 0QH734Z, 0QH735Z, 0QH736Z, 0QH73BZ, 0QH73CZ, 0QH73DZ, 0QH744Z, 0QH745Z, 0QH746Z, 0QH74BZ, 0QH74CZ, 0QH74DZ, 0QH804Z, 0QH805Z, 0QH806Z, 0QH80BZ, 0QH80CZ, 0QH80DZ, 0QH834Z, 0QH835Z, 0QH836Z,

0QH83BZ, 0QH83CZ, 0QH83DZ, 0QH844Z, 0QH845Z, 0QH846Z, 0QH84BZ, 0QH84CZ, 0QH84DZ, 0QH904Z, 0QH905Z, 0QH906Z, 0QH90BZ, 0QH90CZ, 0QH90DZ, 0QH934Z, 0QH935Z, 0QH936Z, 0QH93BZ, 0QH93CZ, 0QH93DZ, 0QH944Z, 0QH945Z, 0QH946Z, 0QH94BZ, 0QH94CZ, 0QH94DZ, 0QHB04Z, 0QHB05Z, 0QHB06Z, 0QHB0BZ, 0QHB0CZ, 0QHB0DZ, 0QHB34Z, 0QHB35Z, 0QHB36Z, 0QHB3BZ, 0QHB3CZ, 0QHB3DZ, 0QHB44Z, 0QHB45Z, 0QHB46Z, 0QHB4BZ, 0QHB4CZ, 0QHB4DZ, 0QHC04Z, 0QHC05Z, 0QHC06Z, 0QHC0BZ, 0QHC0CZ, 0QHC0DZ, 0QHC34Z, 0QHC35Z, 0QHC36Z, 0QHC3BZ, 0QHC3CZ, 0QHC3DZ, 0QHC44Z, 0QHC45Z, 0QHC46Z, 0QHC4BZ, 0QHC4CZ, 0QHC4DZ, 0QS604Z, 0QS605Z, 0QS606Z, 0QS60BZ, 0QS60CZ, 0QS60DZ, 0QS60ZZ, 0QS634Z, 0QS635Z, 0QS63BZ, 0QS63CZ, 0QS63DZ, 0QS644Z, 0QS645Z, 0QS64BZ, 0QS64CZ, 0QS64DZ, 0QS704Z, 0QS705Z, 0QS706Z, 0QS70BZ, 0QS70CZ, 0QS70DZ, 0QS70ZZ, 0QS734Z, 0QS735Z, 0QS73BZ, 0QS73CZ, 0QS73DZ, 0QS744Z, 0QS745Z, 0QS74BZ, 0QS74CZ, 0QS74DZ, 0QS804Z, 0QS805Z, 0QS806Z, 0QS80BZ, 0QS80CZ, 0QS80DZ, 0QS80ZZ, 0QS834Z, 0QS835Z, 0QS83BZ, 0QS83CZ, 0QS83DZ, 0QS844Z, 0QS845Z, 0QS84BZ, 0QS84CZ, 0QS84DZ, 0QS904Z, 0QS905Z, 0QS906Z, 0QS90BZ, 0QS90CZ, 0QS90DZ, 0QS90ZZ, 0QS934Z, 0QS935Z, 0QS93BZ, 0QS93CZ, 0QS93DZ, 0QS944Z, 0QS945Z, 0QS94BZ, 0QS94CZ, 0QS94DZ, 0QSB04Z, 0QSB05Z, 0QSB06Z, 0QSB0BZ, 0QSB0CZ, 0QSB0DZ, 0QSB0ZZ, 0QSB34Z, 0QSB35Z, 0QSB3BZ, 0QSB3CZ, 0QSB3DZ, 0QSB44Z, 0QSB45Z, 0QSB4BZ, 0QSB4CZ, 0QSB4DZ, 0QSC04Z, 0QSC05Z, 0QSC06Z, 0QSC0BZ, 0QSC0CZ, 0QSC0DZ, 0QSC0ZZ, 0QSC34Z, 0QSC35Z, 0QSC3BZ, 0QSC3CZ, 0QSC3DZ, 0QSC44Z, 0QSC45Z, 0QSC4BZ, 0QSC4CZ, 0QSC4DZ, 0QU807Z, 0QU80KZ, 0QU837Z, 0QU83KZ, 0QU847Z, 0QU84KZ, 0QU907Z, 0QU90KZ, 0QU937Z, 0QU93KZ, 0QU947Z, 0QU94KZ, 0QUB07Z, 0QUB0KZ, 0QUB37Z, 0QUB3KZ, 0QUB47Z, 0QUB4KZ, 0QUC07Z, 0QUC0KZ, 0QUC37Z, 0QUC3KZ, 0QUC47Z, 0QUC4KZ
**Femur revision**<br>0QP804Z, 0QP805Z, 0QP807Z, 0QP80JZ, 0QP80KZ, 0QP834Z, 0QP835Z, 0QP837Z, 0QP83JZ, 0QP83KZ, 0QP844Z, 0QP845Z, 0QP847Z, 0QP84JZ, 0QP84KZ, 0QP904Z, 0QP905Z, 0QP907Z, 0QP90JZ, 0QP90KZ, 0QP934Z, 0QP935Z, 0QP937Z, 0QP93JZ, 0QP93KZ, 0QP944Z, 0QP945Z, 0QP947Z, 0QP94JZ, 0QP94KZ, 0QPB04Z, 0QPB05Z, 0QPB07Z, 0QPB0JZ, 0QPB0KZ, 0QPB34Z, 0QPB35Z, 0QPB37Z, 0QPB3JZ, 0QPB3KZ, 0QPB44Z, 0QPB45Z, 0QPB47Z, 0QPB4JZ, 0QPB4KZ, 0QPC04Z, 0QPC05Z, 0QPC07Z, 0QPC0JZ, 0QPC0KZ, 0QPC34Z, 0QPC35Z, 0QPC37Z, 0QPC3JZ, 0QPC3KZ, 0QPC44Z, 0QPC45Z, 0QPC47Z, 0QPC4JZ, 0QPC4KZ
**Foot procedures**<br>0LMV0ZZ, 0LMV4ZZ, 0LMW0ZZ, 0LMW4ZZ, 0LQV0ZZ, 0LQV3ZZ, 0LQV4ZZ, 0LQW0ZZ, 0LQW3ZZ, 0LQW4ZZ, 0MBS0ZZ, 0MBS3ZZ, 0MBS4ZZ, 0MBT0ZZ, 0MBT3ZZ, 0MBT4ZZ, 0MDS0ZZ, 0MDS3ZZ, 0MDS4ZZ, 0MDT0ZZ, 0MDT3ZZ, 0MDT4ZZ, 0MTS0ZZ, 0MTS4ZZ, 0MTT0ZZ, 0MTT4ZZ, 0QBL0ZX, 0QBL0ZZ, 0QBL4ZX, 0QBL4ZZ, 0QBM0ZX, 0QBM0ZZ, 0QBM4ZX, 0QBM4ZZ, 0QBN0ZX, 0QBN0ZZ, 0QBN4ZX, 0QBN4ZZ, 0QBP0ZX, 0QBP0ZZ, 0QBP4ZX, 0QBP4ZZ, 0QBQ0ZX, 0QBQ0ZZ, 0QBQ3ZZ, 0QBQ4ZX, 0QBQ4ZZ, 0QBR0ZX, 0QBR0ZZ, 0QBR3ZZ, 0QBR4ZX, 0QBR4ZZ, 0QPL04Z, 0QPL05Z, 0QPL34Z, 0QPL35Z, 0QPL44Z, 0QPL45Z, 0QPM04Z, 0QPM05Z, 0QPM34Z, 0QPM35Z, 0QPM44Z, 0QPM45Z, 0QPN04Z, 0QPN05Z, 0QPN34Z, 0QPN35Z, 0QPN44Z, 0QPN45Z, 0QPP04Z, 0QPP05Z, 0QPP34Z, 0QPP35Z, 0QPP44Z, 0QPP45Z, 0QPQ04Z, 0QPQ05Z, 0QPQ07Z, 0QPQ0JZ, 0QPQ0KZ, 0QPQ34Z, 0QPQ35Z, 0QPQ37Z, 0QPQ3JZ, 0QPQ3KZ, 0QPQ44Z, 0QPQ45Z, 0QPQ47Z, 0QPQ4JZ, 0QPQ4KZ, 0QPR04Z, 0QPR05Z, 0QPR07Z, 0QPR0JZ, 0QPR0KZ, 0QPR34Z, 0QPR35Z, 0QPR37Z, 0QPR3JZ, 0QPR3KZ, 0QPR44Z, 0QPR45Z, 0QPR47Z, 0QPR4JZ, 0QPR4KZ, 0QSL04Z, 0QSL34Z, 0QSL44Z, 0QSM04Z, 0QSM34Z, 0QSM44Z, 0QSN042, 0QSN04Z, 0QSN342, 0QSN34Z, 0QSN442, 0QSN44Z, 0QSP042, 0QSP04Z, 0QSP342, 0QSP34Z, 0QSP442, 0QSP44Z, 0QSQ04Z, 0QSQ34Z, 0QSQ44Z, 0QSR04Z, 0QSR34Z, 0QSR44Z, 0QTL0ZZ, 0QTM0ZZ, 0QTN0ZZ, 0QTP0ZZ, 0QTQ0ZZ, 0QTR0ZZ, 0QUL07Z, 0QUL37Z, 0QUL47Z, 0QUM07Z, 0QUM37Z, 0QUM47Z, 0QUN07Z, 0QUN37Z, 0QUN47Z, 0QUP07Z, 0QUP37Z, 0QUP47Z, 0QUQ07Z, 0QUQ37Z, 0QUQ47Z, 0QUR07Z, 0QUR37Z, 0QUR47Z, 0SBH0ZZ, 0SBH3ZZ, 0SBH4ZZ, 0SBJ0ZZ, 0SBJ3ZZ, 0SBJ4ZZ, 0SBK0ZZ, 0SBK3ZZ, 0SBK4ZZ, 0SBL0ZZ, 0SBL3ZZ, 0SBL4ZZ, 0SBM0ZZ, 0SBM3ZZ, 0SBM4ZZ, 0SBN0ZZ, 0SBN3ZZ, 0SBN4ZZ, 0SBP0ZZ, 0SBP3ZZ, 0SBP4ZZ, 0SBQ0ZZ, 0SBQ3ZZ, 0SBQ4ZZ, 0SGH04Z, 0SGH05Z, 0SGH07Z, 0SGH0JZ, 0SGH0KZ, 0SGH0ZZ, 0SGH34Z, 0SGH35Z, 0SGH37Z, 0SGH3JZ, 0SGH3KZ, 0SGH3ZZ, 0SGH44Z, 0SGH45Z, 0SGH47Z, 0SGH4JZ, 0SGH4KZ, 0SGH4ZZ, 0SGJ04Z, 0SGJ05Z, 0SGJ07Z, 0SGJ0JZ, 0SGJ0KZ, 0SGJ0ZZ, 0SGJ34Z, 0SGJ35Z, 0SGJ37Z, 0SGJ3JZ, 0SGJ3KZ, 0SGJ3ZZ, 0SGJ44Z, 0SGJ45Z, 0SGJ47Z, 0SGJ4JZ, 0SGJ4KZ, 0SGJ4ZZ, 0SGK04Z, 0SGK05Z, 0SGK07Z, 0SGK0JZ, 0SGK0KZ, 0SGK0ZZ, 0SGK34Z, 0SGK35Z, 0SGK37Z, 0SGK3JZ, 0SGK3KZ,

0SGK3ZZ, 0SGK44Z, 0SGK45Z, 0SGK47Z, 0SGK4JZ, 0SGK4KZ, 0SGK4ZZ, 0SGL04Z, 0SGL05Z, 0SGL07Z, 0SGL0JZ, 0SGL0KZ, 0SGL0ZZ, 0SGL34Z, 0SGL35Z, 0SGL37Z, 0SGL3JZ, 0SGL3KZ, 0SGL3ZZ, 0SGL44Z, 0SGL45Z, 0SGL47Z, 0SGL4JZ, 0SGL4KZ, 0SGL4ZZ, 0SGM04Z, 0SGM05Z, 0SGM07Z, 0SGM0JZ, 0SGM0KZ, 0SGM0ZZ, 0SGM34Z, 0SGM35Z, 0SGM37Z, 0SGM3JZ, 0SGM3KZ, 0SGM3ZZ, 0SGM44Z, 0SGM45Z, 0SGM47Z, 0SGM4JZ, 0SGM4KZ, 0SGM4ZZ, 0SGN04Z, 0SGN05Z, 0SGN07Z, 0SGN0JZ, 0SGN0KZ, 0SGN0ZZ, 0SGN34Z, 0SGN35Z, 0SGN37Z, 0SGN3JZ, 0SGN3KZ, 0SGN3ZZ, 0SGN44Z, 0SGN45Z, 0SGN47Z, 0SGN4JZ, 0SGN4KZ, 0SGN4ZZ, 0SGP04Z, 0SGP05Z, 0SGP07Z, 0SGP0JZ, 0SGP0KZ, 0SGP0ZZ, 0SGP34Z, 0SGP35Z, 0SGP37Z, 0SGP3JZ, 0SGP3KZ, 0SGP3ZZ, 0SGP44Z, 0SGP45Z, 0SGP47Z, 0SGP4JZ, 0SGP4KZ, 0SGP4ZZ, 0SGQ04Z, 0SGQ05Z, 0SGQ07Z, 0SGQ0JZ, 0SGQ0KZ, 0SGQ0ZZ, 0SGQ34Z, 0SGQ35Z, 0SGQ37Z, 0SGQ3JZ, 0SGQ3KZ, 0SGQ3ZZ, 0SGQ44Z, 0SGQ45Z, 0SGQ47Z, 0SGQ4JZ, 0SGQ4KZ, 0SGQ4ZZ, 0SPH08Z, 0SPH38Z, 0SPH48Z, 0SPJ08Z, 0SPJ38Z, 0SPJ48Z, 0SPK08Z, 0SPK0JZ, 0SPK38Z, 0SPK3JZ, 0SPK48Z, 0SPK4JZ, 0SPL08Z, 0SPL0JZ, 0SPL38Z, 0SPL3JZ, 0SPL48Z, 0SPL4JZ, 0SPM08Z, 0SPM0JZ, 0SPM38Z, 0SPM3JZ, 0SPM48Z, 0SPM4JZ, 0SPN08Z, 0SPN0JZ, 0SPN38Z, 0SPN3JZ, 0SPN48Z, 0SPN4JZ, 0SPP08Z, 0SPP0JZ, 0SPP38Z, 0SPP3JZ, 0SPP48Z, 0SPP4JZ, 0SPQ08Z, 0SPQ0JZ, 0SPQ38Z, 0SPQ3JZ, 0SPQ48Z, 0SPQ4JZ, 0STH0ZZ, 0STJ0ZZ, 0STK0ZZ, 0STL0ZZ, 0STM0ZZ, 0STN0ZZ, 0STP0ZZ, 0STQ0ZZ
**Forearm procedures**<br>0PBH0ZX, 0PBH0ZZ, 0PBH3ZZ, 0PBH4ZX, 0PBH4ZZ, 0PBJ0ZX, 0PBJ0ZZ, 0PBJ3ZZ, 0PBJ4ZX, 0PBJ4ZZ, 0PBK0ZX, 0PBK0ZZ, 0PBK3ZZ, 0PBK4ZX, 0PBK4ZZ, 0PBL0ZX, 0PBL0ZZ, 0PBL3ZZ, 0PBL4ZX, 0PBL4ZZ, 0PHH05Z, 0PHH0BZ, 0PHH0CZ, 0PHH0DZ, 0PHH35Z, 0PHH3BZ, 0PHH3CZ, 0PHH3DZ, 0PHH45Z, 0PHH4BZ, 0PHH4CZ, 0PHH4DZ, 0PHJ05Z, 0PHJ0BZ, 0PHJ0CZ, 0PHJ0DZ, 0PHJ35Z, 0PHJ3BZ, 0PHJ3CZ, 0PHJ3DZ, 0PHJ45Z, 0PHJ4BZ, 0PHJ4CZ, 0PHJ4DZ, 0PHK05Z, 0PHK0BZ, 0PHK0CZ, 0PHK0DZ, 0PHK35Z, 0PHK3BZ, 0PHK3CZ, 0PHK3DZ, 0PHK45Z, 0PHK4BZ, 0PHK4CZ, 0PHK4DZ, 0PHL05Z, 0PHL0BZ, 0PHL0CZ, 0PHL0DZ, 0PHL35Z, 0PHL3BZ, 0PHL3CZ, 0PHL3DZ, 0PHL45Z, 0PHL4BZ, 0PHL4CZ, 0PHL4DZ, 0PPH04Z, 0PPH05Z, 0PPH07Z, 0PPH0JZ, 0PPH0KZ, 0PPH34Z, 0PPH35Z, 0PPH37Z, 0PPH3JZ, 0PPH3KZ, 0PPH44Z, 0PPH45Z, 0PPH47Z, 0PPH4JZ, 0PPH4KZ, 0PPJ04Z, 0PPJ05Z, 0PPJ07Z, 0PPJ0JZ, 0PPJ0KZ, 0PPJ34Z, 0PPJ35Z, 0PPJ37Z, 0PPJ3JZ, 0PPJ3KZ, 0PPJ44Z, 0PPJ45Z, 0PPJ47Z, 0PPJ4JZ, 0PPJ4KZ, 0PPK04Z, 0PPK05Z, 0PPK07Z, 0PPK0JZ, 0PPK0KZ, 0PPK34Z, 0PPK35Z, 0PPK37Z, 0PPK3JZ, 0PPK3KZ, 0PPK44Z, 0PPK45Z, 0PPK47Z, 0PPK4JZ, 0PPK4KZ, 0PPL04Z, 0PPL05Z, 0PPL07Z, 0PPL0JZ, 0PPL0KZ, 0PPL34Z, 0PPL35Z, 0PPL37Z, 0PPL3JZ, 0PPL3KZ, 0PPL44Z, 0PPL45Z, 0PPL47Z, 0PPL4JZ, 0PPL4KZ, 0PSH04Z, 0PSH05Z, 0PSH06Z, 0PSH0BZ, 0PSH0CZ, 0PSH0DZ, 0PSH0ZZ, 0PSH34Z, 0PSH35Z, 0PSH3BZ, 0PSH3CZ, 0PSH3DZ, 0PSH44Z, 0PSH45Z, 0PSH4BZ, 0PSH4CZ, 0PSH4DZ, 0PSJ04Z, 0PSJ05Z, 0PSJ06Z, 0PSJ0BZ, 0PSJ0CZ, 0PSJ0DZ, 0PSJ0ZZ, 0PSJ34Z, 0PSJ35Z, 0PSJ3BZ, 0PSJ3CZ, 0PSJ3DZ, 0PSJ44Z, 0PSJ45Z, 0PSJ4BZ, 0PSJ4CZ, 0PSJ4DZ, 0PSK04Z, 0PSK05Z, 0PSK06Z, 0PSK0BZ, 0PSK0CZ, 0PSK0DZ, 0PSK0ZZ, 0PSK34Z, 0PSK35Z, 0PSK3BZ, 0PSK3CZ, 0PSK3DZ, 0PSK44Z, 0PSK45Z, 0PSK4BZ, 0PSK4CZ, 0PSK4DZ, 0PSL04Z, 0PSL05Z, 0PSL06Z, 0PSL0BZ, 0PSL0CZ, 0PSL0DZ, 0PSL0ZZ, 0PSL34Z, 0PSL35Z, 0PSL3BZ, 0PSL3CZ, 0PSL3DZ, 0PSL44Z, 0PSL45Z, 0PSL4BZ, 0PSL4CZ, 0PSL4DZ, 0PTH0ZZ, 0PTJ0ZZ, 0PTK0ZZ, 0PTL0ZZ, 0PUH07Z, 0PUH0KZ, 0PUH37Z, 0PUH3KZ, 0PUH47Z, 0PUH4KZ, 0PUJ07Z, 0PUJ0KZ, 0PUJ37Z, 0PUJ3KZ, 0PUJ47Z, 0PUJ4KZ, 0PUK07Z, 0PUK0KZ, 0PUK37Z, 0PUK3KZ, 0PUK47Z, 0PUK4KZ, 0PUL07Z, 0PUL0KZ, 0PUL37Z, 0PUL3KZ, 0PUL47Z, 0PUL4KZ
**Hip revision**<br>0QP404Z, 0QP407Z, 0QP40JZ, 0QP40KZ, 0QP434Z, 0QP437Z, 0QP43JZ, 0QP43KZ, 0QP444Z, 0QP447Z, 0QP44JZ, 0QP44KZ, 0QP504Z, 0QP507Z, 0QP50JZ, 0QP50KZ, 0QP534Z, 0QP537Z, 0QP53JZ, 0QP53KZ, 0QP544Z, 0QP547Z, 0QP54JZ, 0QP54KZ, 0QP604Z, 0QP605Z, 0QP607Z, 0QP60JZ, 0QP60KZ, 0QP634Z, 0QP635Z, 0QP637Z, 0QP63JZ, 0QP63KZ, 0QP644Z, 0QP645Z, 0QP647Z, 0QP64JZ, 0QP64KZ, 0QP704Z, 0QP705Z, 0QP707Z, 0QP70JZ, 0QP70KZ, 0QP734Z, 0QP735Z, 0QP737Z, 0QP73JZ, 0QP73KZ, 0QP744Z, 0QP745Z, 0QP747Z, 0QP74JZ, 0QP74KZ, 0SP904Z, 0SP905Z, 0SP907Z, 0SP908Z, 0SP909Z, 0SP90BZ, 0SP90JZ, 0SP90KZ, 0SP934Z, 0SP935Z, 0SP937Z, 0SP938Z, 0SP93JZ, 0SP93KZ, 0SP944Z, 0SP945Z, 0SP947Z, 0SP948Z, 0SP94JZ, 0SP94KZ, 0SPA0JZ, 0SPA3JZ, 0SPA4JZ, 0SPB04Z, 0SPB05Z, 0SPB07Z, 0SPB08Z, 0SPB09Z, 0SPB0BZ, 0SPB0JZ, 0SPB0KZ, 0SPB34Z, 0SPB35Z, 0SPB37Z, 0SPB38Z, 0SPB3JZ, 0SPB3KZ, 0SPB44Z, 0SPB45Z, 0SPB47Z, 0SPB48Z, 0SPB4JZ, 0SPB4KZ, 0SPE0JZ, 0SPE3JZ, 0SPE4JZ, 0SPR0JZ, 0SPR3JZ, 0SPR4JZ, 0SPS0JZ, 0SPS3JZ, 0SPS4JZ, 0SW904Z, 0SW905Z,

0SW907Z, 0SW908Z, 0SW909Z, 0SW90BZ, 0SW90JZ, 0SW90KZ, 0SW934Z, 0SW935Z, 0SW937Z, 0SW938Z, 0SW93JZ, 0SW93KZ, 0SW944Z, 0SW945Z, 0SW947Z, 0SW948Z, 0SW94JZ, 0SW94KZ, 0SWA0JZ, 0SWA3JZ, 0SWA4JZ, 0SWB04Z, 0SWB05Z, 0SWB07Z, 0SWB08Z, 0SWB09Z, 0SWB0BZ, 0SWB0JZ, 0SWB0KZ, 0SWB34Z, 0SWB35Z, 0SWB37Z, 0SWB38Z, 0SWB3JZ, 0SWB3KZ, 0SWB44Z, 0SWB45Z, 0SWB47Z, 0SWB48Z, 0SWB4JZ, 0SWB4KZ, 0SWE0JZ, 0SWE3JZ, 0SWE4JZ, 0SWR0JZ, 0SWR3JZ, 0SWR4JZ, 0SWS0JZ, 0SWS3JZ, 0SWS4JZ
**Humerus procedures**<br>0PBC0ZX, 0PBC0ZZ, 0PBC3ZZ, 0PBC4ZX, 0PBC4ZZ, 0PBD0ZX, 0PBD0ZZ, 0PBD3ZZ, 0PBD4ZX, 0PBD4ZZ, 0PBF0ZX, 0PBF0ZZ, 0PBF3ZZ, 0PBF4ZX, 0PBF4ZZ, 0PBG0ZX, 0PBG0ZZ, 0PBG3ZZ, 0PBG4ZX, 0PBG4ZZ, 0PHC04Z, 0PHC05Z, 0PHC06Z, 0PHC0BZ, 0PHC0CZ, 0PHC0DZ, 0PHC34Z, 0PHC35Z, 0PHC36Z, 0PHC3BZ, 0PHC3CZ, 0PHC3DZ, 0PHC44Z, 0PHC45Z, 0PHC46Z, 0PHC4BZ, 0PHC4CZ, 0PHC4DZ, 0PHD04Z, 0PHD05Z, 0PHD06Z, 0PHD0BZ, 0PHD0CZ, 0PHD0DZ, 0PHD34Z, 0PHD35Z, 0PHD36Z, 0PHD3BZ, 0PHD3CZ, 0PHD3DZ, 0PHD44Z, 0PHD45Z, 0PHD46Z, 0PHD4BZ, 0PHD4CZ, 0PHD4DZ, 0PHF04Z, 0PHF05Z, 0PHF06Z, 0PHF0BZ, 0PHF0CZ, 0PHF0DZ, 0PHF34Z, 0PHF35Z, 0PHF36Z, 0PHF3BZ, 0PHF3CZ, 0PHF3DZ, 0PHF44Z, 0PHF45Z, 0PHF46Z, 0PHF4BZ, 0PHF4CZ, 0PHF4DZ, 0PHG04Z, 0PHG05Z, 0PHG06Z, 0PHG0BZ, 0PHG0CZ, 0PHG0DZ, 0PHG34Z, 0PHG35Z, 0PHG36Z, 0PHG3BZ, 0PHG3CZ, 0PHG3DZ, 0PHG44Z, 0PHG45Z, 0PHG46Z, 0PHG4BZ, 0PHG4CZ, 0PHG4DZ, 0PPF04Z, 0PPF05Z, 0PPF07Z, 0PPF0JZ, 0PPF0KZ, 0PPF34Z, 0PPF35Z, 0PPF37Z, 0PPF3JZ, 0PPF3KZ, 0PPF44Z, 0PPF45Z, 0PPF47Z, 0PPF4JZ, 0PPF4KZ, 0PPG04Z, 0PPG05Z, 0PPG07Z, 0PPG0JZ, 0PPG0KZ, 0PPG34Z, 0PPG35Z, 0PPG37Z, 0PPG3JZ, 0PPG3KZ, 0PPG44Z, 0PPG45Z, 0PPG47Z, 0PPG4JZ, 0PPG4KZ, 0PSC04Z, 0PSC05Z, 0PSC06Z, 0PSC0BZ, 0PSC0CZ, 0PSC0DZ, 0PSC0ZZ, 0PSC34Z, 0PSC35Z, 0PSC3BZ, 0PSC3CZ, 0PSC3DZ, 0PSC44Z, 0PSC45Z, 0PSC4BZ, 0PSC4CZ, 0PSC4DZ, 0PSD04Z, 0PSD05Z, 0PSD06Z, 0PSD0BZ, 0PSD0CZ, 0PSD0DZ, 0PSD0ZZ, 0PSD34Z, 0PSD35Z, 0PSD3BZ, 0PSD3CZ, 0PSD3DZ, 0PSD44Z, 0PSD45Z, 0PSD4BZ, 0PSD4CZ, 0PSD4DZ, 0PSF04Z, 0PSF05Z, 0PSF06Z, 0PSF0BZ, 0PSF0CZ, 0PSF0DZ, 0PSF0ZZ, 0PSF34Z, 0PSF35Z, 0PSF3BZ, 0PSF3CZ, 0PSF3DZ, 0PSF44Z, 0PSF45Z, 0PSF4BZ, 0PSF4CZ, 0PSF4DZ, 0PSG04Z, 0PSG05Z, 0PSG06Z, 0PSG0BZ, 0PSG0CZ, 0PSG0DZ, 0PSG0ZZ, 0PSG34Z, 0PSG35Z, 0PSG3BZ, 0PSG3CZ, 0PSG3DZ, 0PSG44Z, 0PSG45Z, 0PSG4BZ, 0PSG4CZ, 0PSG4DZ, 0PTC0ZZ, 0PTD0ZZ, 0PTF0ZZ, 0PTG0ZZ, 0PUC07Z, 0PUC0KZ, 0PUC37Z, 0PUC3KZ, 0PUC47Z, 0PUC4KZ, 0PUD07Z, 0PUD0KZ, 0PUD37Z, 0PUD3KZ, 0PUD47Z, 0PUD4KZ, 0PUF07Z, 0PUF0KZ, 0PUF37Z, 0PUF3KZ, 0PUF47Z, 0PUF4KZ, 0PUG07Z, 0PUG0KZ, 0PUG37Z, 0PUG3KZ, 0PUG47Z, 0PUG4KZ
**Knee revision**<br>0QPD04Z, 0QPD05Z, 0QPD07Z, 0QPD0JZ, 0QPD0KZ, 0QPD34Z, 0QPD35Z, 0QPD37Z, 0QPD3JZ, 0QPD3KZ, 0QPD44Z, 0QPD45Z, 0QPD47Z, 0QPD4JZ, 0QPD4KZ, 0QPF04Z, 0QPF05Z, 0QPF07Z, 0QPF0JZ, 0QPF0KZ, 0QPF34Z, 0QPF35Z, 0QPF37Z, 0QPF3JZ, 0QPF3KZ, 0QPF44Z, 0QPF45Z, 0QPF47Z, 0QPF4JZ, 0QPF4KZ, 0SPC08Z, 0SPC09Z, 0SPC0JC, 0SPC0JZ, 0SPC38Z, 0SPC3JC, 0SPC3JZ, 0SPC48Z, 0SPC4JC, 0SPC4JZ, 0SPD08Z, 0SPD09Z, 0SPD0JC, 0SPD0JZ, 0SPD38Z, 0SPD3JC, 0SPD3JZ, 0SPD48Z, 0SPD4JC, 0SPD4JZ, 0SPT0JZ, 0SPT3JZ, 0SPT4JZ, 0SPU0JZ, 0SPU3JZ, 0SPU4JZ, 0SPV0JZ, 0SPV3JZ, 0SPV4JZ, 0SPW0JZ, 0SPW3JZ, 0SPW4JZ, 0SWC0JC, 0SWC0JZ, 0SWC3JC, 0SWC3JZ, 0SWC4JC, 0SWC4JZ, 0SWD0JC, 0SWD0JZ, 0SWD3JC, 0SWD3JZ, 0SWD4JC, 0SWD4JZ, 0SWT0JZ, 0SWT3JZ, 0SWT4JZ, 0SWU0JZ, 0SWU3JZ, 0SWU4JZ, 0SWV0JZ, 0SWV3JZ, 0SWV4JZ, 0SWW0JZ, 0SWW3JZ, 0SWW4JZ
**Ostectomy, femur**<br>0QB60ZX, 0QB60ZZ, 0QB63ZZ, 0QB64ZX, 0QB64ZZ, 0QB70ZX, 0QB70ZZ, 0QB73ZZ, 0QB74ZX, 0QB74ZZ, 0QB80ZX, 0QB80ZZ, 0QB83ZZ, 0QB84ZX, 0QB84ZZ, 0QB90ZX, 0QB90ZZ, 0QB93ZZ, 0QB94ZX, 0QB94ZZ, 0QBB0ZX, 0QBB0ZZ, 0QBB3ZZ, 0QBB4ZX, 0QBB4ZZ, 0QBC0ZX, 0QBC0ZZ, 0QBC3ZZ, 0QBC4ZX, 0QBC4ZZ, 0QT60ZZ, 0QT70ZZ, 0QT80ZZ, 0QT90ZZ, 0QTB0ZZ, 0QTC0ZZ
**Ostectomy, knee**<br>0QBD0ZX, 0QBD0ZZ, 0QBD3ZZ, 0QBD4ZX, 0QBD4ZZ, 0QBF0ZX, 0QBF0ZZ, 0QBF3ZZ, 0QBF4ZX, 0QBF4ZZ, 0QTD0ZZ, 0QTF0ZZ
**Ostectomy, tibia/fibula**<br>0QBG0ZZ, 0QBG3ZZ, 0QBG4ZZ, 0QBH0ZZ, 0QBH3ZZ, 0QBH4ZZ, 0QBJ0ZZ, 0QBJ3ZZ, 0QBJ4ZZ, 0QBK0ZZ, 0QBK3ZZ, 0QBK4ZZ, 0QTG0ZZ, 0QTH0ZZ, 0QTJ0ZZ, 0QTK0ZZ

**Other fracture repair**<br>0PS004Z, 0PS104Z, 0PS204Z, 0PS504Z, 0PS604Z, 0PS704Z, 0PS804Z, 0PS904Z, 0PSB04Z, 0QS204Z, 0QS234Z, 0QS244Z, 0QS304Z, 0QS334Z, 0QS344Z
**Other hip procedures**<br>0LMJ0ZZ, 0LMJ4ZZ, 0LMK0ZZ, 0LMK4ZZ, 0LQJ0ZZ, 0LQJ3ZZ, 0LQJ4ZZ, 0LQK0ZZ, 0LQK3ZZ, 0LQK4ZZ, 0MBL0ZZ, 0MBL3ZZ, 0MBL4ZZ, 0MBM0ZZ, 0MBM3ZZ, 0MBM4ZZ, 0MDL0ZZ, 0MDL3ZZ, 0MDL4ZZ, 0MDM0ZZ, 0MDM3ZZ, 0MDM4ZZ, 0MTL0ZZ, 0MTL4ZZ, 0MTM0ZZ, 0MTM4ZZ, 0QB40ZZ, 0QB44ZZ, 0QB50ZZ, 0QB54ZZ, 0QS404Z, 0QS434Z, 0QS444Z, 0QS504Z, 0QS534Z, 0QS544Z, 0QU607Z, 0QU60KZ, 0QU637Z, 0QU63KZ, 0QU647Z, 0QU64KZ, 0QU707Z, 0QU70KZ, 0QU737Z, 0QU73KZ, 0QU747Z, 0QU74KZ, 0S990ZZ, 0S9B0ZZ, 0SB90ZZ, 0SB93ZZ, 0SB94ZZ, 0SBB0ZZ, 0SBB3ZZ, 0SBB4ZZ, 0SC90ZZ, 0SC93ZZ, 0SC94ZZ, 0SCB0ZZ, 0SCB3ZZ, 0SCB4ZZ, 0SH904Z, 0SH905Z, 0SH934Z, 0SH935Z, 0SH944Z, 0SH945Z, 0SHB04Z, 0SHB05Z, 0SHB34Z, 0SHB35Z, 0SHB44Z, 0SHB45Z, 0SQ90ZZ, 0SQ93ZZ, 0SQ94ZZ, 0SQ9XZZ, 0SQB0ZZ, 0SQB3ZZ, 0SQB4ZZ, 0SQBXZZ, 0ST90ZZ, 0STB0ZZ, 0SU907Z, 0SU909Z, 0SU90JZ, 0SU90KZ, 0SU937Z, 0SU93JZ, 0SU93KZ, 0SU947Z, 0SU94JZ, 0SU94KZ, 0SUA09Z, 0SUB07Z, 0SUB09Z, 0SUB0JZ, 0SUB0KZ, 0SUB37Z, 0SUB3JZ, 0SUB3KZ, 0SUB47Z, 0SUB4JZ, 0SUB4KZ, 0SUE09Z, 0SUR09Z, 0SUS09Z
**Other knee procedures**<br>0LMQ0ZZ, 0LMQ4ZZ, 0LMR0ZZ, 0LMR4ZZ, 0LQQ0ZZ, 0LQQ3ZZ, 0LQQ4ZZ, 0LQR0ZZ, 0LQR3ZZ, 0LQR4ZZ, 0LSQ0ZZ, 0LSQ4ZZ, 0LSR0ZZ, 0LSR4ZZ, 0MBN0ZZ, 0MBN3ZZ, 0MBN4ZZ, 0MBP0ZZ, 0MBP3ZZ, 0MBP4ZZ, 0MDN0ZZ, 0MDN3ZZ, 0MDN4ZZ, 0MDP0ZZ, 0MDP3ZZ, 0MDP4ZZ, 0MQN0ZZ, 0MQN3ZZ, 0MQN4ZZ, 0MQP0ZZ, 0MQP3ZZ, 0MQP4ZZ, 0MTN0ZZ, 0MTN4ZZ, 0MTP0ZZ, 0MTP4ZZ, 0QQD0ZZ, 0QQD4ZZ, 0QQDXZZ, 0QQF0ZZ, 0QQF4ZZ, 0QQFXZZ, 0QSD04Z, 0QSD34Z, 0QSD44Z, 0QSF04Z, 0QSF34Z, 0QSF44Z, 0QUD07Z, 0QUD0JZ, 0QUD0KZ, 0QUD37Z, 0QUD3JZ, 0QUD3KZ, 0QUD47Z, 0QUD4JZ, 0QUD4KZ, 0QUF07Z, 0QUF0JZ, 0QUF0KZ, 0QUF37Z, 0QUF3JZ, 0QUF3KZ, 0QUF47Z, 0QUF4JZ, 0QUF4KZ, 0SBC0ZZ, 0SBC3ZZ, 0SBC4ZZ, 0SBD0ZZ, 0SBD3ZZ, 0SBD4ZZ, 0SGC04Z, 0SGC05Z, 0SGC07Z, 0SGC0JZ, 0SGC0KZ, 0SGC0ZZ, 0SGC34Z, 0SGC35Z, 0SGC37Z, 0SGC3JZ, 0SGC3KZ, 0SGC3ZZ, 0SGC44Z, 0SGC45Z, 0SGC47Z, 0SGC4JZ, 0SGC4KZ, 0SGC4ZZ, 0SGD04Z, 0SGD05Z, 0SGD07Z, 0SGD0JZ, 0SGD0KZ, 0SGD0ZZ, 0SGD34Z, 0SGD35Z, 0SGD37Z, 0SGD3JZ, 0SGD3KZ, 0SGD3ZZ, 0SGD44Z, 0SGD45Z, 0SGD47Z, 0SGD4JZ, 0SGD4KZ, 0SGD4ZZ, 0SQC0ZZ, 0SQC3ZZ, 0SQC4ZZ, 0SQCXZZ, 0SQD0ZZ, 0SQD3ZZ, 0SQD4ZZ, 0SQDXZZ, 0STC0ZZ, 0STD0ZZ, 0SUC07Z, 0SUC09C, 0SUC09Z, 0SUC0JZ, 0SUC0KZ, 0SUC37Z, 0SUC3JZ, 0SUC3KZ, 0SUC47Z, 0SUC4JZ, 0SUC4KZ, 0SUD07Z, 0SUD09C, 0SUD09Z, 0SUD0JZ, 0SUD0KZ, 0SUD37Z, 0SUD3JZ, 0SUD3KZ, 0SUD47Z, 0SUD4JZ, 0SUD4KZ, 0SUT09Z, 0SUU09Z, 0SUV09Z, 0SUW09Z
**Other shoulder procedures**<br>0LM10ZZ, 0LM14ZZ, 0LM20ZZ, 0LM24ZZ, 0LQ10ZZ, 0LQ13ZZ, 0LQ14ZZ, 0LQ20ZZ, 0LQ23ZZ, 0LQ24ZZ, 0MB10ZZ, 0MB13ZZ, 0MB14ZZ, 0MB20ZZ, 0MB23ZZ, 0MB24ZZ, 0MD10ZZ, 0MD13ZZ, 0MD14ZZ, 0MD20ZZ, 0MD23ZZ, 0MD24ZZ, 0MT10ZZ, 0MT14ZZ, 0MT20ZZ, 0MT24ZZ, 0RBJ0ZZ, 0RBJ3ZZ, 0RBJ4ZZ, 0RBK0ZZ, 0RBK3ZZ, 0RBK4ZZ, 0RQJ0ZZ, 0RQJ3ZZ, 0RQJ4ZZ, 0RQJXZZ, 0RQK0ZZ, 0RQK3ZZ, 0RQK4ZZ, 0RQKXZZ, 0RSJ3ZZ, 0RSJ4ZZ, 0RSJXZZ, 0RSK3ZZ, 0RSK4ZZ, 0RSKXZZ, 0RUJ07Z, 0RUJ0JZ, 0RUJ0KZ, 0RUJ37Z, 0RUJ3JZ, 0RUJ3KZ, 0RUJ47Z, 0RUJ4JZ, 0RUJ4KZ, 0RUK07Z, 0RUK0JZ, 0RUK0KZ, 0RUK37Z, 0RUK3JZ, 0RUK3KZ, 0RUK47Z, 0RUK4JZ, 0RUK4KZ
**Other spinal procedures**<br>009T0ZZ, 009T4ZZ, 009U0ZZ, 009W0ZZ, 009W4ZZ, 009X0ZZ, 009X4ZZ, 009Y0ZZ, 009Y4ZZ, 0MBC0ZZ, 0MBC3ZZ, 0MBC4ZZ, 0MBD0ZZ, 0MBD3ZZ, 0MBD4ZZ, 0MDC0ZZ, 0MDC3ZZ, 0MDC4ZZ, 0MDD0ZZ, 0MDD3ZZ, 0MDD4ZZ, 0MTC0ZZ, 0MTC4ZZ, 0MTD0ZZ, 0MTD4ZZ, 0PB30ZX, 0PB30ZZ, 0PB34ZX, 0PB34ZZ, 0PB40ZX, 0PB40ZZ, 0PB44ZX, 0PB44ZZ, 0PR307Z, 0PR30JZ, 0PR337Z, 0PR33JZ, 0PR347Z, 0PR34JZ, 0PR407Z, 0PR40JZ, 0PR437Z, 0PR43JZ, 0PR447Z, 0PR44JZ, 0PU30JZ, 0PU30KZ, 0PU34JZ, 0PU34KZ, 0PU407Z, 0PU40JZ, 0PU40KZ, 0PU437Z, 0PU44JZ, 0PU44KZ, 0QB00ZX, 0QB00ZZ, 0QB04ZX, 0QB04ZZ, 0QB10ZZ, 0QB14ZZ, 0QBS0ZZ, 0QBS4ZZ, 0QU007Z, 0QU00JZ, 0QU00KZ, 0QU037Z, 0QU03JZ, 0QU047Z, 0QU04JZ, 0QU04KZ, 0QU13JZ, 0QUS3JZ, 0SR007Z, 0SR00JZ, 0SR00KZ, 0SR207Z, 0SR20KZ, 0SR307Z, 0SR30JZ, 0SR30KZ, 0SR407Z, 0SR40KZ, 0SR507Z, 0SR50JZ, 0SR50KZ, 0SR607Z, 0SR60JZ, 0SR60KZ, 0SR707Z, 0SR70JZ, 0SR70KZ, 0SR807Z, 0SR80JZ, 0SR80KZ, 0SU007Z, 0SU00JZ, 0SU00KZ, 0SU037Z, 0SU03JZ, 0SU03KZ, 0SU047Z, 0SU04JZ, 0SU04KZ, 0SU307Z, 0SU30JZ, 0SU30KZ,

0SU337Z, 0SU33JZ, 0SU33KZ, 0SU347Z, 0SU34JZ, 0SU34KZ, 0SU507Z, 0SU50JZ, 0SU50KZ, 0SU537Z, 0SU53JZ, 0SU53KZ, 0SU547Z, 0SU54JZ, 0SU54KZ, 0SU607Z, 0SU60JZ, 0SU60KZ, 0SU637Z, 0SU63JZ, 0SU63KZ, 0SU647Z, 0SU64JZ, 0SU64KZ, 0SU707Z, 0SU70JZ, 0SU70KZ, 0SU737Z, 0SU73JZ, 0SU73KZ, 0SU747Z, 0SU74JZ, 0SU74KZ, 0SU807Z, 0SU80JZ, 0SU80KZ, 0SU837Z, 0SU83JZ, 0SU83KZ, 0SU847Z, 0SU84JZ, 0SU84KZ
**Other tendon repair**<br>0LM30ZZ, 0LM34ZZ, 0LM40ZZ, 0LM44ZZ, 0LM50ZZ, 0LM54ZZ, 0LM60ZZ, 0LM64ZZ, 0LML0ZZ, 0LML4ZZ, 0LMM0ZZ, 0LMM4ZZ, 0LMN0ZZ, 0LMN4ZZ, 0LMP0ZZ, 0LMP4ZZ, 0LQ30ZZ, 0LQ33ZZ, 0LQ34ZZ, 0LQ40ZZ, 0LQ43ZZ, 0LQ44ZZ, 0LQ50ZZ, 0LQ53ZZ, 0LQ54ZZ, 0LQ60ZZ, 0LQ63ZZ, 0LQ64ZZ, 0LQL0ZZ, 0LQL3ZZ, 0LQL4ZZ, 0LQM0ZZ, 0LQM3ZZ, 0LQM4ZZ, 0LQN0ZZ, 0LQN3ZZ, 0LQN4ZZ, 0LQP0ZZ, 0LQP3ZZ, 0LQP4ZZ
**Partial hip replacement**<br>0QR607Z, 0QR60KZ, 0QR637Z, 0QR63KZ, 0QR647Z, 0QR64KZ, 0QR707Z, 0QR70KZ, 0QR737Z, 0QR73KZ, 0QR747Z, 0QR74KZ, 0QR807Z, 0QR80KZ, 0QR837Z, 0QR83KZ, 0QR847Z, 0QR84KZ, 0QR907Z, 0QR90KZ, 0QR937Z, 0QR93KZ, 0QR947Z, 0QR94KZ, 0QRB07Z, 0QRB0KZ, 0QRB37Z, 0QRB3KZ, 0QRB47Z, 0QRB4KZ, 0QRC07Z, 0QRC0KZ, 0QRC37Z, 0QRC3KZ, 0QRC47Z, 0QRC4KZ, 0SRA009, 0SRA00A, 0SRA00Z, 0SRA019, 0SRA01A, 0SRA01Z, 0SRA039, 0SRA03A, 0SRA03Z, 0SRA07Z, 0SRA0J9, 0SRA0JA, 0SRA0JZ, 0SRA0KZ, 0SRE009, 0SRE00A, 0SRE00Z, 0SRE019, 0SRE01A, 0SRE01Z, 0SRE039, 0SRE03A, 0SRE03Z, 0SRE07Z, 0SRE0J9, 0SRE0JA, 0SRE0JZ, 0SRE0KZ, 0SRR019, 0SRR01A, 0SRR01Z, 0SRR039, 0SRR03A, 0SRR03Z, 0SRR07Z, 0SRR0J9, 0SRR0JA, 0SRR0JZ, 0SRR0KZ, 0SRS019, 0SRS01A, 0SRS01Z, 0SRS039, 0SRS03A, 0SRS03Z, 0SRS07Z, 0SRS0J9, 0SRS0JA, 0SRS0JZ, 0SRS0KZ
**Partial knee replacement**<br>0QRD07Z, 0QRD0JZ, 0QRD0KZ, 0QRD37Z, 0QRD3JZ, 0QRD3KZ, 0QRD47Z, 0QRD4JZ, 0QRD4KZ, 0QRF07Z, 0QRF0JZ, 0QRF0KZ, 0QRF37Z, 0QRF3JZ, 0QRF3KZ, 0QRF47Z, 0QRF4JZ, 0QRF4KZ, 0SRC0L9, 0SRC0LA, 0SRC0LZ, 0SRD0L9, 0SRD0LA, 0SRD0LZ, 0SRT07Z, 0SRT0J9, 0SRT0JA, 0SRT0JZ, 0SRT0KZ, 0SRU07Z, 0SRU0J9, 0SRU0JA, 0SRU0JZ, 0SRU0KZ, 0SRV07Z, 0SRV0J9, 0SRV0JA, 0SRV0JZ, 0SRV0KZ, 0SRW07Z, 0SRW0J9, 0SRW0JA, 0SRW0JZ, 0SRW0KZ
**Partial shoulder replacement**<br>0PRC07Z, 0PRC0KZ, 0PRC37Z, 0PRC3KZ, 0PRC47Z, 0PRC4KZ, 0PRD07Z, 0PRD0KZ, 0PRD37Z, 0PRD3KZ, 0PRD47Z, 0PRD4KZ, 0RRJ0J6-0RRJ0J7, 0RRK0J6-0RRK0J7
**Shoulder revision**<br>0PPC04Z, 0PPC05Z, 0PPC07Z, 0PPC0JZ, 0PPC0KZ, 0PPC34Z, 0PPC35Z, 0PPC37Z, 0PPC3JZ, 0PPC3KZ, 0PPC44Z, 0PPC45Z, 0PPC47Z, 0PPC4JZ, 0PPC4KZ, 0PPD04Z, 0PPD05Z, 0PPD07Z, 0PPD0JZ, 0PPD0KZ, 0PPD34Z, 0PPD35Z, 0PPD37Z, 0PPD3JZ, 0PPD3KZ, 0PPD44Z, 0PPD45Z, 0PPD47Z, 0PPD4JZ, 0PPD4KZ, 0RPJ08Z, 0RPJ0JZ, 0RPJ38Z, 0RPJ3JZ, 0RPJ48Z, 0RPJ4JZ, 0RPK08Z, 0RPK0JZ, 0RPK38Z, 0RPK3JZ, 0RPK48Z, 0RPK4JZ, 0RWJ0JZ, 0RWJ3JZ, 0RWJ4JZ, 0RWK0JZ, 0RWK3JZ, 0RWK4JZ
**Spinal cord/spinal meninges lesion excision/destruction**<br>005T0ZZ, 005T3ZZ, 005T4ZZ, 005W0ZZ, 005W3ZZ, 005W4ZZ, 005X0ZZ, 005X3ZZ, 005X4ZZ, 005Y0ZZ, 005Y3ZZ, 005Y4ZZ, 00BT0ZZ, 00BT3ZZ, 00BT4ZZ, 00BW0ZZ, 00BW3ZZ, 00BW4ZZ, 00BX0ZZ, 00BX3ZZ, 00BX4ZZ, 00BY0ZZ, 00BY3ZZ, 00BY4ZZ, 00QT0ZZ, 00QT3ZZ, 00QT4ZZ
**Spinal decompression**<br>00C30ZZ, 00C33ZZ, 00C34ZZ, 00CT0ZZ, 00CT3ZZ, 00CT4ZZ, 00CU0ZZ, 00CU3ZZ, 00CU4ZZ, 00CW0ZZ, 00CW3ZZ, 00CW4ZZ, 00CX0ZZ, 00CX3ZZ, 00CX4ZZ, 00CY0ZZ, 00CY3ZZ, 00CY4ZZ, 00NW0ZZ, 00NW3ZZ, 00NW4ZZ, 00NX0ZZ, 00NX3ZZ, 00NX4ZZ, 00NY0ZZ, 00NY3ZZ, 00NY4ZZ, 0PN30ZZ, 0PN33ZZ, 0PN34ZZ, 0PN40ZZ, 0PN43ZZ, 0PN44ZZ, 0QN00ZZ, 0QN03ZZ, 0QN04ZZ, 0RB00ZZ, 0RB03ZZ, 0RB04ZZ, 0RB10ZZ, 0RB13ZZ, 0RB14ZZ, 0RB30ZZ, 0RB33ZZ, 0RB34ZZ, 0RB40ZZ, 0RB43ZZ, 0RB44ZZ, 0RB50ZZ, 0RB53ZZ, 0RB54ZZ, 0RB60ZZ, 0RB63ZZ, 0RB64ZZ, 0RB90ZZ, 0RB93ZZ, 0RB94ZZ, 0RBA0ZZ, 0RBA3ZZ, 0RBA4ZZ, 0RBB0ZZ, 0RBB3ZZ, 0RBB4ZZ, 0RT30ZZ, 0RT40ZZ, 0RT50ZZ, 0RT90ZZ, 0RTB0ZZ, 0SB00ZZ, 0SB03ZZ, 0SB04ZZ, 0SB20ZZ, 0SB23ZZ, 0SB24ZZ, 0SB30ZZ, 0SB33ZZ, 0SB34ZZ, 0SB40ZZ, 0SB43ZZ, 0SB44ZZ, 0SB50ZZ, 0SB53ZZ, 0SB54ZZ, 0SB60ZZ, 0SB63ZZ, 0SB64ZZ, 0SB70ZZ, 0SB73ZZ, 0SB74ZZ, 0SB80ZZ, 0SB83ZZ, 0SB84ZZ, 0ST20ZZ, 0ST40ZZ

Spinal revision
0PP304Z, 0PP307Z, 0PP30JZ, 0PP30KZ, 0PP334Z, 0PP337Z, 0PP33JZ, 0PP33KZ, 0PP344Z, 0PP347Z, 0PP34JZ, 0PP34KZ, 0PP404Z, 0PP407Z, 0PP40JZ, 0PP40KZ, 0PP434Z, 0PP437Z, 0PP43JZ, 0PP43KZ, 0PP444Z, 0PP447Z, 0PP44JZ, 0PP44KZ, 0QP004Z, 0QP007Z, 0QP00JZ, 0QP00KZ, 0QP034Z, 0QP037Z, 0QP03JZ, 0QP03KZ, 0QP044Z, 0QP047Z, 0QP04JZ, 0QP04KZ, 0QP104Z, 0QP107Z, 0QP10JZ, 0QP10KZ, 0QP134Z, 0QP137Z, 0QP13JZ, 0QP13KZ, 0QP144Z, 0QP147Z, 0QP14JZ, 0QP14KZ, 0QPS04Z, 0QPS07Z, 0QPS0JZ, 0QPS0KZ, 0QPS34Z, 0QPS37Z, 0QPS3JZ, 0QPS3KZ, 0QPS44Z, 0QPS47Z, 0QPS4JZ, 0QPS4KZ, 0RP008Z, 0RP00JZ, 0RP038Z, 0RP03JZ, 0RP048Z, 0RP04JZ, 0RP108Z, 0RP10JZ, 0RP138Z, 0RP13JZ, 0RP148Z, 0RP14JZ, 0RP30JZ, 0RP33JZ, 0RP34JZ, 0RP408Z, 0RP40JZ, 0RP438Z, 0RP43JZ, 0RP448Z, 0RP44JZ, 0RP50JZ, 0RP53JZ, 0RP54JZ, 0RP608Z, 0RP60JZ, 0RP638Z, 0RP63JZ, 0RP648Z, 0RP64JZ, 0RP90JZ, 0RP93JZ, 0RP94JZ, 0RPA08Z, 0RPA0JZ, 0RPA38Z, 0RPA3JZ, 0RPA48Z, 0RPA4JZ, 0RPB0JZ, 0RPB3JZ, 0RPB4JZ, 0SP008Z, 0SP00JZ, 0SP038Z, 0SP03JZ, 0SP048Z, 0SP04JZ, 0SP20JZ, 0SP23JZ, 0SP24JZ, 0SP308Z, 0SP30JZ, 0SP338Z, 0SP33JZ, 0SP348Z, 0SP34JZ, 0SP40JZ, 0SP43JZ, 0SP44JZ, 0SP508Z, 0SP50JZ, 0SP538Z, 0SP53JZ, 0SP548Z, 0SP54JZ, 0SP608Z, 0SP60JZ, 0SP638Z, 0SP63JZ, 0SP648Z, 0SP64JZ, 0SP708Z, 0SP70JZ, 0SP738Z, 0SP73JZ, 0SP748Z, 0SP74JZ, 0SP808Z, 0SP80JZ, 0SP838Z, 0SP83JZ, 0SP848Z, 0SP84JZ

Thoracic/lumbar/sacral fusion
0RG6070-0RG6071, 0RG607J, 0RG60A0-0RG60A1, 0RG60AJ, 0RG60J0-0RG60J1, 0RG60JJ, 0RG60K0-0RG60K1, 0RG60KJ, 0RG60Z0-0RG60Z1, 0RG60ZJ, 0RG6370-0RG6371, 0RG637J, 0RG63A0-0RG63A1, 0RG63AJ, 0RG63J0-0RG63J1, 0RG63JJ, 0RG63K0-0RG63K1, 0RG63KJ, 0RG63Z0-0RG63Z1, 0RG63ZJ, 0RG6470-0RG6471, 0RG647J, 0RG64A0-0RG64A1, 0RG64AJ, 0RG64J0-0RG64J1, 0RG64JJ, 0RG64K0-0RG64K1, 0RG64KJ, 0RG64Z0-0RG64Z1, 0RG64ZJ, 0RG7070-0RG7071, 0RG707J, 0RG70A0-0RG70A1, 0RG70AJ, 0RG70J0-0RG70J1, 0RG70JJ, 0RG70K0-0RG70K1, 0RG70KJ, 0RG70Z0-0RG70Z1, 0RG70ZJ, 0RG7370-0RG7371, 0RG737J, 0RG73A0-0RG73A1, 0RG73AJ, 0RG73J0-0RG73J1, 0RG73JJ, 0RG73K0-0RG73K1, 0RG73KJ, 0RG73Z0-0RG73Z1, 0RG73ZJ, 0RG7470-0RG7471, 0RG747J, 0RG74A0-0RG74A1, 0RG74AJ, 0RG74J0-0RG74J1, 0RG74JJ, 0RG74K0-0RG74K1, 0RG74KJ, 0RG74Z0-0RG74Z1, 0RG74ZJ, 0RG8070-0RG8071, 0RG807J, 0RG80A0-0RG80A1, 0RG80AJ, 0RG80J0-0RG80J1, 0RG80JJ, 0RG80K0-0RG80K1, 0RG80KJ, 0RG80Z0-0RG80Z1, 0RG80ZJ, 0RG8370-0RG8371, 0RG837J, 0RG83A0-0RG83A1, 0RG83AJ, 0RG83J0-0RG83J1, 0RG83JJ, 0RG83K0-0RG83K1, 0RG83KJ, 0RG83Z0-0RG83Z1, 0RG83ZJ, 0RG8470-0RG8471, 0RG847J, 0RG84A0-0RG84A1, 0RG84AJ, 0RG84J0-0RG84J1, 0RG84JJ, 0RG84K0-0RG84K1, 0RG84KJ, 0RG84Z0-0RG84Z1, 0RG84ZJ, 0RGA070-0RGA071, 0RGA07J, 0RGA0A0-0RGA0A1, 0RGA0AJ, 0RGA0J0-0RGA0J1, 0RGA0JJ, 0RGA0K0-0RGA0K1, 0RGA0KJ, 0RGA0Z0-0RGA0Z1, 0RGA0ZJ, 0RGA370-0RGA371, 0RGA37J, 0RGA3A0-0RGA3A1, 0RGA3AJ, 0RGA3J0-0RGA3J1, 0RGA3JJ, 0RGA3K0-0RGA3K1, 0RGA3KJ, 0RGA3Z0-0RGA3Z1, 0RGA3ZJ, 0RGA470-0RGA471, 0RGA47J, 0RGA4A0-0RGA4A1, 0RGA4AJ, 0RGA4J0, 0RGA4J0-0RGA4J1, 0RGA4J1, 0RGA4JJ, 0RGA4K0, 0RGA4K0-0RGA4K1, 0RGA4K1, 0RGA4KJ, 0RGA4Z0, 0RGA4Z0-0RGA4Z1, 0RGA4Z1, 0RGA4ZJ, 0SG0070-0SG0071, 0SG007J, 0SG00A0-0SG00A1, 0SG00AJ, 0SG00J0-0SG00J1, 0SG00JJ, 0SG00K0-0SG00K1, 0SG00KJ, 0SG00Z0-0SG00Z1, 0SG00ZJ, 0SG0370-0SG0371, 0SG037J, 0SG03A0-0SG03A1, 0SG03AJ, 0SG03J0-0SG03J1, 0SG03JJ, 0SG03K0-0SG03K1, 0SG03KJ, 0SG03Z0-0SG03Z1, 0SG03ZJ, 0SG0470-0SG0471, 0SG047J, 0SG04A0-0SG04A1, 0SG04AJ, 0SG04J0-0SG04J1, 0SG04JJ, 0SG04K0-0SG04K1, 0SG04KJ, 0SG04Z0-0SG04Z1, 0SG04ZJ, 0SG1070-0SG1071, 0SG107J, 0SG10A0-0SG10A1, 0SG10AJ, 0SG10J0-0SG10J1, 0SG10JJ, 0SG10K0-0SG10K1, 0SG10KJ, 0SG10Z0-0SG10Z1, 0SG10ZJ, 0SG1370-0SG1371, 0SG137J, 0SG13A0-0SG13A1, 0SG13AJ, 0SG13J0-0SG13J1, 0SG13JJ, 0SG13K0-0SG13K1, 0SG13KJ, 0SG13Z0-0SG13Z1, 0SG13ZJ, 0SG1470-0SG1471, 0SG147J, 0SG14A0-0SG14A1, 0SG14AJ, 0SG14J0-0SG14J1, 0SG14JJ, 0SG14K0-0SG14K1, 0SG14KJ, 0SG14Z0-0SG14Z1, 0SG14ZJ, 0SG3070-0SG3071, 0SG307J, 0SG30A0-0SG30A1, 0SG30AJ, 0SG30J0-0SG30J1, 0SG30JJ, 0SG30K0-0SG30K1, 0SG30KJ, 0SG30Z0-0SG30Z1, 0SG30ZJ, 0SG3370-0SG3371, 0SG337J, 0SG33A0-0SG33A1, 0SG33AJ, 0SG33J0-0SG33J1, 0SG33JJ, 0SG33K0-0SG33K1, 0SG33KJ, 0SG33Z0-0SG33Z1, 0SG33ZJ, 0SG3470-0SG3471, 0SG347J, 0SG34A0-0SG34A1, 0SG34AJ, 0SG34J0-0SG34J1, 0SG34JJ, 0SG34K0-0SG34K1, 0SG34KJ, 0SG34Z0-0SG34Z1, 0SG34ZJ, 0SG504Z, 0SG507Z, 0SG50JZ, 0SG50KZ, 0SG50ZZ, 0SG534Z, 0SG537Z, 0SG53JZ, 0SG53KZ, 0SG53ZZ, 0SG544Z, 0SG547Z, 0SG54JZ, 0SG54KZ, 0SG54ZZ, 0SG704Z, 0SG707Z, 0SG70JZ, 0SG70KZ, 0SG70ZZ, 0SG734Z,

0SG737Z, 0SG73JZ, 0SG73KZ, 0SG73ZZ, 0SG744Z, 0SG747Z, 0SG74JZ, 0SG74KZ, 0SG74ZZ, 0SG804Z, 0SG807Z, 0SG80JZ, 0SG80KZ, 0SG80ZZ, 0SG834Z, 0SG837Z, 0SG83JZ, 0SG83KZ, 0SG83ZZ, 0SG844Z, 0SG847Z, 0SG84JZ, 0SG84KZ, 0SG84ZZ, XRG6092, XRG60F3, XRG7092, XRG70F3, XRG8092, XRG80F3, XRGA092, XRGA0F3, XRGB092, XRGB0F3, XRGC092, XRGC0F3, XRGD092, XRGD0F3
**Tibia/Fibula repair**<br>0QHG04Z, 0QHG05Z, 0QHG06Z, 0QHG0BZ, 0QHG0CZ, 0QHG0DZ, 0QHG34Z, 0QHG35Z, 0QHG36Z, 0QHG3BZ, 0QHG3CZ, 0QHG3DZ, 0QHG44Z, 0QHG45Z, 0QHG46Z, 0QHG4BZ, 0QHG4CZ, 0QHG4DZ, 0QHH04Z, 0QHH05Z, 0QHH06Z, 0QHH0BZ, 0QHH0CZ, 0QHH0DZ, 0QHH34Z, 0QHH35Z, 0QHH36Z, 0QHH3BZ, 0QHH3CZ, 0QHH3DZ, 0QHH44Z, 0QHH45Z, 0QHH46Z, 0QHH4BZ, 0QHH4CZ, 0QHH4DZ, 0QHJ04Z, 0QHJ05Z, 0QHJ06Z, 0QHJ0BZ, 0QHJ0CZ, 0QHJ0DZ, 0QHJ34Z, 0QHJ35Z, 0QHJ36Z, 0QHJ3BZ, 0QHJ3CZ, 0QHJ3DZ, 0QHJ44Z, 0QHJ45Z, 0QHJ46Z, 0QHJ4BZ, 0QHJ4CZ, 0QHJ4DZ, 0QHK04Z, 0QHK05Z, 0QHK06Z, 0QHK0BZ, 0QHK0CZ, 0QHK0DZ, 0QHK34Z, 0QHK35Z, 0QHK36Z, 0QHK3BZ, 0QHK3CZ, 0QHK3DZ, 0QHK44Z, 0QHK45Z, 0QHK46Z, 0QHK4BZ, 0QHK4CZ, 0QHK4DZ, 0QSG04Z, 0QSG05Z, 0QSG06Z, 0QSG0BZ, 0QSG0CZ, 0QSG0DZ, 0QSG0ZZ, 0QSG34Z, 0QSG35Z, 0QSG3BZ, 0QSG3CZ, 0QSG3DZ, 0QSG44Z, 0QSG45Z, 0QSG4BZ, 0QSG4CZ, 0QSG4DZ, 0QSH04Z, 0QSH05Z, 0QSH06Z, 0QSH0BZ, 0QSH0CZ, 0QSH0DZ, 0QSH0ZZ, 0QSH34Z, 0QSH35Z, 0QSH3BZ, 0QSH3CZ, 0QSH3DZ, 0QSH44Z, 0QSH45Z, 0QSH4BZ, 0QSH4CZ, 0QSH4DZ, 0QSJ04Z, 0QSJ05Z, 0QSJ06Z, 0QSJ0BZ, 0QSJ0CZ, 0QSJ0DZ, 0QSJ0ZZ, 0QSJ34Z, 0QSJ35Z, 0QSJ3BZ, 0QSJ3CZ, 0QSJ3DZ, 0QSJ44Z, 0QSJ45Z, 0QSJ4BZ, 0QSJ4CZ, 0QSJ4DZ, 0QSK04Z, 0QSK05Z, 0QSK06Z, 0QSK0BZ, 0QSK0CZ, 0QSK0DZ, 0QSK0ZZ, 0QSK34Z, 0QSK35Z, 0QSK3BZ, 0QSK3CZ, 0QSK3DZ, 0QSK44Z, 0QSK45Z, 0QSK4BZ, 0QSK4CZ, 0QSK4DZ, 0QUG07Z, 0QUG0KZ, 0QUG37Z, 0QUG3KZ, 0QUG47Z, 0QUG4KZ, 0QUH07Z, 0QUH0KZ, 0QUH37Z, 0QUH3KZ, 0QUH47Z, 0QUH4KZ, 0QUJ07Z, 0QUJ0KZ, 0QUJ37Z, 0QUJ3KZ, 0QUJ47Z, 0QUJ4KZ, 0QUK07Z, 0QUK0KZ, 0QUK37Z, 0QUK3KZ, 0QUK47Z, 0QUK4KZ
**Tibia/Fibula revision**<br>0QPG04Z, 0QPG05Z, 0QPG07Z, 0QPG0JZ, 0QPG0KZ, 0QPG34Z, 0QPG35Z, 0QPG37Z, 0QPG3JZ, 0QPG3KZ, 0QPG44Z, 0QPG45Z, 0QPG47Z, 0QPG4JZ, 0QPG4KZ, 0QPH04Z, 0QPH05Z, 0QPH07Z, 0QPH0JZ, 0QPH0KZ, 0QPH34Z, 0QPH35Z, 0QPH37Z, 0QPH3JZ, 0QPH3KZ, 0QPH44Z, 0QPH45Z, 0QPH47Z, 0QPH4JZ, 0QPH4KZ, 0QPJ04Z, 0QPJ05Z, 0QPJ07Z, 0QPJ0JZ, 0QPJ0KZ, 0QPJ34Z, 0QPJ35Z, 0QPJ37Z, 0QPJ3JZ, 0QPJ3KZ, 0QPJ44Z, 0QPJ45Z, 0QPJ47Z, 0QPJ4JZ, 0QPJ4KZ, 0QPK04Z, 0QPK05Z, 0QPK07Z, 0QPK0JZ, 0QPK0KZ, 0QPK34Z, 0QPK35Z, 0QPK37Z, 0QPK3JZ, 0QPK3KZ, 0QPK44Z, 0QPK45Z, 0QPK47Z, 0QPK4JZ, 0QPK4KZ
**Total hip replacement**<br>0SR9019, 0SR901A, 0SR901Z, 0SR9029, 0SR902A, 0SR902Z, 0SR9039, 0SR903A, 0SR903Z, 0SR9049, 0SR904A, 0SR904Z, 0SR9069, 0SR906A, 0SR906Z, 0SR907Z, 0SR90EZ, 0SR90J9, 0SR90JA, 0SR90JZ, 0SR90KZ, 0SRB019, 0SRB01A, 0SRB01Z, 0SRB029, 0SRB02A, 0SRB02Z, 0SRB039, 0SRB03A, 0SRB03Z, 0SRB049, 0SRB04A, 0SRB04Z, 0SRB069, 0SRB06A, 0SRB06Z, 0SRB07Z, 0SRB0EZ, 0SRB0J9, 0SRB0JA, 0SRB0JZ, 0SRB0KZ
**Total knee replacement**<br>0SRC069, 0SRC06A, 0SRC06Z, 0SRC07Z, 0SRC0EZ, 0SRC0J9, 0SRC0JA, 0SRC0JZ, 0SRC0KZ, 0SRD069, 0SRD06A, 0SRD06Z, 0SRD07Z, 0SRD0EZ, 0SRD0J9, 0SRD0JA, 0SRD0JZ, 0SRD0KZ
**Total shoulder replacement**<br>0RRJ07Z, 0RRJ0JZ, 0RRJ0KZ, 0RRK07Z, 0RRK0JZ, 0RRK0KZ
**Vertebral fracture repair**<br>0PS304Z, 0PS30ZZ, 0PS334Z, 0PS33ZZ, 0PS344Z, 0PS34ZZ, 0PS3XZZ, 0PS404Z, 0PS40ZZ, 0PS434Z, 0PS43ZZ, 0PS444Z, 0PS44ZZ, 0PS4XZZ, 0QS004Z, 0QS00ZZ, 0QS034Z, 0QS03ZZ, 0QS044Z, 0QS04ZZ, 0QS0XZZ, 0QS104Z, 0QS10ZZ, 0QS134Z, 0QS13ZZ, 0QS144Z, 0QS14ZZ, 0QS1XZZ, 0QSS04Z, 0QSS0ZZ, 0QSS34Z, 0QSS3ZZ, 0QSS44Z, 0QSS4ZZ, 0QSSXZZ

**Supplemental Digital Content Table 3. List of ICD-10 principal procedure codes for Vascular Surgery, categorized into clinically relevant procedure groups**
**Carotid artery stent**<br>037H046, 037H04Z, 037H056, 037H05Z, 037H066, 037H06Z, 037H076, 037H07Z, 037H0D6, 037H0DZ, 037H0E6, 037H0EZ, 037H0F6, 037H0FZ, 037H0G6, 037H0GZ, 037H0Z6, 037H0ZZ, 037H346, 037H34Z, 037H356, 037H35Z, 037H366, 037H36Z, 037H376, 037H37Z, 037H3D6, 037H3DZ, 037H3E6, 037H3EZ, 037H3F6, 037H3FZ, 037H3G6, 037H3GZ, 037H3Z6, 037H3ZZ, 037H446, 037H44Z, 037H456, 037H45Z, 037H466, 037H46Z, 037H476, 037H47Z, 037H4D6, 037H4DZ, 037H4E6, 037H4EZ, 037H4F6, 037H4FZ, 037H4G6, 037H4GZ, 037H4Z6, 037H4ZZ, 037J046, 037J04Z, 037J056, 037J05Z, 037J066, 037J06Z, 037J076, 037J07Z, 037J0D6, 037J0DZ, 037J0E6, 037J0EZ, 037J0F6, 037J0FZ, 037J0G6, 037J0GZ, 037J0Z6, 037J0ZZ, 037J346, 037J34Z, 037J356, 037J35Z, 037J366, 037J36Z, 037J376, 037J37Z, 037J3D6, 037J3DZ, 037J3E6, 037J3EZ, 037J3F6, 037J3FZ, 037J3G6, 037J3GZ, 037J3Z6, 037J3ZZ, 037J446, 037J44Z, 037J456, 037J45Z, 037J466, 037J46Z, 037J476, 037J47Z, 037J4D6, 037J4DZ, 037J4E6, 037J4EZ, 037J4F6, 037J4FZ, 037J4G6, 037J4GZ, 037J4Z6, 037J4ZZ, 037K046, 037K04Z, 037K056, 037K05Z, 037K066, 037K06Z, 037K076, 037K07Z, 037K0D6, 037K0DZ, 037K0E6, 037K0EZ, 037K0F6, 037K0FZ, 037K0G6, 037K0GZ, 037K0Z6, 037K0ZZ, 037K346, 037K34Z, 037K356, 037K35Z, 037K366, 037K36Z, 037K376, 037K37Z, 037K3D6, 037K3DZ, 037K3E6, 037K3EZ, 037K3F6, 037K3FZ, 037K3G6, 037K3GZ, 037K3Z6, 037K3ZZ, 037K446, 037K44Z, 037K456, 037K45Z, 037K466, 037K46Z, 037K476, 037K47Z, 037K4D6, 037K4DZ, 037K4E6, 037K4EZ, 037K4F6, 037K4FZ, 037K4G6, 037K4GZ, 037K4Z6, 037K4ZZ, 037L046, 037L04Z, 037L056, 037L05Z, 037L066, 037L06Z, 037L076, 037L07Z, 037L0D6, 037L0DZ, 037L0E6, 037L0EZ, 037L0F6, 037L0FZ, 037L0G6, 037L0GZ, 037L0Z6, 037L0ZZ, 037L346, 037L34Z, 037L356, 037L35Z, 037L366, 037L36Z, 037L376, 037L37Z, 037L3D6, 037L3DZ, 037L3E6, 037L3EZ, 037L3F6, 037L3FZ, 037L3G6, 037L3GZ, 037L3Z6, 037L3ZZ, 037L446, 037L44Z, 037L456, 037L45Z, 037L466, 037L46Z, 037L476, 037L47Z, 037L4D6, 037L4DZ, 037L4E6, 037L4EZ, 037L4F6, 037L4FZ, 037L4G6, 037L4GZ, 037L4Z6, 037L4ZZ, 037M046, 037M04Z, 037M056, 037M05Z, 037M066, 037M06Z, 037M076, 037M07Z, 037M0D6, 037M0DZ, 037M0E6, 037M0EZ, 037M0F6, 037M0FZ, 037M0G6, 037M0GZ, 037M0Z6, 037M0ZZ, 037M346, 037M34Z, 037M356, 037M35Z, 037M366, 037M36Z, 037M376, 037M37Z, 037M3D6, 037M3DZ, 037M3E6, 037M3EZ, 037M3F6, 037M3FZ, 037M3G6, 037M3GZ, 037M3Z6, 037M3ZZ, 037M446, 037M44Z, 037M456, 037M45Z, 037M466, 037M46Z, 037M476, 037M47Z, 037M4D6, 037M4DZ, 037M4E6, 037M4EZ, 037M4F6, 037M4FZ, 037M4G6, 037M4GZ, 037M4Z6, 037M4ZZ, 037N046, 037N04Z, 037N056, 037N05Z, 037N066, 037N06Z, 037N076, 037N07Z, 037N0D6, 037N0DZ, 037N0E6, 037N0EZ, 037N0F6, 037N0FZ, 037N0G6, 037N0GZ, 037N0Z6, 037N0ZZ, 037N346, 037N34Z, 037N356, 037N35Z, 037N366, 037N36Z, 037N376, 037N37Z, 037N3D6, 037N3DZ, 037N3E6, 037N3EZ, 037N3F6, 037N3FZ, 037N3G6, 037N3GZ, 037N3Z6, 037N3ZZ, 037N446, 037N44Z, 037N456, 037N45Z, 037N466, 037N46Z, 037N476, 037N47Z, 037N4D6, 037N4DZ, 037N4E6, 037N4EZ, 037N4F6, 037N4FZ, 037N4G6, 037N4GZ, 037N4Z6, 037N4ZZ, 03VH3DZ, 03VH3ZZ, 03VH4DZ, 03VJ3DZ, 03VJ3ZZ, 03VJ4DZ, 03VK3CZ, 03VK3DZ, 03VK3ZZ, 03VK4CZ, 03VK4DZ, 03VL3CZ, 03VL3DZ, 03VL3ZZ, 03VL4CZ, 03VL4DZ, 03VM3DZ, 03VM3ZZ, 03VM4DZ, 03VN3DZ, 03VN3ZZ, 03VN4DZ
**Carotid endarterectomy**<br>03CH0Z6, 03CH0ZZ, 03CH3Z6, 03CH3ZZ, 03CH4Z6, 03CH4ZZ, 03CJ0Z6, 03CJ0ZZ, 03CJ3Z6, 03CJ3ZZ, 03CJ4Z6, 03CJ4ZZ, 03CK0Z6, 03CK0ZZ, 03CK3Z6, 03CK3ZZ, 03CK4Z6, 03CK4ZZ, 03CL0Z6, 03CL0ZZ, 03CL3Z6, 03CL3ZZ, 03CL4Z6, 03CL4ZZ, 03CM0Z6, 03CM0ZZ, 03CM3Z6, 03CM3ZZ, 03CM4Z6, 03CM4ZZ, 03CN0Z6, 03CN0ZZ, 03CN3Z6, 03CN3ZZ, 03CN4Z6, 03CN4ZZ
**Dialysis Access**<br>031209D, 031209F, 03120AD, 03120AF, 03120JD, 03120JF, 03120KD, 03120KF, 03120ZD, 03120ZF, 031309D, 031309F, 03130AD, 03130AF, 03130JD, 03130JF, 03130KD, 03130KF, 03130ZD, 03130ZF, 031409D, 031409F, 03140AD, 03140AF, 03140JD, 03140JF, 03140KD, 03140KF, 03140ZD, 03140ZF, 031509D, 031509F, 03150AD, 03150AF, 03150JD, 03150JF, 03150KD, 03150KF, 03150ZD, 03150ZF, 031609D, 031609F, 03160AD, 03160AF, 03160JD, 03160JF, 03160KD, 03160KF, 03160ZD, 03160ZF, 031709D, 031709F, 03170AD, 03170AF, 03170JD, 03170JF, 03170KD, 03170KF, 03170ZD, 03170ZF, 031809D, 031809F, 03180AD, 03180AF, 03180JD, 03180JF, 03180KD, 03180KF, 03180ZD, 03180ZF, 031909F, 03190AF, 03190JF, 03190KF, 03190ZF, 031A09F, 031A0AF, 031A0JF, 031A0KF, 031A0ZF,

031B09F, 031B0AF, 031B0JF, 031B0KF, 031B0ZF, 031C09F, 031C0AF, 031C0JF, 031C0KF, 031C0ZF, 03LY3DZ, 03LY3ZZ, 03LY4DZ, 03PY07Z, 03PY0CZ, 03PY0DZ, 03PY0JZ, 03PY0KZ, 03WY0JZ, 03WY3JZ, 03WY4JZ
**Endo abdominal artery repair**<br>04V13DZ, 04V13ZZ, 04V14DZ, 04V23DZ, 04V23ZZ, 04V24DZ, 04V33DZ, 04V33ZZ, 04V34DZ, 04V43DZ, 04V43ZZ, 04V44DZ, 04V53DZ, 04V53ZZ, 04V54DZ, 04V63DZ, 04V63ZZ, 04V64DZ, 04V73DZ, 04V73ZZ, 04V74DZ, 04V83DZ, 04V83ZZ, 04V84DZ, 04V93DZ, 04V93ZZ, 04V94DZ, 04VA3DZ, 04VA3ZZ, 04VA4DZ, 04VB3DZ, 04VB3ZZ, 04VB4DZ, 04VC3DZ, 04VC3EZ, 04VC3FZ, 04VC3ZZ, 04VC4DZ, 04VC4EZ, 04VC4FZ, 04VD3DZ, 04VD3EZ, 04VD3FZ, 04VD3ZZ, 04VD4DZ, 04VD4EZ, 04VD4FZ, 04VE3DZ, 04VE3ZZ, 04VE4DZ, 04VF3DZ, 04VF3ZZ, 04VF4DZ, 04VH3DZ, 04VH3ZZ, 04VH4DZ, 04VJ3DZ, 04VJ3ZZ, 04VJ4DZ
**Endo abdominal artery revascularization**<br>0470341, 0470346, 047034Z, 0470356, 047035Z, 0470366, 047036Z, 0470376, 047037Z, 04703D1, 04703D6, 04703DZ, 04703E6, 04703EZ, 04703F6, 04703FZ, 04703G6, 04703GZ, 04703Z1, 04703Z6, 04703ZZ, 0470441, 0470446, 047044Z, 0470456, 047045Z, 0470466, 047046Z, 0470476, 047047Z, 04704D1, 04704D6, 04704DZ, 04704E6, 04704EZ, 04704F6, 04704FZ, 04704G6, 04704GZ, 04704Z1, 04704Z6, 04704ZZ, 0471341, 0471346, 047134Z, 0471356, 047135Z, 0471366, 047136Z, 0471376, 047137Z, 04713D1, 04713D6, 04713DZ, 04713E6, 04713EZ, 04713F6, 04713FZ, 04713G6, 04713GZ, 04713Z1, 04713Z6, 04713ZZ, 0471441, 0471446, 047144Z, 0471456, 047145Z, 0471466, 047146Z, 0471476, 047147Z, 04714D1, 04714D6, 04714DZ, 04714E6, 04714EZ, 04714F6, 04714FZ, 04714G6, 04714GZ, 04714Z1, 04714Z6, 04714ZZ, 0472341, 0472346, 047234Z, 0472356, 047235Z, 0472366, 047236Z, 0472376, 047237Z, 04723D1, 04723D6, 04723DZ, 04723E6, 04723EZ, 04723F6, 04723FZ, 04723G6, 04723GZ, 04723Z1, 04723Z6, 04723ZZ, 0472441, 0472446, 047244Z, 0472456, 047245Z, 0472466, 047246Z, 0472476, 047247Z, 04724D1, 04724D6, 04724DZ, 04724E6, 04724EZ, 04724F6, 04724FZ, 04724G6, 04724GZ, 04724Z1, 04724Z6, 04724ZZ, 0473341, 0473346, 047334Z, 0473356, 047335Z, 0473366, 047336Z, 0473376, 047337Z, 04733D1, 04733D6, 04733DZ, 04733E6, 04733EZ, 04733F6, 04733FZ, 04733G6, 04733GZ, 04733Z1, 04733Z6, 04733ZZ, 0473441, 0473446, 047344Z, 0473456, 047345Z, 0473466, 047346Z, 0473476, 047347Z, 04734D1, 04734D6, 04734DZ, 04734E6, 04734EZ, 04734F6, 04734FZ, 04734G6, 04734GZ, 04734Z1, 04734Z6, 04734ZZ, 0474341, 0474346, 047434Z, 0474356, 047435Z, 0474366, 047436Z, 0474376, 047437Z, 04743D1, 04743D6, 04743DZ, 04743E6, 04743EZ, 04743F6, 04743FZ, 04743G6, 04743GZ, 04743Z1, 04743Z6, 04743ZZ, 0474441, 0474446, 047444Z, 0474456, 047445Z, 0474466, 047446Z, 0474476, 047447Z, 04744D1, 04744D6, 04744DZ, 04744E6, 04744EZ, 04744F6, 04744FZ, 04744G6, 04744GZ, 04744Z1, 04744Z6, 04744ZZ, 0475341, 0475346, 047534Z, 0475356, 047535Z, 0475366, 047536Z, 0475376, 047537Z, 04753D1, 04753D6, 04753DZ, 04753E6, 04753EZ, 04753F6, 04753FZ, 04753G6, 04753GZ, 04753Z1, 04753Z6, 04753ZZ, 0475441, 0475446, 047544Z, 0475456, 047545Z, 0475466, 047546Z, 0475476, 047547Z, 04754D1, 04754D6, 04754DZ, 04754E6, 04754EZ, 04754F6, 04754FZ, 04754G6, 04754GZ, 04754Z1, 04754Z6, 04754ZZ, 0476341, 0476346, 047634Z, 0476356, 047635Z, 0476366, 047636Z, 0476376, 047637Z, 04763D1, 04763D6, 04763DZ, 04763E6, 04763EZ, 04763F6, 04763FZ, 04763G6, 04763GZ, 04763Z1, 04763Z6, 04763ZZ, 0476441, 0476446, 047644Z, 0476456, 047645Z, 0476466, 047646Z, 0476476, 047647Z, 04764D1, 04764D6, 04764DZ, 04764E6, 04764EZ, 04764F6, 04764FZ, 04764G6, 04764GZ, 04764Z1, 04764Z6, 04764ZZ, 0477341, 0477346, 047734Z, 0477356, 047735Z, 0477366, 047736Z, 0477376, 047737Z, 04773D1, 04773D6, 04773DZ, 04773E6, 04773EZ, 04773F6, 04773FZ, 04773G6, 04773GZ, 04773Z1, 04773Z6, 04773ZZ, 0477441, 0477446, 047744Z, 0477456, 047745Z, 0477466, 047746Z, 0477476, 047747Z, 04774D1, 04774D6, 04774DZ, 04774E6, 04774EZ, 04774F6, 04774FZ, 04774G6, 04774GZ, 04774Z1, 04774Z6, 04774ZZ, 0478341, 0478346, 047834Z, 0478356, 047835Z, 0478366, 047836Z, 0478376, 047837Z, 04783D1, 04783D6, 04783DZ, 04783E6, 04783EZ, 04783F6, 04783FZ, 04783G6, 04783GZ, 04783Z1, 04783Z6, 04783ZZ, 0478441, 0478446, 047844Z, 0478456, 047845Z, 0478466, 047846Z, 0478476, 047847Z, 04784D1, 04784D6, 04784DZ, 04784E6, 04784EZ, 04784F6, 04784FZ, 04784G6, 04784GZ, 04784Z1, 04784Z6, 04784ZZ, 0479341, 0479346, 047934Z, 0479356, 047935Z, 0479366, 047936Z, 0479376, 047937Z, 04793D1, 04793D6, 04793DZ, 04793E6, 04793EZ, 04793F6, 04793FZ, 04793G6, 04793GZ, 04793Z1, 04793Z6, 04793ZZ, 0479441, 0479446, 047944Z, 0479456, 047945Z, 0479466, 047946Z, 0479476, 047947Z, 04794D1, 04794D6, 04794DZ, 04794E6, 04794EZ, 04794F6, 04794FZ, 04794G6, 04794GZ, 04794Z1, 04794Z6, 04794ZZ, 047A341, 047A346, 047A34Z, 047A356, 047A35Z, 047A366, 047A36Z, 047A376, 047A37Z, 047A3D1, 047A3D6, 047A3DZ, 047A3E6, 047A3EZ, 047A3F6, 047A3FZ,

047A3G6, 047A3GZ, 047A3Z1, 047A3Z6, 047A3ZZ, 047A441, 047A446, 047A44Z, 047A456, 047A45Z, 047A466, 047A46Z, 047A476, 047A47Z, 047A4D1, 047A4D6, 047A4DZ, 047A4E6, 047A4EZ, 047A4F6, 047A4FZ, 047A4G6, 047A4GZ, 047A4Z1, 047A4Z6, 047A4ZZ, 047B341, 047B346, 047B34Z, 047B356, 047B35Z, 047B366, 047B36Z, 047B376, 047B37Z, 047B3D1, 047B3D6, 047B3DZ, 047B3E6, 047B3EZ, 047B3F6, 047B3FZ, 047B3G6, 047B3GZ, 047B3Z1, 047B3Z6, 047B3ZZ, 047B441, 047B446, 047B44Z, 047B456, 047B45Z, 047B466, 047B46Z, 047B476, 047B47Z, 047B4D1, 047B4D6, 047B4DZ, 047B4E6, 047B4EZ, 047B4F6, 047B4FZ, 047B4G6, 047B4GZ, 047B4Z1, 047B4Z6, 047B4ZZ, 047C341, 047C346, 047C34Z, 047C356, 047C35Z, 047C366, 047C36Z, 047C376, 047C37Z, 047C3D1, 047C3D6, 047C3DZ, 047C3E6, 047C3EZ, 047C3F6, 047C3FZ, 047C3G6, 047C3GZ, 047C3Z1, 047C3Z6, 047C3ZZ, 047C441, 047C446, 047C44Z, 047C456, 047C45Z, 047C466, 047C46Z, 047C476, 047C47Z, 047C4D1, 047C4D6, 047C4DZ, 047C4E6, 047C4EZ, 047C4F6, 047C4FZ, 047C4G6, 047C4GZ, 047C4Z1, 047C4Z6, 047C4ZZ, 047D341, 047D346, 047D34Z, 047D356, 047D35Z, 047D366, 047D36Z, 047D376, 047D37Z, 047D3D1, 047D3D6, 047D3DZ, 047D3E6, 047D3EZ, 047D3F6, 047D3FZ, 047D3G6, 047D3GZ, 047D3Z1, 047D3Z6, 047D3ZZ, 047D441, 047D446, 047D44Z, 047D456, 047D45Z, 047D466, 047D46Z, 047D476, 047D47Z, 047D4D1, 047D4D6, 047D4DZ, 047D4E6, 047D4EZ, 047D4F6, 047D4FZ, 047D4G6, 047D4GZ, 047D4Z1, 047D4Z6, 047D4ZZ, 047E341, 047E346, 047E34Z, 047E356, 047E35Z, 047E366, 047E36Z, 047E376, 047E37Z, 047E3D1, 047E3D6, 047E3DZ, 047E3E6, 047E3EZ, 047E3F6, 047E3FZ, 047E3G6, 047E3GZ, 047E3Z1, 047E3Z6, 047E3ZZ, 047E441, 047E446, 047E44Z, 047E456, 047E45Z, 047E466, 047E46Z, 047E476, 047E47Z, 047E4D1, 047E4D6, 047E4DZ, 047E4E6, 047E4EZ, 047E4F6, 047E4FZ, 047E4G6, 047E4GZ, 047E4Z1, 047E4Z6, 047E4ZZ, 047F341, 047F346, 047F34Z, 047F356, 047F35Z, 047F366, 047F36Z, 047F376, 047F37Z, 047F3D1, 047F3D6, 047F3DZ, 047F3E6, 047F3EZ, 047F3F6, 047F3FZ, 047F3G6, 047F3GZ, 047F3Z1, 047F3Z6, 047F3ZZ, 047F441, 047F446, 047F44Z, 047F456, 047F45Z, 047F466, 047F46Z, 047F476, 047F47Z, 047F4D1, 047F4D6, 047F4DZ, 047F4E6, 047F4EZ, 047F4F6, 047F4FZ, 047F4G6, 047F4GZ, 047F4Z1, 047F4Z6, 047F4ZZ, 047H341, 047H346, 047H34Z, 047H356, 047H35Z, 047H366, 047H36Z, 047H376, 047H37Z, 047H3D1, 047H3D6, 047H3DZ, 047H3E6, 047H3EZ, 047H3F6, 047H3FZ, 047H3G6, 047H3GZ, 047H3Z1, 047H3Z6, 047H3ZZ, 047H441, 047H446, 047H44Z, 047H456, 047H45Z, 047H466, 047H46Z, 047H476, 047H47Z, 047H4D1, 047H4D6, 047H4DZ, 047H4E6, 047H4EZ, 047H4F6, 047H4FZ, 047H4G6, 047H4GZ, 047H4Z1, 047H4Z6, 047H4ZZ, 047J341, 047J346, 047J34Z, 047J356, 047J35Z, 047J366, 047J36Z, 047J376, 047J37Z, 047J3D1, 047J3D6, 047J3DZ, 047J3E6, 047J3EZ, 047J3F6, 047J3FZ, 047J3G6, 047J3GZ, 047J3Z1, 047J3Z6, 047J3ZZ, 047J441, 047J446, 047J44Z, 047J456, 047J45Z, 047J466, 047J46Z, 047J476, 047J47Z, 047J4D1, 047J4D6, 047J4DZ, 047J4E6, 047J4EZ, 047J4F6, 047J4FZ, 047J4G6, 047J4GZ, 047J4Z1, 047J4Z6, 047J4ZZ, 04C13Z6, 04C13ZZ, 04C14Z6, 04C14ZZ, 04C23Z6, 04C23ZZ, 04C24Z6, 04C24ZZ, 04C33Z6, 04C33ZZ, 04C34Z6, 04C34ZZ, 04C43Z6, 04C43ZZ, 04C44Z6, 04C44ZZ, 04C53Z6, 04C53ZZ, 04C54Z6, 04C54ZZ, 04C63Z6, 04C63ZZ, 04C64Z6, 04C64ZZ, 04C73Z6, 04C73ZZ, 04C74Z6, 04C74ZZ, 04C83Z6, 04C83ZZ, 04C84Z6, 04C84ZZ, 04C93Z6, 04C93ZZ, 04C94Z6, 04C94ZZ, 04CA3Z6, 04CA3ZZ, 04CA4Z6, 04CA4ZZ, 04CB3Z6, 04CB3ZZ, 04CB4Z6, 04CB4ZZ, 04R147Z, 04R14JZ, 04R14KZ, 04R247Z, 04R24JZ, 04R24KZ, 04R347Z, 04R34JZ, 04R34KZ, 04R447Z, 04R44JZ, 04R44KZ, 04R547Z, 04R54JZ, 04R54KZ, 04R647Z, 04R64JZ, 04R64KZ, 04R747Z, 04R74JZ, 04R74KZ, 04R847Z, 04R84JZ, 04R84KZ, 04R947Z, 04R94JZ, 04R94KZ, 04RA47Z, 04RA4JZ, 04RA4KZ, 04RB47Z, 04RB4JZ, 04RB4KZ, 04RC47Z, 04RC4JZ, 04RC4KZ, 04RD47Z, 04RD4JZ, 04RD4KZ, 04RE47Z, 04RE4JZ, 04RE4KZ, 04RF47Z, 04RF4JZ, 04RF4KZ, 04RH47Z, 04RH4JZ, 04RH4KZ, 04RJ47Z, 04RJ4JZ, 04RJ4KZ, 04U137Z, 04U13JZ, 04U13KZ, 04U147Z, 04U14JZ, 04U14KZ, 04U237Z, 04U23JZ, 04U23KZ, 04U247Z, 04U24JZ, 04U24KZ, 04U337Z, 04U33JZ, 04U33KZ, 04U347Z, 04U34JZ, 04U34KZ, 04U437Z, 04U43JZ, 04U43KZ, 04U447Z, 04U44JZ, 04U44KZ, 04U537Z, 04U53JZ, 04U53KZ, 04U547Z, 04U54JZ, 04U54KZ, 04U637Z, 04U63JZ, 04U63KZ, 04U647Z, 04U64JZ, 04U64KZ, 04U737Z, 04U73JZ, 04U73KZ, 04U747Z, 04U74JZ, 04U74KZ, 04U837Z, 04U83JZ, 04U83KZ, 04U847Z, 04U84JZ, 04U84KZ, 04U937Z, 04U93JZ, 04U93KZ, 04U947Z, 04U94JZ, 04U94KZ, 04UA37Z, 04UA3JZ, 04UA3KZ, 04UA47Z, 04UA4JZ, 04UA4KZ, 04UB37Z, 04UB3JZ, 04UB3KZ, 04UB47Z, 04UB4JZ, 04UB4KZ, 04UC37Z, 04UC3JZ, 04UC3KZ, 04UC47Z, 04UC4JZ, 04UC4KZ, 04UD37Z, 04UD3JZ, 04UD3KZ, 04UD47Z, 04UD4JZ, 04UD4KZ, 04UE37Z, 04UE3JZ, 04UE3KZ, 04UE47Z, 04UE4JZ, 04UE4KZ, 04UF37Z, 04UF3JZ, 04UF3KZ, 04UF47Z,

04UF4JZ, 04UF4KZ, 04UH37Z, 04UH3JZ, 04UH3KZ, 04UH47Z, 04UH4JZ, 04UH4KZ, 04UJ37Z, 04UJ3JZ, 04UJ3KZ, 04UJ47Z, 04UJ4JZ, 04UJ4KZ
**Endo aortic/iliac revascularization**<br>04C03Z6, 04C03ZZ, 04C04Z6, 04C04ZZ, 04CC3Z6, 04CC3ZZ, 04CC4Z6, 04CC4ZZ, 04CD3Z6, 04CD3ZZ, 04CD4Z6, 04CD4ZZ, 04CE3Z6, 04CE3ZZ, 04CE4Z6, 04CE4ZZ, 04CF3Z6, 04CF3ZZ, 04CF4Z6, 04CF4ZZ, 04CH3Z6, 04CH3ZZ, 04CH4Z6, 04CH4ZZ, 04CJ3Z6, 04CJ3ZZ, 04CJ4Z6, 04CJ4ZZ
**Endo upper extremity revascularization**<br>0373346, 037334Z, 0373356, 037335Z, 0373366, 037336Z, 0373376, 037337Z, 03733D6, 03733DZ, 03733E6, 03733EZ, 03733F6, 03733FZ, 03733G6, 03733GZ, 03733Z6, 03733ZZ, 0373446, 037344Z, 0373456, 037345Z, 0373466, 037346Z, 0373476, 037347Z, 03734D6, 03734DZ, 03734E6, 03734EZ, 03734F6, 03734FZ, 03734G6, 03734GZ, 03734Z6, 03734ZZ, 0374346, 037434Z, 0374356, 037435Z, 0374366, 037436Z, 0374376, 037437Z, 03743D6, 03743DZ, 03743E6, 03743EZ, 03743F6, 03743FZ, 03743G6, 03743GZ, 03743Z6, 03743ZZ, 0374446, 037444Z, 0374456, 037445Z, 0374466, 037446Z, 0374476, 037447Z, 03744D6, 03744DZ, 03744E6, 03744EZ, 03744F6, 03744FZ, 03744G6, 03744GZ, 03744Z6, 03744ZZ, 0375346, 037534Z, 0375356, 037535Z, 0375366, 037536Z, 0375376, 037537Z, 03753D6, 03753DZ, 03753E6, 03753EZ, 03753F6, 03753FZ, 03753G6, 03753GZ, 03753Z6, 03753ZZ, 0375446, 037544Z, 0375456, 037545Z, 0375466, 037546Z, 0375476, 037547Z, 03754D6, 03754DZ, 03754E6, 03754EZ, 03754F6, 03754FZ, 03754G6, 03754GZ, 03754Z6, 03754ZZ, 0376346, 037634Z, 0376356, 037635Z, 0376366, 037636Z, 0376376, 037637Z, 03763D6, 03763DZ, 03763E6, 03763EZ, 03763F6, 03763FZ, 03763G6, 03763GZ, 03763Z6, 03763ZZ, 0376446, 037644Z, 0376456, 037645Z, 0376466, 037646Z, 0376476, 037647Z, 03764D6, 03764DZ, 03764E6, 03764EZ, 03764F6, 03764FZ, 03764G6, 03764GZ, 03764Z6, 03764ZZ, 0377346, 037734Z, 0377356, 037735Z, 0377366, 037736Z, 0377376, 037737Z, 03773D6, 03773DZ, 03773E6, 03773EZ, 03773F6, 03773FZ, 03773G6, 03773GZ, 03773Z6, 03773ZZ, 0377446, 037744Z, 0377456, 037745Z, 0377466, 037746Z, 0377476, 037747Z, 03774D6, 03774DZ, 03774E6, 03774EZ, 03774F6, 03774FZ, 03774G6, 03774GZ, 03774Z6, 03774ZZ, 0378346, 037834Z, 0378356, 037835Z, 0378366, 037836Z, 0378376, 037837Z, 03783D6, 03783DZ, 03783E6, 03783EZ, 03783F6, 03783FZ, 03783G6, 03783GZ, 03783Z6, 03783ZZ, 0378446, 037844Z, 0378456, 037845Z, 0378466, 037846Z, 0378476, 037847Z, 03784D6, 03784DZ, 03784E6, 03784EZ, 03784F6, 03784FZ, 03784G6, 03784GZ, 03784Z6, 03784ZZ, 0379346, 037934Z, 0379356, 037935Z, 0379366, 037936Z, 0379376, 037937Z, 03793D6, 03793DZ, 03793E6, 03793EZ, 03793F6, 03793FZ, 03793G6, 03793GZ, 03793Z6, 03793ZZ, 0379446, 037944Z, 0379456, 037945Z, 0379466, 037946Z, 0379476, 037947Z, 03794D6, 03794DZ, 03794E6, 03794EZ, 03794F6, 03794FZ, 03794G6, 03794GZ, 03794Z6, 03794ZZ, 037A346, 037A34Z, 037A356, 037A35Z, 037A366, 037A36Z, 037A376, 037A37Z, 037A3D6, 037A3DZ, 037A3E6, 037A3EZ, 037A3F6, 037A3FZ, 037A3G6, 037A3GZ, 037A3Z6, 037A3ZZ, 037A446, 037A44Z, 037A456, 037A45Z, 037A466, 037A46Z, 037A476, 037A47Z, 037A4D6, 037A4DZ, 037A4E6, 037A4EZ, 037A4F6, 037A4FZ, 037A4G6, 037A4GZ, 037A4Z6, 037A4ZZ, 037B346, 037B34Z, 037B356, 037B35Z, 037B366, 037B36Z, 037B376, 037B37Z, 037B3D6, 037B3DZ, 037B3E6, 037B3EZ, 037B3F6, 037B3FZ, 037B3G6, 037B3GZ, 037B3Z6, 037B3ZZ, 037B446, 037B44Z, 037B456, 037B45Z, 037B466, 037B46Z, 037B476, 037B47Z, 037B4D6, 037B4DZ, 037B4E6, 037B4EZ, 037B4F6, 037B4FZ, 037B4G6, 037B4GZ, 037B4Z6, 037B4ZZ, 037C346, 037C34Z, 037C356, 037C35Z, 037C366, 037C36Z, 037C376, 037C37Z, 037C3D6, 037C3DZ, 037C3E6, 037C3EZ, 037C3F6, 037C3FZ, 037C3G6, 037C3GZ, 037C3Z6, 037C3ZZ, 037C446, 037C44Z, 037C456, 037C45Z, 037C466, 037C46Z, 037C476, 037C47Z, 037C4D6, 037C4DZ, 037C4E6, 037C4EZ, 037C4F6, 037C4FZ, 037C4G6, 037C4GZ, 037C4Z6, 037C4ZZ, 03C33Z6, 03C33ZZ, 03C34Z6, 03C34ZZ, 03C43Z6, 03C43ZZ, 03C44Z6, 03C44ZZ, 03C53Z6, 03C53ZZ, 03C54Z6, 03C54ZZ, 03C63Z6, 03C63ZZ, 03C64Z6, 03C64ZZ, 03C73Z6, 03C73ZZ, 03C74Z6, 03C74ZZ, 03C83Z6, 03C83ZZ, 03C84Z6, 03C84ZZ, 03C93Z6, 03C93ZZ, 03C94Z6, 03C94ZZ, 03CA3Z6, 03CA3ZZ, 03CA4Z6, 03CA4ZZ, 03CB3Z6, 03CB3ZZ, 03CB4Z6, 03CB4ZZ, 03CC3Z6, 03CC3ZZ, 03CC4Z6, 03CC4ZZ, 03CY3Z6, 03CY3ZZ, 03CY4Z6, 03CY4ZZ, 03Q33ZZ, 03Q34ZZ, 03Q43ZZ, 03Q44ZZ, 03Q53ZZ, 03Q54ZZ, 03Q63ZZ, 03Q64ZZ, 03Q73ZZ, 03Q74ZZ, 03Q83ZZ, 03Q84ZZ, 03Q93ZZ, 03Q94ZZ, 03QA3ZZ, 03QA4ZZ, 03QB3ZZ, 03QB4ZZ, 03QC3ZZ, 03QC4ZZ, 03V33CZ, 03V34CZ, 03V34ZZ, 03V43CZ, 03V44CZ, 03V44ZZ, 03V53CZ, 03V54CZ, 03V54ZZ, 03V63CZ, 03V64CZ, 03V64ZZ, 03V73CZ, 03V74CZ, 03V74ZZ, 03V83CZ, 03V84CZ, 03V84ZZ, 03V93CZ, 03V94CZ, 03V94ZZ, 03VA3CZ, 03VA4CZ, 03VA4ZZ,

03VB3CZ, 03VB4CZ, 03VB4ZZ, 03VC3CZ, 03VC4CZ, 03VC4ZZ, 03WY37Z, 03WY3KZ, 03WY47Z, 03WY4KZ
**Endo venous procedures - lower**<br>06703DZ, 06703ZZ, 06704DZ, 06704ZZ, 06713DZ, 06713ZZ, 06714DZ, 06714ZZ, 06723DZ, 06723ZZ, 06724DZ, 06724ZZ, 06733DZ, 06733ZZ, 06734DZ, 06734ZZ, 06743DZ, 06743ZZ, 06744DZ, 06744ZZ, 06753DZ, 06753ZZ, 06754DZ, 06754ZZ, 06763DZ, 06763ZZ, 06764DZ, 06764ZZ, 06773DZ, 06773ZZ, 06774DZ, 06774ZZ, 06783DZ, 06783ZZ, 06784DZ, 06784ZZ, 06793DZ, 06793ZZ, 06794DZ, 06794ZZ, 067B3DZ, 067B3ZZ, 067B4DZ, 067B4ZZ, 067C3DZ, 067C3ZZ, 067C4DZ, 067C4ZZ, 067D3DZ, 067D3ZZ, 067D4DZ, 067D4ZZ, 067F3DZ, 067F3ZZ, 067F4DZ, 067F4ZZ, 067G3DZ, 067G3ZZ, 067G4DZ, 067G4ZZ, 067H3DZ, 067H3ZZ, 067H4DZ, 067H4ZZ, 067J3DZ, 067J3ZZ, 067J4DZ, 067J4ZZ, 067M3DZ, 067M3ZZ, 067M4DZ, 067M4ZZ, 067N3DZ, 067N3ZZ, 067N4DZ, 067N4ZZ, 067P3DZ, 067P3ZZ, 067P4DZ, 067P4ZZ, 067Q3DZ, 067Q3ZZ, 067Q4DZ, 067Q4ZZ, 067R3DZ, 067R3ZZ, 067R4DZ, 067R4ZZ, 067S3DZ, 067S3ZZ, 067S4DZ, 067S4ZZ, 067Y3DZ, 067Y3ZZ, 067Y4DZ, 067Y4ZZ, 06C04ZZ, 06C13ZZ, 06C14ZZ, 06C23ZZ, 06C24ZZ, 06C33ZZ, 06C34ZZ, 06C43ZZ, 06C44ZZ, 06C53ZZ, 06C54ZZ, 06C63ZZ, 06C64ZZ, 06C73ZZ, 06C74ZZ, 06C83ZZ, 06C84ZZ, 06C93ZZ, 06C94ZZ, 06CB3ZZ, 06CB4ZZ, 06CC3ZZ, 06CC4ZZ, 06CD3ZZ, 06CD4ZZ, 06CF3ZZ, 06CF4ZZ, 06CG3ZZ, 06CG4ZZ, 06CH3ZZ, 06CH4ZZ, 06CJ3ZZ, 06CJ4ZZ, 06CM3ZZ, 06CN3ZZ, 06CP3ZZ, 06CQ3ZZ, 06CR3ZZ, 06CS3ZZ, 06CY3ZZ, 06CY4ZZ, 06DM0ZZ, 06DM3ZZ, 06DM4ZZ, 06DN0ZZ, 06DN3ZZ, 06DN4ZZ, 06DP0ZZ, 06DP3ZZ, 06DP4ZZ, 06DQ0ZZ, 06DQ3ZZ, 06DQ4ZZ, 06DR0ZZ, 06DR3ZZ, 06DR4ZZ, 06DS0ZZ, 06DS3ZZ, 06DS4ZZ, 06DT0ZZ, 06DT3ZZ, 06DT4ZZ, 06DV0ZZ, 06DV3ZZ, 06DV4ZZ, 06DY0ZZ, 06DY3ZZ, 06DY4ZZ, 06LM0CZ, 06LM0DZ, 06LM0ZZ, 06LM3CZ, 06LM3DZ, 06LM3ZZ, 06LM4CZ, 06LM4DZ, 06LM4ZZ, 06LN0CZ, 06LN0DZ, 06LN0ZZ, 06LN3CZ, 06LN3DZ, 06LN3ZZ, 06LN4CZ, 06LN4DZ, 06LN4ZZ, 06LP0CZ, 06LP0DZ, 06LP0ZZ, 06LP3CZ, 06LP3DZ, 06LP3ZZ, 06LP4CZ, 06LP4DZ, 06LP4ZZ, 06LQ0CZ, 06LQ0DZ, 06LQ0ZZ, 06LQ3CZ, 06LQ3DZ, 06LQ3ZZ, 06LQ4CZ, 06LQ4DZ, 06LQ4ZZ, 06LR0CZ, 06LR0DZ, 06LR0ZZ, 06LR3CZ, 06LR3DZ, 06LR3ZZ, 06LR4CZ, 06LR4DZ, 06LR4ZZ, 06LS0CZ, 06LS0DZ, 06LS0ZZ, 06LS3CZ, 06LS3DZ, 06LS3ZZ, 06LS4CZ, 06LS4DZ, 06LS4ZZ, 06LT0CZ, 06LT0DZ, 06LT0ZZ, 06LT3CZ, 06LT3DZ, 06LT3ZZ, 06LT4CZ, 06LT4DZ, 06LT4ZZ, 06LV0CZ, 06LV0DZ, 06LV0ZZ, 06LV3CZ, 06LV3DZ, 06LV3ZZ, 06LV4CZ, 06LV4DZ, 06LV4ZZ, 06PY37Z, 06PY3CZ, 06PY3DZ, 06PY3JZ, 06PY3KZ, 06PY47Z, 06PY4CZ, 06PY4DZ, 06PY4JZ, 06PY4KZ, 06WY37Z, 06WY3CZ, 06WY3DZ, 06WY3JZ, 06WY3KZ, 06WY47Z, 06WY4CZ, 06WY4DZ, 06WY4JZ, 06WY4KZ, 3E030TZ, 3E033TZ
**Endo venous procedures - upper**<br>027V34Z, 027V3DZ, 027V3ZZ, 027V44Z, 027V4DZ, 027V4ZZ, 05753DZ, 05753ZZ, 05754DZ, 05754ZZ, 05763DZ, 05763ZZ, 05764DZ, 05764ZZ, 05773DZ, 05773ZZ, 05774DZ, 05774ZZ, 05783DZ, 05783ZZ, 05784DZ, 05784ZZ, 05793DZ, 05793ZZ, 05794DZ, 05794ZZ, 057A3DZ, 057A3ZZ, 057A4DZ, 057A4ZZ, 057B3DZ, 057B3ZZ, 057B4DZ, 057B4ZZ, 057C3DZ, 057C3ZZ, 057C4DZ, 057C4ZZ, 057D3DZ, 057D3ZZ, 057D4DZ, 057D4ZZ, 057F3DZ, 057F3ZZ, 057F4DZ, 057F4ZZ, 057Y3DZ, 057Y3ZZ, 057Y4DZ, 057Y4ZZ, 05C50ZZ, 05C53ZZ, 05C54ZZ, 05C54ZZ, 05C60ZZ, 05C63ZZ, 05C64ZZ, 05C64ZZ, 05C73ZZ, 05C74ZZ, 05C83ZZ, 05C84ZZ, 05C93ZZ, 05C94ZZ, 05CA3ZZ, 05CA4ZZ, 05CB3ZZ, 05CB4ZZ, 05CC3ZZ, 05CC4ZZ, 05CD3ZZ, 05CD4ZZ, 05CF3ZZ, 05CF4ZZ, 05WY37Z, 05WY3JZ, 05WY3KZ, 05WY47Z, 05WY4JZ, 05WY4KZ
**EVAR (Endovascular Aneurysm Repair)**<br>04H03DZ, 04Q03ZZ, 04Q04ZZ, 04R047Z, 04R04JZ, 04R04KZ, 04U037Z, 04U03JZ, 04U03KZ, 04U047Z, 04U04JZ, 04U04KZ, 04V03D6, 04V03DJ, 04V03DZ, 04V03E6, 04V03EZ, 04V03F6, 04V03FZ, 04V03Z6, 04V03ZZ, 04V04D6, 04V04DJ, 04V04DZ, 04V04E6, 04V04EZ, 04V04F6, 04V04FZ, 04V04Z6, 04V04ZZ
**Extra-anatomic bypass**<br>0315096-0315099, 031509B, 031509C, 03150A6-03150A9, 03150AB, 03150AC, 03150J6-03150J9, 03150JB, 03150JC, 03150K6-03150K9, 03150KB, 03150KC, 03150Z6-03150Z9, 03150ZB, 03150ZC, 0316096-0316099, 031609B, 031609C, 03160A6-03160A9, 03160AB, 03160AC, 03160J6-03160J9, 03160JB, 03160JC, 03160K6-03160K9, 03160KB, 03160KC, 03160Z6-03160Z9, 03160ZB, 03160ZC
**Infrainguinal PVI (peripheral vascular interventions)**<br>047K341, 047K346, 047K34Z, 047K356, 047K35Z, 047K366, 047K36Z, 047K376, 047K37Z, 047K3D1, 047K3D6, 047K3DZ, 047K3E6, 047K3EZ, 047K3F6, 047K3FZ, 047K3G6, 047K3GZ, 047K3Z1, 047K3Z6, 047K3ZZ, 047K441, 047K446, 047K44Z, 047K456, 047K45Z, 047K466,

047K46Z, 047K476, 047K47Z, 047K4D1, 047K4D6, 047K4DZ, 047K4E6, 047K4EZ, 047K4F6, 047K4FZ, 047K4G6, 047K4GZ, 047K4Z1, 047K4Z6, 047K4ZZ, 047L341, 047L346, 047L34Z, 047L356, 047L35Z, 047L366, 047L36Z, 047L376, 047L37Z, 047L3D1, 047L3D6, 047L3DZ, 047L3E6, 047L3EZ, 047L3F6, 047L3FZ, 047L3G6, 047L3GZ, 047L3Z1, 047L3Z6, 047L3ZZ, 047L441, 047L446, 047L44Z, 047L456, 047L45Z, 047L466, 047L46Z, 047L476, 047L47Z, 047L4D1, 047L4D6, 047L4DZ, 047L4E6, 047L4EZ, 047L4F6, 047L4FZ, 047L4G6, 047L4GZ, 047L4Z1, 047L4Z6, 047L4ZZ, 047M341, 047M346, 047M34Z, 047M356, 047M35Z, 047M366, 047M36Z, 047M376, 047M37Z, 047M3D1, 047M3D6, 047M3DZ, 047M3E6, 047M3EZ, 047M3F6, 047M3FZ, 047M3G6, 047M3GZ, 047M3Z1, 047M3Z6, 047M3ZZ, 047M441, 047M446, 047M44Z, 047M456, 047M45Z, 047M466, 047M46Z, 047M476, 047M47Z, 047M4D1, 047M4D6, 047M4DZ, 047M4E6, 047M4EZ, 047M4F6, 047M4FZ, 047M4G6, 047M4GZ, 047M4Z1, 047M4Z6, 047M4ZZ, 047N341, 047N346, 047N34Z, 047N356, 047N35Z, 047N366, 047N36Z, 047N376, 047N37Z, 047N3D1, 047N3D6, 047N3DZ, 047N3E6, 047N3EZ, 047N3F6, 047N3FZ, 047N3G6, 047N3GZ, 047N3Z1, 047N3Z6, 047N3ZZ, 047N441, 047N446, 047N44Z, 047N456, 047N45Z, 047N466, 047N46Z, 047N476, 047N47Z, 047N4D1, 047N4D6, 047N4DZ, 047N4E6, 047N4EZ, 047N4F6, 047N4FZ, 047N4G6, 047N4GZ, 047N4Z1, 047N4Z6, 047N4ZZ, 047P341, 047P346, 047P34Z, 047P356, 047P35Z, 047P366, 047P36Z, 047P376, 047P37Z, 047P3D1, 047P3D6, 047P3DZ, 047P3E6, 047P3EZ, 047P3F6, 047P3FZ, 047P3G6, 047P3GZ, 047P3Z1, 047P3Z6, 047P3ZZ, 047P441, 047P446, 047P44Z, 047P456, 047P45Z, 047P466, 047P46Z, 047P476, 047P47Z, 047P4D1, 047P4D6, 047P4DZ, 047P4E6, 047P4EZ, 047P4F6, 047P4FZ, 047P4G6, 047P4GZ, 047P4Z1, 047P4Z6, 047P4ZZ, 047Q341, 047Q346, 047Q34Z, 047Q356, 047Q35Z, 047Q366, 047Q36Z, 047Q376, 047Q37Z, 047Q3D1, 047Q3D6, 047Q3DZ, 047Q3E6, 047Q3EZ, 047Q3F6, 047Q3FZ, 047Q3G6, 047Q3GZ, 047Q3Z1, 047Q3Z6, 047Q3ZZ, 047Q441, 047Q446, 047Q44Z, 047Q456, 047Q45Z, 047Q466, 047Q46Z, 047Q476, 047Q47Z, 047Q4D1, 047Q4D6, 047Q4DZ, 047Q4E6, 047Q4EZ, 047Q4F6, 047Q4FZ, 047Q4G6, 047Q4GZ, 047Q4Z1, 047Q4Z6, 047Q4ZZ, 047R341, 047R346, 047R34Z, 047R356, 047R35Z, 047R366, 047R36Z, 047R376, 047R37Z, 047R3D1, 047R3D6, 047R3DZ, 047R3E6, 047R3EZ, 047R3F6, 047R3FZ, 047R3G6, 047R3GZ, 047R3Z1, 047R3Z6, 047R3ZZ, 047R441, 047R446, 047R44Z, 047R456, 047R45Z, 047R466, 047R46Z, 047R476, 047R47Z, 047R4D1, 047R4D6, 047R4DZ, 047R4E6, 047R4EZ, 047R4F6, 047R4FZ, 047R4G6, 047R4GZ, 047R4Z1, 047R4Z6, 047R4ZZ, 047S341, 047S346, 047S34Z, 047S356, 047S35Z, 047S366, 047S36Z, 047S376, 047S37Z, 047S3D1, 047S3D6, 047S3DZ, 047S3E6, 047S3EZ, 047S3F6, 047S3FZ, 047S3G6, 047S3GZ, 047S3Z1, 047S3Z6, 047S3ZZ, 047S441, 047S446, 047S44Z, 047S456, 047S45Z, 047S466, 047S46Z, 047S476, 047S47Z, 047S4D1, 047S4D6, 047S4DZ, 047S4E6, 047S4EZ, 047S4F6, 047S4FZ, 047S4G6, 047S4GZ, 047S4Z1, 047S4Z6, 047S4ZZ, 047T341, 047T346, 047T34Z, 047T356, 047T35Z, 047T366, 047T36Z, 047T376, 047T37Z, 047T3D1, 047T3D6, 047T3DZ, 047T3E6, 047T3EZ, 047T3F6, 047T3FZ, 047T3G6, 047T3GZ, 047T3Z1, 047T3Z6, 047T3ZZ, 047T441, 047T446, 047T44Z, 047T456, 047T45Z, 047T466, 047T46Z, 047T476, 047T47Z, 047T4D1, 047T4D6, 047T4DZ, 047T4E6, 047T4EZ, 047T4F6, 047T4FZ, 047T4G6, 047T4GZ, 047T4Z1, 047T4Z6, 047T4ZZ, 047U341, 047U346, 047U34Z, 047U356, 047U35Z, 047U366, 047U36Z, 047U376, 047U37Z, 047U3D1, 047U3D6, 047U3DZ, 047U3E6, 047U3EZ, 047U3F6, 047U3FZ, 047U3G6, 047U3GZ, 047U3Z1, 047U3Z6, 047U3ZZ, 047U441, 047U446, 047U44Z, 047U456, 047U45Z, 047U466, 047U46Z, 047U476, 047U47Z, 047U4D1, 047U4D6, 047U4DZ, 047U4E6, 047U4EZ, 047U4F6, 047U4FZ, 047U4G6, 047U4GZ, 047U4Z1, 047U4Z6, 047U4ZZ, 04CK3Z6, 04CK3ZZ, 04CK4Z6, 04CK4ZZ, 04CL3Z6, 04CL3ZZ, 04CL4Z6, 04CL4ZZ, 04CM3Z6, 04CM3ZZ, 04CM4Z6, 04CM4ZZ, 04CN3Z6, 04CN3ZZ, 04CN4Z6, 04CN4ZZ, 04CP3Z6, 04CP3ZZ, 04CP4Z6, 04CP4ZZ, 04CQ3Z6, 04CQ3ZZ, 04CQ4Z6, 04CQ4ZZ, 04CR3Z6, 04CR3ZZ, 04CR4Z6, 04CR4ZZ, 04CS3Z6, 04CS3ZZ, 04CS4Z6, 04CS4ZZ, 04CT3Z6, 04CT3ZZ, 04CT4Z6, 04CT4ZZ, 04CU3Z6, 04CU3ZZ, 04CU4Z6, 04CU4ZZ, 04PY37Z, 04PY3JZ, 04PY3KZ, 04PY47Z, 04PY4JZ, 04PY4KZ, 04QK3ZZ, 04QK4ZZ, 04QL3ZZ, 04QL4ZZ, 04QM3ZZ, 04QM4ZZ, 04QN3ZZ, 04QN4ZZ, 04QP3ZZ, 04QP4ZZ, 04QQ3ZZ, 04QQ4ZZ, 04QR3ZZ, 04QR4ZZ, 04QS3ZZ, 04QS4ZZ, 04QT3ZZ, 04QT4ZZ, 04QU3ZZ, 04QU4ZZ, 04RK47Z, 04RK4JZ, 04RK4KZ, 04RL47Z, 04RL4JZ, 04RL4KZ, 04RM47Z, 04RM4JZ, 04RM4KZ, 04RN47Z, 04RN4JZ, 04RN4KZ, 04RP47Z, 04RP4JZ, 04RP4KZ, 04RQ47Z, 04RQ4JZ, 04RQ4KZ, 04RR47Z, 04RR4JZ, 04RR4KZ, 04RS47Z, 04RS4JZ, 04RS4KZ, 04RT47Z, 04RT4JZ, 04RT4KZ, 04RU47Z, 04RU4JZ, 04RU4KZ, 04UK37Z, 04UK3JZ, 04UK3KZ, 04UK47Z, 04UK4JZ, 04UK4KZ, 04UL37Z, 04UL3JZ, 04UL3KZ, 04UL47Z, 04UL4JZ, 04UL4KZ, 04UM37Z, 04UM3JZ, 04UM3KZ, 04UM47Z, 04UM4JZ, 04UM4KZ, 04UN37Z, 04UN3JZ, 04UN3KZ,

04UN47Z, 04UN4JZ, 04UN4KZ, 04UP37Z, 04UP3JZ, 04UP3KZ, 04UP47Z, 04UP4JZ, 04UP4KZ, 04UQ37Z, 04UQ3JZ, 04UQ3KZ, 04UQ47Z, 04UQ4JZ, 04UQ4KZ, 04UR37Z, 04UR3JZ, 04UR3KZ, 04UR47Z, 04UR4JZ, 04UR4KZ, 04US37Z, 04US3JZ, 04US3KZ, 04US47Z, 04US4JZ, 04US4KZ, 04UT37Z, 04UT3JZ, 04UT3KZ, 04UT47Z, 04UT4JZ, 04UT4KZ, 04UU37Z, 04UU3JZ, 04UU3KZ, 04UU47Z, 04UU4JZ, 04UU4KZ, 04VK3DZ, 04VK3ZZ, 04VK4DZ, 04VL3DZ, 04VL3ZZ, 04VL4DZ, 04VM3DZ, 04VM3ZZ, 04VM4DZ, 04VN3DZ, 04VN3ZZ, 04VN4DZ, 04VP3DZ, 04VP3ZZ, 04VP4DZ, 04VQ3DZ, 04VQ3ZZ, 04VQ4DZ, 04VR3DZ, 04VR3ZZ, 04VR4DZ, 04VS3DZ, 04VS3ZZ, 04VS4DZ, 04VT3DZ, 04VT3ZZ, 04VT4DZ, 04VU3DZ, 04VU3ZZ, 04VU4DZ, 04WY37Z, 04WY3DZ, 04WY3JZ, 04WY3KZ, 04WY47Z, 04WY4DZ, 04WY4JZ, 04WY4KZ, X27H385, X27H395, X27H3B5, X27H3C5, X27J385, X27J395, X27J3B5, X27J3C5, X27K385, X27K395, X27K3B5, X27K3C5, X27L385, X27L395, X27L3B5, X27L3C5, X27M385, X27M395, X27M3B5, X27M3C5, X27N385, X27N395, X27N3B5, X27N3C5, X27P385, X27P395, X27P3B5, X27P3C5, X27Q385, X27Q395, X27Q3B5, X27Q3C5, X27R385, X27R395, X27R3B5, X27R3C5, X27S385, X27S395, X27S3B5, X27S3C5, X27T385, X27T395, X27T3B5, X27T3C5, X27U385, X27U395, X27U3B5, X27U3C5
**IVC Filter**<br>06H03DZ, 06H04DZ
**Lower extremity amputation**<br>0Y620ZZ, 0Y630ZZ, 0Y640ZZ, 0Y670ZZ, 0Y680ZZ, 0Y6C0Z1-0Y6C0Z3, 0Y6D0Z1-0Y6D0Z3, 0Y6F0ZZ, 0Y6G0ZZ, 0Y6H0Z1-0Y6H0Z3, 0Y6J0Z1-0Y6J0Z3, 0Y6M0Z0, 0Y6N0Z0
**Open AAA (Abdominal Aortic Aneurysm) repair**<br>04Q00ZZ, 04R007Z, 04R00JZ, 04R00KZ, 04U007Z, 04U00JZ, 04U00KZ, 04V00D6, 04V00DJ, 04V00DZ, 04V00E6, 04V00EZ, 04V00F6, 04V00FZ, 04V00Z6, 04V00ZZ
**Open abdominal artery revascularization**<br>0410090-0410095, 04100A0-04100A5, 04100J0-04100J5, 04100K0-04100K5, 04100Z0-04100Z5, 0413093-0413095, 04130A3-04130A5, 04130J3-04130J5, 04130K3-04130K5, 04130Z3-04130Z5, 0413493-0413495, 04134A3-04134A5, 04134J3-04134J5, 04134K3-04134K5, 04134Z3-04134Z5, 0414093-0414095, 04140A3-04140A5, 04140J3-04140J5, 04140K3-04140K5, 04140Z3-04140Z5, 0414493-0414495, 04144A3-04144A5, 04144J3-04144J5, 04144K3-04144K5, 04144Z3-04144Z5, 041C090-041C095, 041C0A0-041C0A5, 041C0J0-041C0J5, 041C0K0-041C0K5, 041C0Z0-041C0Z5, 041D090-041D095, 041D0A0-041D0A5, 041D0J0-041D0J5, 041D0K0-041D0K5, 041D0Z0-041D0Z5, 0470041, 0470046, 047004Z, 0470056, 047005Z, 0470066, 047006Z, 0470076, 047007Z, 04700D1, 04700D6, 04700DZ, 04700E6, 04700EZ, 04700F6, 04700FZ, 04700G6, 04700GZ, 04700Z1, 04700Z6, 04700ZZ, 0471041, 0471046, 047104Z, 0471056, 047105Z, 0471066, 047106Z, 0471076, 047107Z, 04710D1, 04710D6, 04710DZ, 04710E6, 04710EZ, 04710F6, 04710FZ, 04710G6, 04710GZ, 04710Z1, 04710Z6, 04710ZZ, 0472041, 0472046, 047204Z, 0472056, 047205Z, 0472066, 047206Z, 0472076, 047207Z, 04720D1, 04720D6, 04720DZ, 04720E6, 04720EZ, 04720F6, 04720FZ, 04720G6, 04720GZ, 04720Z1, 04720Z6, 04720ZZ, 0473041, 0473046, 047304Z, 0473056, 047305Z, 0473066, 047306Z, 0473076, 047307Z, 04730D1, 04730D6, 04730DZ, 04730E6, 04730EZ, 04730F6, 04730FZ, 04730G6, 04730GZ, 04730Z1, 04730Z6, 04730ZZ, 0474041, 0474046, 047404Z, 0474056, 047405Z, 0474066, 047406Z, 0474076, 047407Z, 04740D1, 04740D6, 04740DZ, 04740E6, 04740EZ, 04740F6, 04740FZ, 04740G6, 04740GZ, 04740Z1, 04740Z6, 04740ZZ, 0475041, 0475046, 047504Z, 0475056, 047505Z, 0475066, 047506Z, 0475076, 047507Z, 04750D1, 04750D6, 04750DZ, 04750E6, 04750EZ, 04750F6, 04750FZ, 04750G6, 04750GZ, 04750Z1, 04750Z6, 04750ZZ, 0476041, 0476046, 047604Z, 0476056, 047605Z, 0476066, 047606Z, 0476076, 047607Z, 04760D1, 04760D6, 04760DZ, 04760E6, 04760EZ, 04760F6, 04760FZ, 04760G6, 04760GZ, 04760Z1, 04760Z6, 04760ZZ, 0477041, 0477046, 047704Z, 0477056, 047705Z, 0477066, 047706Z, 0477076, 047707Z, 04770D1, 04770D6, 04770DZ, 04770E6, 04770EZ, 04770F6, 04770FZ, 04770G6, 04770GZ, 04770Z1, 04770Z6, 04770ZZ, 0478041, 0478046, 047804Z, 0478056, 047805Z, 0478066, 047806Z, 0478076, 047807Z, 04780D1, 04780D6, 04780DZ, 04780E6, 04780EZ, 04780F6, 04780FZ, 04780G6, 04780GZ, 04780Z1, 04780Z6, 04780ZZ, 0479041, 0479046, 047904Z, 0479056, 047905Z, 0479066, 047906Z, 0479076, 047907Z, 04790D1, 04790D6, 04790DZ, 04790E6, 04790EZ, 04790F6, 04790FZ, 04790G6, 04790GZ, 04790Z1, 04790Z6, 04790ZZ, 047A041, 047A046, 047A04Z, 047A056, 047A05Z, 047A066, 047A06Z, 047A076, 047A07Z, 047A0D1, 047A0D6, 047A0DZ, 047A0E6, 047A0EZ, 047A0F6, 047A0FZ, 047A0G6, 047A0GZ, 047A0Z1, 047A0Z6, 047A0ZZ, 047B041, 047B046, 047B04Z, 047B056, 047B05Z, 047B066, 047B06Z, 047B076, 047B07Z, 047B0D1, 047B0D6, 047B0DZ, 047B0E6, 047B0EZ, 047B0F6, 047B0FZ, 047B0G6, 047B0GZ, 047B0Z1, 047B0Z6, 047B0ZZ, 047C041, 047C046, 047C04Z,

047C056, 047C05Z, 047C066, 047C06Z, 047C076, 047C07Z, 047C0D1, 047C0D6, 047C0DZ, 047C0E6, 047C0EZ, 047C0F6, 047C0FZ, 047C0G6, 047C0GZ, 047C0Z1, 047C0Z6, 047C0ZZ, 047D041, 047D046, 047D04Z, 047D056, 047D05Z, 047D066, 047D06Z, 047D076, 047D07Z, 047D0D1, 047D0D6, 047D0DZ, 047D0E6, 047D0EZ, 047D0F6, 047D0FZ, 047D0G6, 047D0GZ, 047D0Z1, 047D0Z6, 047D0ZZ, 047E041, 047E046, 047E04Z, 047E056, 047E05Z, 047E066, 047E06Z, 047E076, 047E07Z, 047E0D1, 047E0D6, 047E0DZ, 047E0E6, 047E0EZ, 047E0F6, 047E0FZ, 047E0G6, 047E0GZ, 047E0Z1, 047E0Z6, 047E0ZZ, 047F041, 047F046, 047F04Z, 047F056, 047F05Z, 047F066, 047F06Z, 047F076, 047F07Z, 047F0D1, 047F0D6, 047F0DZ, 047F0E6, 047F0EZ, 047F0F6, 047F0FZ, 047F0G6, 047F0GZ, 047F0Z1, 047F0Z6, 047F0ZZ, 047H041, 047H046, 047H04Z, 047H056, 047H05Z, 047H066, 047H06Z, 047H076, 047H07Z, 047H0D1, 047H0D6, 047H0DZ, 047H0E6, 047H0EZ, 047H0F6, 047H0FZ, 047H0G6, 047H0GZ, 047H0Z1, 047H0Z6, 047H0ZZ, 047J041, 047J046, 047J04Z, 047J056, 047J05Z, 047J066, 047J06Z, 047J076, 047J07Z, 047J0D1, 047J0D6, 047J0DZ, 047J0E6, 047J0EZ, 047J0F6, 047J0FZ, 047J0G6, 047J0GZ, 047J0Z1, 047J0Z6, 047J0ZZ, 04C10Z6, 04C10ZZ, 04C20Z6, 04C20ZZ, 04C30Z6, 04C30ZZ, 04C40Z6, 04C40ZZ, 04C50Z6, 04C50ZZ, 04C60Z6, 04C60ZZ, 04C70Z6, 04C70ZZ, 04C80Z6, 04C80ZZ, 04C90Z6, 04C90ZZ, 04CA0Z6, 04CA0ZZ, 04CB0Z6, 04CB0ZZ, 04R107Z, 04R10JZ, 04R10KZ, 04R207Z, 04R20JZ, 04R20KZ, 04R307Z, 04R30JZ, 04R30KZ, 04R407Z, 04R40JZ, 04R40KZ, 04R507Z, 04R50JZ, 04R50KZ, 04R607Z, 04R60JZ, 04R60KZ, 04R707Z, 04R70JZ, 04R70KZ, 04R807Z, 04R80JZ, 04R80KZ, 04R907Z, 04R90JZ, 04R90KZ, 04RA07Z, 04RA0JZ, 04RA0KZ, 04RB07Z, 04RB0JZ, 04RB0KZ, 04RC07Z, 04RC0JZ, 04RC0KZ, 04RD07Z, 04RD0JZ, 04RD0KZ, 04RE07Z, 04RE0JZ, 04RE0KZ, 04RF07Z, 04RF0JZ, 04RF0KZ, 04RH07Z, 04RH0JZ, 04RH0KZ, 04RJ07Z, 04RJ0JZ, 04RJ0KZ, 04U107Z, 04U10JZ, 04U10KZ, 04U207Z, 04U20JZ, 04U20KZ, 04U307Z, 04U30JZ, 04U30KZ, 04U407Z, 04U40JZ, 04U40KZ, 04U507Z, 04U50JZ, 04U50KZ, 04U607Z, 04U60JZ, 04U60KZ, 04U707Z, 04U70JZ, 04U70KZ, 04U807Z, 04U80JZ, 04U80KZ, 04U907Z, 04U90JZ, 04U90KZ, 04UA07Z, 04UA0JZ, 04UA0KZ, 04UB07Z, 04UB0JZ, 04UB0KZ, 04UC07Z, 04UC0JZ, 04UC0KZ, 04UD07Z, 04UD0JZ, 04UD0KZ, 04UE07Z, 04UE0JZ, 04UE0KZ, 04UF07Z, 04UF0JZ, 04UF0KZ, 04UH07Z, 04UH0JZ, 04UH0KZ, 04UJ07Z, 04UJ0JZ, 04UJ0KZ
**Open infrainguinal revascularization**<br>041K09H, 041K09J, 041K09K, 041K09L, 041K09M, 041K09N, 041K09P, 041K09Q, 041K09S, 041K0AH, 041K0AJ, 041K0AK, 041K0AL, 041K0AM, 041K0AN, 041K0AP, 041K0AQ, 041K0AS, 041K0JH, 041K0JJ, 041K0JK, 041K0JL, 041K0JM, 041K0JN, 041K0JP, 041K0JQ, 041K0JS, 041K0KH, 041K0KJ, 041K0KK, 041K0KL, 041K0KM, 041K0KN, 041K0KP, 041K0KQ, 041K0KS, 041K0ZH, 041K0ZJ, 041K0ZK, 041K0ZL, 041K0ZM, 041K0ZN, 041K0ZP, 041K0ZQ, 041K0ZS, 041L09H, 041L09J, 041L09K, 041L09L, 041L09M, 041L09N, 041L09P, 041L09Q, 041L09S, 041L0AH, 041L0AJ, 041L0AK, 041L0AL, 041L0AM, 041L0AN, 041L0AP, 041L0AQ, 041L0AS, 041L0JH, 041L0JJ, 041L0JK, 041L0JL, 041L0JM, 041L0JN, 041L0JP, 041L0JQ, 041L0JS, 041L0KH, 041L0KJ, 041L0KK, 041L0KL, 041L0KM, 041L0KN, 041L0KP, 041L0KQ, 041L0KS, 041L0ZH, 041L0ZJ, 041L0ZK, 041L0ZL, 041L0ZM, 041L0ZN, 041L0ZP, 041L0ZQ, 041L0ZS, 041M09L, 041M09M, 041M09P, 041M09Q, 041M09S, 041M0AL, 041M0AM, 041M0AP, 041M0AQ, 041M0AS, 041M0JL, 041M0JM, 041M0JP, 041M0JQ, 041M0JS, 041M0KL, 041M0KM, 041M0KP, 041M0KQ, 041M0KS, 041M0ZL, 041M0ZM, 041M0ZP, 041M0ZQ, 041M0ZS, 041N09L, 041N09M, 041N09P, 041N09Q, 041N09S, 041N0AL, 041N0AM, 041N0AP, 041N0AQ, 041N0AS, 041N0JL, 041N0JM, 041N0JP, 041N0JQ, 041N0JS, 041N0KL, 041N0KM, 041N0KP, 041N0KQ, 041N0KS, 041N0ZL, 041N0ZM, 041N0ZP, 041N0ZQ, 041N0ZS, 041T09P, 041T09Q, 041T09S, 041T0AP, 041T0AQ, 041T0AS, 041T0JP, 041T0JQ, 041T0JS, 041T0KP, 041T0KQ, 041T0KS, 041T0ZP, 041T0ZQ, 041T0ZS, 041U09P, 041U09Q, 041U09S, 041U0AP, 041U0AQ, 041U0AS, 041U0JP, 041U0JQ, 041U0JS, 041U0KP, 041U0KQ, 041U0KS, 041U0ZP, 041U0ZQ, 041U0ZS, 041V09P, 041V09Q, 041V09S, 041V0AP, 041V0AQ, 041V0AS, 041V0JP, 041V0JQ, 041V0JS, 041V0KP, 041V0KQ, 041V0KS, 041V0ZP, 041V0ZQ, 041V0ZS, 041W09P, 041W09Q, 041W09S, 041W0AP, 041W0AQ, 041W0AS, 041W0JP, 041W0JQ, 041W0JS, 041W0KP, 041W0KQ, 041W0KS, 041W0ZP, 041W0ZQ, 041W0ZS, 047K041, 047K046, 047K04Z, 047K056, 047K05Z, 047K066, 047K06Z, 047K076, 047K07Z, 047K0D1, 047K0D6, 047K0DZ, 047K0E6, 047K0EZ, 047K0F6, 047K0FZ, 047K0G6, 047K0GZ, 047K0Z1, 047K0Z6, 047K0ZZ, 047L041, 047L046, 047L04Z, 047L056, 047L05Z, 047L066, 047L06Z, 047L076, 047L07Z, 047L0D1, 047L0D6, 047L0DZ, 047L0E6, 047L0EZ, 047L0F6, 047L0FZ, 047L0G6, 047L0GZ, 047L0Z1, 047L0Z6, 047L0ZZ, 047M041, 047M046, 047M04Z, 047M056, 047M05Z,

047M066, 047M06Z, 047M076, 047M07Z, 047M0D1, 047M0D6, 047M0DZ, 047M0E6, 047M0EZ, 047M0F6, 047M0FZ, 047M0G6, 047M0GZ, 047M0Z1, 047M0Z6, 047M0ZZ, 047N041, 047N046, 047N04Z, 047N056, 047N05Z, 047N066, 047N06Z, 047N076, 047N07Z, 047N0D1, 047N0D6, 047N0DZ, 047N0E6, 047N0EZ, 047N0F6, 047N0FZ, 047N0G6, 047N0GZ, 047N0Z1, 047N0Z6, 047N0ZZ, 047P041, 047P046, 047P04Z, 047P056, 047P05Z, 047P066, 047P06Z, 047P076, 047P07Z, 047P0D1, 047P0D6, 047P0DZ, 047P0E6, 047P0EZ, 047P0F6, 047P0FZ, 047P0G6, 047P0GZ, 047P0Z1, 047P0Z6, 047P0ZZ, 047Q041, 047Q046, 047Q04Z, 047Q056, 047Q05Z, 047Q066, 047Q06Z, 047Q076, 047Q07Z, 047Q0D1, 047Q0D6, 047Q0DZ, 047Q0E6, 047Q0EZ, 047Q0F6, 047Q0FZ, 047Q0G6, 047Q0GZ, 047Q0Z1, 047Q0Z6, 047Q0ZZ, 047R041, 047R046, 047R04Z, 047R056, 047R05Z, 047R066, 047R06Z, 047R076, 047R07Z, 047R0D1, 047R0D6, 047R0DZ, 047R0E6, 047R0EZ, 047R0F6, 047R0FZ, 047R0G6, 047R0GZ, 047R0Z1, 047R0Z6, 047R0ZZ, 047S041, 047S046, 047S04Z, 047S056, 047S05Z, 047S066, 047S06Z, 047S076, 047S07Z, 047S0D1, 047S0D6, 047S0DZ, 047S0E6, 047S0EZ, 047S0F6, 047S0FZ, 047S0G6, 047S0GZ, 047S0Z1, 047S0Z6, 047S0ZZ, 047T041, 047T046, 047T04Z, 047T056, 047T05Z, 047T066, 047T06Z, 047T076, 047T07Z, 047T0D1, 047T0D6, 047T0DZ, 047T0E6, 047T0EZ, 047T0F6, 047T0FZ, 047T0G6, 047T0GZ, 047T0Z1, 047T0Z6, 047T0ZZ, 047U041, 047U046, 047U04Z, 047U056, 047U05Z, 047U066, 047U06Z, 047U076, 047U07Z, 047U0D1, 047U0D6, 047U0DZ, 047U0E6, 047U0EZ, 047U0F6, 047U0FZ, 047U0G6, 047U0GZ, 047U0Z1, 047U0Z6, 047U0ZZ, 04CK0Z6, 04CK0ZZ, 04CL0Z6, 04CL0ZZ, 04CM0Z6, 04CM0ZZ, 04CN0Z6, 04CN0ZZ, 04CP0Z6, 04CP0ZZ, 04CQ0Z6, 04CQ0ZZ, 04CR0Z6, 04CR0ZZ, 04CS0Z6, 04CS0ZZ, 04CT0Z6, 04CT0ZZ, 04CU0Z6, 04CU0ZZ, 04PY07Z, 04PY0JZ, 04PY0KZ, 04QK0ZZ, 04QL0ZZ, 04QM0ZZ, 04QN0ZZ, 04QP0ZZ, 04QQ0ZZ, 04QR0ZZ, 04QS0ZZ, 04QT0ZZ, 04QU0ZZ, 04RK07Z, 04RK0JZ, 04RK0KZ, 04RL07Z, 04RL0JZ, 04RL0KZ, 04RM07Z, 04RM0JZ, 04RM0KZ, 04RN07Z, 04RN0JZ, 04RN0KZ, 04RP07Z, 04RP0JZ, 04RP0KZ, 04RQ07Z, 04RQ0JZ, 04RQ0KZ, 04RR07Z, 04RR0JZ, 04RR0KZ, 04RS07Z, 04RS0JZ, 04RS0KZ, 04RT07Z, 04RT0JZ, 04RT0KZ, 04RU07Z, 04RU0JZ, 04RU0KZ, 04UK07Z, 04UK0JZ, 04UK0KZ, 04UL07Z, 04UL0JZ, 04UL0KZ, 04UM07Z, 04UM0JZ, 04UM0KZ, 04UN07Z, 04UN0JZ, 04UN0KZ, 04UP07Z, 04UP0JZ, 04UP0KZ, 04UQ07Z, 04UQ0JZ, 04UQ0KZ, 04UR07Z, 04UR0JZ, 04UR0KZ, 04US07Z, 04US0JZ, 04US0KZ, 04UT07Z, 04UT0JZ, 04UT0KZ, 04UU07Z, 04UU0JZ, 04UU0KZ, 04WY07Z, 04WY0DZ, 04WY0JZ, 04WY0KZ
**Open TAA (Thoracic Aortic Aneurysm) Repair**<br>02QW0ZZ, 02UW07Z, 02UW08Z, 02UW0JZ, 02UW0KZ, 02VW0CZ, 02VW0DZ, 02VW0EZ, 02VW0FZ, 02VW0ZZ
**Open upper extremity revascularization**<br>0313090-0313095, 03130A0-03130A5, 03130J0-03130J5, 03130K0-03130K5, 03130Z0-03130Z5, 0314090-0314095, 03140A0-03140A5, 03140J0-03140J5, 03140K0-03140K5, 03140Z0-03140Z5, 0315090-0315095, 03150A0-03150A5, 03150J0-03150J5, 03150K0-03150K5, 03150Z0-03150Z5, 0316090-0316095, 03160A0-03160A5, 03160J0-03160J5, 03160K0-03160K5, 03160Z0-03160Z5, 0317090, 0317093, 03170A0, 03170A3, 03170J0, 03170J3, 03170K0, 03170K3, 03170Z0, 03170Z3, 0318091, 0318094, 03180A1, 03180A4, 03180J1, 03180J4, 03180K1, 03180K4, 03180Z1, 03180Z4, 0319093, 03190A3, 03190J3, 03190K3, 03190Z3, 031A094, 031A0A4, 031A0J4, 031A0K4, 031A0Z4, 031B093, 031B0A3, 031B0J3, 031B0K3, 031B0Z3, 031C094, 031C0A4, 031C0J4, 031C0K4, 031C0Z4, 0373046, 037304Z, 0373056, 037305Z, 0373066, 037306Z, 0373076, 037307Z, 03730D6, 03730DZ, 03730E6, 03730EZ, 03730F6, 03730FZ, 03730G6, 03730GZ, 03730Z6, 03730ZZ, 0374046, 037404Z, 0374056, 037405Z, 0374066, 037406Z, 0374076, 037407Z, 03740D6, 03740DZ, 03740E6, 03740EZ, 03740F6, 03740FZ, 03740G6, 03740GZ, 03740Z6, 03740ZZ, 0375046, 037504Z, 0375056, 037505Z, 0375066, 037506Z, 0375076, 037507Z, 03750D6, 03750DZ, 03750E6, 03750EZ, 03750F6, 03750FZ, 03750G6, 03750GZ, 03750Z6, 03750ZZ, 0376046, 037604Z, 0376056, 037605Z, 0376066, 037606Z, 0376076, 037607Z, 03760D6, 03760DZ, 03760E6, 03760EZ, 03760F6, 03760FZ, 03760G6, 03760GZ, 03760Z6, 03760ZZ, 0377046, 037704Z, 0377056, 037705Z, 0377066, 037706Z, 0377076, 037707Z, 03770D6, 03770DZ, 03770E6, 03770EZ, 03770F6, 03770FZ, 03770G6, 03770GZ, 03770Z6, 03770ZZ, 0378046, 037804Z, 0378056, 037805Z, 0378066, 037806Z, 0378076, 037807Z, 03780D6, 03780DZ, 03780E6, 03780EZ, 03780F6, 03780FZ, 03780G6, 03780GZ, 03780Z6, 03780ZZ, 0379046, 037904Z, 0379056, 037905Z, 0379066, 037906Z, 0379076, 037907Z, 03790D6, 03790DZ, 03790E6, 03790EZ, 03790F6, 03790FZ, 03790G6, 03790GZ, 03790Z6, 03790ZZ, 037A046, 037A04Z, 037A056, 037A05Z, 037A066, 037A06Z, 037A076, 037A07Z, 037A0D6, 037A0DZ, 037A0E6, 037A0EZ, 037A0F6, 037A0FZ, 037A0G6, 037A0GZ, 037A0Z6, 037A0ZZ, 037B046, 037B04Z, 037B056,

037B05Z, 037B066, 037B06Z, 037B076, 037B07Z, 037B0D6, 037B0DZ, 037B0E6, 037B0EZ, 037B0F6, 037B0FZ, 037B0G6, 037B0GZ, 037B0Z6, 037B0ZZ, 037C046, 037C04Z, 037C056, 037C05Z, 037C066, 037C06Z, 037C076, 037C07Z, 037C0D6, 037C0DZ, 037C0E6, 037C0EZ, 037C0F6, 037C0FZ, 037C0G6, 037C0GZ, 037C0Z6, 037C0ZZ, 03C30Z6, 03C30ZZ, 03C40Z6, 03C40ZZ, 03C50Z6, 03C50ZZ, 03C60Z6, 03C60ZZ, 03C70Z6, 03C70ZZ, 03C80Z6, 03C80ZZ, 03C90Z6, 03C90ZZ, 03CA0Z6, 03CA0ZZ, 03CB0Z6, 03CB0ZZ, 03CC0Z6, 03CC0ZZ, 03CY0Z6, 03CY0ZZ, 03Q30ZZ, 03Q40ZZ, 03Q50ZZ, 03Q60ZZ, 03Q70ZZ, 03Q80ZZ, 03Q90ZZ, 03QA0ZZ, 03QB0ZZ, 03QC0ZZ, 03V30CZ, 03V30ZZ, 03V40CZ, 03V40ZZ, 03V50CZ, 03V50ZZ, 03V60CZ, 03V60ZZ, 03V70CZ, 03V70ZZ, 03V80CZ, 03V80ZZ, 03V90CZ, 03V90ZZ, 03VA0CZ, 03VA0ZZ, 03VB0CZ, 03VB0ZZ, 03VC0CZ, 03VC0ZZ, 03WY07Z, 03WY0KZ
**Open aortic/iliac revascularization**<br>0410096-0410099, 041009B, 041009C, 041009D, 041009F, 041009G, 041009H, 041009J, 041009K, 041009Q, 041009R, 04100A6-04100A9, 04100AB, 04100AC, 04100AD, 04100AF, 04100AG, 04100AH, 04100AJ, 04100AK, 04100AQ, 04100AR, 04100J6-04100J9, 04100JB, 04100JC, 04100JD, 04100JF, 04100JG, 04100JH, 04100JJ, 04100JK, 04100JQ, 04100JR, 04100K6-04100K9, 04100KB, 04100KC, 04100KD, 04100KF, 04100KG, 04100KH, 04100KJ, 04100KK, 04100Z6-04100Z9, 04100ZB, 04100ZC, 04100ZD, 04100ZF, 04100ZG, 04100ZH, 04100ZJ, 04100ZK, 041C096-041C099, 041C09B, 041C09C, 041C09D, 041C09F, 041C09G, 041C09H, 041C09J, 041C09K, 041C0A6-041C0A9, 041C0AB, 041C0AC, 041C0AD, 041C0AF, 041C0AG, 041C0AH, 041C0AJ, 041C0AK, 041C0J6-041C0J9, 041C0JB, 041C0JC, 041C0JD, 041C0JF, 041C0JG, 041C0JH, 041C0JJ, 041C0JK, 041C0K6-041C0K9, 041C0KB, 041C0KC, 041C0KD, 041C0KF, 041C0KG, 041C0KH, 041C0KJ, 041C0KK, 041C0Z6-041C0Z9, 041C0ZB, 041C0ZC, 041C0ZD, 041C0ZF, 041C0ZG, 041C0ZH, 041C0ZJ, 041C0ZK, 041D096-041D099, 041D09B, 041D09C, 041D09D, 041D09F, 041D09G, 041D09H, 041D09J, 041D09K, 041D0A6-041D0A9, 041D0AB, 041D0AC, 041D0AD, 041D0AF, 041D0AG, 041D0AH, 041D0AJ, 041D0AK, 041D0J6-041D0J9, 041D0JB, 041D0JC, 041D0JD, 041D0JF, 041D0JG, 041D0JH, 041D0JJ, 041D0JK, 041D0K6-041D0K9, 041D0KB, 041D0KC, 041D0KD, 041D0KF, 041D0KG, 041D0KH, 041D0KJ, 041D0KK, 041D0Z6-041D0Z9, 041D0ZB, 041D0ZC, 041D0ZD, 041D0ZF, 041D0ZG, 041D0ZH, 041D0ZJ, 041D0ZK, 041E099, 041E09B, 041E09C, 041E09D, 041E09F, 041E09G, 041E09H, 041E09J, 041E09K, 041E0A9, 041E0AB, 041E0AC, 041E0AD, 041E0AF, 041E0AG, 041E0AH, 041E0AJ, 041E0AK, 041E0J9, 041E0JB, 041E0JC, 041E0JD, 041E0JF, 041E0JG, 041E0JH, 041E0JJ, 041E0JK, 041E0K9, 041E0KB, 041E0KC, 041E0KD, 041E0KF, 041E0KG, 041E0KH, 041E0KJ, 041E0KK, 041E0Z9, 041E0ZB, 041E0ZC, 041E0ZD, 041E0ZF, 041E0ZG, 041E0ZH, 041E0ZJ, 041E0ZK, 041E499, 041E49B, 041E49C, 041E49D, 041E49F, 041E49G, 041E49H, 041E49J, 041E49K, 041E4A9, 041E4AB, 041E4AC, 041E4AD, 041E4AF, 041E4AG, 041E4AH, 041E4AJ, 041E4AK, 041E4J9, 041E4JB, 041E4JC, 041E4JD, 041E4JF, 041E4JG, 041E4JH, 041E4JJ, 041E4JK, 041E4K9, 041E4KB, 041E4KC, 041E4KD, 041E4KF, 041E4KG, 041E4KH, 041E4KJ, 041E4KK, 041E4Z9, 041E4ZB, 041E4ZC, 041E4ZD, 041E4ZF, 041E4ZG, 041E4ZH, 041E4ZJ, 041E4ZK, 041F099, 041F09B, 041F09C, 041F09D, 041F09F, 041F09G, 041F09H, 041F09J, 041F09K, 041F0A9, 041F0AB, 041F0AC, 041F0AD, 041F0AF, 041F0AG, 041F0AH, 041F0AJ, 041F0AK, 041F0J9, 041F0JB, 041F0JC, 041F0JD, 041F0JF, 041F0JG, 041F0JH, 041F0JJ, 041F0JK, 041F0K9, 041F0KB, 041F0KC, 041F0KD, 041F0KF, 041F0KG, 041F0KH, 041F0KJ, 041F0KK, 041F0Z9, 041F0ZB, 041F0ZC, 041F0ZD, 041F0ZF, 041F0ZG, 041F0ZH, 041F0ZJ, 041F0ZK, 041H099, 041H09B, 041H09C, 041H09D, 041H09F, 041H09G, 041H09H, 041H09J, 041H09K, 041H0A9, 041H0AB, 041H0AC, 041H0AD, 041H0AF, 041H0AG, 041H0AH, 041H0AJ, 041H0AK, 041H0J9, 041H0JB, 041H0JC, 041H0JD, 041H0JF, 041H0JG, 041H0JH, 041H0JJ, 041H0JK, 041H0K9, 041H0KB, 041H0KC, 041H0KD, 041H0KF, 041H0KG, 041H0KH, 041H0KJ, 041H0KK, 041H0Z9, 041H0ZB, 041H0ZC, 041H0ZD, 041H0ZF, 041H0ZG, 041H0ZH, 041H0ZJ, 041H0ZK, 041J099, 041J09B, 041J09C, 041J09D, 041J09F, 041J09G, 041J09H, 041J09J, 041J09K, 041J0A9, 041J0AB, 041J0AC, 041J0AD, 041J0AF, 041J0AG, 041J0AH, 041J0AJ, 041J0AK, 041J0J9, 041J0JB, 041J0JC, 041J0JD, 041J0JF, 041J0JG, 041J0JH, 041J0JJ, 041J0JK, 041J0K9, 041J0KB, 041J0KC, 041J0KD, 041J0KF, 041J0KG, 041J0KH, 041J0KJ, 041J0KK, 041J0Z9, 041J0ZB, 041J0ZC, 041J0ZD, 041J0ZF, 041J0ZG, 041J0ZH, 041J0ZJ, 041J0ZK, 04C00Z6, 04C00ZZ, 04CC0Z6, 04CC0ZZ, 04CD0Z6, 04CD0ZZ, 04CE0Z6, 04CE0ZZ, 04CF0Z6, 04CF0ZZ, 04CH0Z6, 04CH0ZZ, 04CJ0Z6, 04CJ0ZZ

**Other carotid procedures**<br>031H09J, 031H09K, 031H0AJ, 031H0AK, 031H0JJ, 031H0JK, 031H0KJ, 031H0KK, 031H0ZJ, 031H0ZK, 031J09J, 031J09K, 031J0AJ, 031J0AK, 031J0JJ, 031J0JK, 031J0KJ, 031J0KK, 031J0ZJ, 031J0ZK, 031K09J, 031K09K, 031K0AJ, 031K0AK, 031K0JJ, 031K0JK, 031K0KJ, 031K0KK, 031K0ZJ, 031K0ZK, 031L09J, 031L09K, 031L0AJ, 031L0AK, 031L0JJ, 031L0JK, 031L0KJ, 031L0KK, 031L0ZJ, 031L0ZK, 031M09J, 031M09K, 031M0AJ, 031M0AK, 031M0JJ, 031M0JK, 031M0KJ, 031M0KK, 031M0ZJ, 031M0ZK, 031N09J, 031N09K, 031N0AJ, 031N0AK, 031N0JJ, 031N0JK, 031N0KJ, 031N0KK, 031N0ZJ, 031N0ZK, 03BH0ZZ, 03BH4ZZ, 03BJ0ZZ, 03BJ4ZZ, 03BK0ZZ, 03BK4ZZ, 03BL0ZZ, 03BL4ZZ, 03BM0ZZ, 03BM4ZZ, 03BN0ZZ, 03BN4ZZ, 03LH0DZ, 03LH3DZ, 03LH3ZZ, 03LH4DZ, 03LJ0DZ, 03LJ3DZ, 03LJ3ZZ, 03LJ4DZ, 03LK0CZ, 03LK0DZ, 03LK0ZZ, 03LK3CZ, 03LK3DZ, 03LK3ZZ, 03LK4CZ, 03LK4DZ, 03LK4ZZ, 03LL0CZ, 03LL0DZ, 03LL0ZZ, 03LL3CZ, 03LL3DZ, 03LL3ZZ, 03LL4CZ, 03LL4DZ, 03LL4ZZ, 03LM0DZ, 03LM3DZ, 03LM3ZZ, 03LM4DZ, 03LN0DZ, 03LN3DZ, 03LN3ZZ, 03LN4DZ, 03UH07Z, 03UH0JZ, 03UH0KZ, 03UH37Z, 03UH3JZ, 03UH3KZ, 03UH47Z, 03UH4JZ, 03UH4KZ, 03UJ07Z, 03UJ0JZ, 03UJ0KZ, 03UJ37Z, 03UJ3JZ, 03UJ3KZ, 03UJ47Z, 03UJ4JZ, 03UJ4KZ, 03UK07Z, 03UK0JZ, 03UK0KZ, 03UK37Z, 03UK3JZ, 03UK3KZ, 03UK47Z, 03UK4JZ, 03UK4KZ, 03UL07Z, 03UL0JZ, 03UL0KZ, 03UL37Z, 03UL3JZ, 03UL3KZ, 03UL47Z, 03UL4JZ, 03UL4KZ, 03UM07Z, 03UM0JZ, 03UM0KZ, 03UM37Z, 03UM3JZ, 03UM3KZ, 03UM47Z, 03UM4JZ, 03UM4KZ, 03UN07Z, 03UN0JZ, 03UN0KZ, 03UN37Z, 03UN3JZ, 03UN3KZ, 03UN47Z, 03UN4JZ, 03UN4KZ, 03VH0DZ, 03VJ0DZ, 03VK0CZ, 03VK0DZ, 03VL0CZ, 03VL0DZ, 03VM0DZ, 03VN0DZ
**TEVAR (Thoracic endovascular aortic repair)**<br>02QW3ZZ, 02QW4ZZ, 02UW37Z, 02UW38Z, 02UW3JZ, 02UW3KZ, 02UW47Z, 02UW48Z, 02UW4JZ, 02UW4KZ, 02VW3CZ, 02VW3DZ, 02VW3EZ, 02VW3FZ, 02VW3ZZ, 02VW4CZ, 02VW4DZ, 02VW4EZ, 02VW4FZ, 02VW4ZZ

Supplemental Digital Content 2. Details of the Sequential Exchange Matching Algorithm

Overview of the algorithm

The actual match uses 3 comparison groups, but the algorithm here is described for a single comparison group. There is some detail needed to describe the coordination of the 3 comparison groups, but that is described in the next section.

Each patient in the focal hospital is matched to K patients from a national comparison group. For example, the typical national comparison group had K=10, so each patient in the focal hospital is matched to 10 patients from typical hospitals around the US.

The match combines near exact matching for E=90 surgical groups (3 divisions plus 87 procedure groups) x 2 multimorbidity categories (any multimorbidity or no multimorbidity) = 180 categories. In addition, C = 110 clinical and social/demographic covariates were balanced, one of these being multimorbidity. The paper speaks of matching for 90 +110 = 200 covariates. The covariates were 3 surgical divisions (general, orthopedic, vascular), 32 general surgery categories, 33 orthopedic surgery categories, 22 vascular surgical categories, 55 comorbidities, 30 common multimorbidity types (or Qualifying Comorbidity Sets), the number of comorbidities (0 to 55 comorbidities), the number of multimorbidities (or QCS's from 0 to 226), multimorbidity (0 or 1), a risk score, and 21 socioeconomic or demographic variables, 3+32+33+22+55+30+4+21 = 200.

If exact matching for the E categories is feasible, then it is achieved in the first step and is maintained throughout the matching process. A random sequence of patients is considered as possible swaps for patients that are currently in the match to improve balance on the C covariates. Swapping or not is decided based on a measure of current covariate imbalance. This measure of covariate imbalance may decrease but never increases as a result of a swap. Another measure of covariate imbalance may terminate the swapping process before considering all patients in the nation as controls. A fair amount of detail not discussed in this section ensures that special cases do not terminate the algorithm.

Step 1 – C covariates are to be balanced. Obtain the C means for the focal hospital's covariates. For example, mean patient age in the hospital.

Step 2 – Obtain the C means and standard deviations for the national comparison group's covariates. For example, mean age in well-resourced hospitals in Medicare and standard deviation of age in well-resourced hospitals in Medicare.

Step 3 – E categories are matched exactly, e=1,…,E. Determine the focal frequency f_e in category e from the focal hospital. Determine the frequency of control patients in the national comparison set in each of the exact categories.

Step 4 – If the frequency of patients in the national comparison group g_e in exact match category e is at least K times f_e, then sample K such patients from category e in the national comparison group for the starting match. Otherwise, if $g_e > Kf_e$ then there is a deficit; so take all Kf_e comparison patients and add $g_e – Kf_e$ patients that are not from the exact category e but are in the same risk decile.

Step 5 – Begin swapping patients to improve covariate balance. Randomly order the patients in the national comparison group who are not as yet in the match. Take the first patient on this list. Use a measure of covariate imbalance, defined in Details below, to decide whether adding this patient to the matched sample would improve the measure of covariate balance. If the answer is no, continue on to the next patient on the list. If the answer is yes, add this patient to their exact match category as an extra control and recompute the measure of covariate balance. Examine all patients in that exact match category deleting the one patient whose removal most improves the measure of covariate balance. It may be that the patient that was just added is now deleted. The addition and deletion of one patient is a swap.

Step 6 – Continue swapping until the balance on covariates is judged acceptable. For example, the stopping rule we used was an absolute standardized difference in means all less than 0.01.

Detailed Description of the Match

I. Comparison Groups

Our evaluation involved 3 national comparison groups: (1) patients in well-resourced hospitals; (2) patients in typical hospitals; (3) patients in hospitals with similar characteristics to the focal hospital (the Analogous match). Before matching these national comparison groups overlapped; however, the matched samples drawn from them do not overlap. No patient is used twice in the evaluation of a single focal hospital. We implemented this by forming the well-resourced match first, taking their matched patients out of the second national comparison group, matching the typical patients, taking their patients out of the third national comparison group, and matching the third group.

The Analogous match was different from the first two. It involved more covariates, specifically covariates that describe the hospital, such as volume. Second, the initial sample of patients was adjusted to populate the exact

match categories at the beginning with patients who also came from similar hospitals as well as were in this exact match category. Third, the stopping rule was somewhat more lenient.

II. Measures of imbalance

There were 3 measures of covariate imbalance called swap, requirement, terminate. The swap measure was used to decide whether a swap of one patient for another looked advantageous. The swap measure could be overruled by the absolute requirement measure. Swapping continued until either the end of the dataset or the terminate measure determined if the covariate balance was adequate.

For each of the C covariates, there is an absolute difference in means between the focal hospital and the current matched sample divided by the national standard deviation of that covariate. These are the absolute standardized differences, D_c, c=1,…,C. Every time a patient is swapped, D_c changes.

The requirement for a proposed swap was that the max(D_c) never increase as the result of a swap.

The termination requirement was that the max(D_c) < 0.01. For the Analogous match, the termination requirement was max(D_c) < 0.05.

The swap measure was more complex. It used all of the D_c, but emphasized the larger ones. First the D_c were sorted into increasing order, $D_{(1)} < D_{(2)} < \ldots < D_{(C)}$. The measure was:

$$\sum_{c=1}^{C} \frac{D_{(c)}}{(C+1-c)}$$

Supplemental Digital Content 3. Assigning Grades and Cut Points

Letter grades were based on cut-points determined from medium and large hospitals with a volume of at least 250 over 3 years. The letter grades have no effect on P-values and mortality rates reported in the paper. In hospital j the mortality rate is m_j, and the matched controls have mortality rate c_j. Grade cut-points were based on what is known as the folded root, $\sqrt{m_j} - \sqrt{c_j}$, with the bottom 10% receiving a D, the next 30% receiving a C (technically, here, a C is still below the 50th percentile), the next 30% receiving a B, the next 20% receiving an A, and the top 10% receiving an A+. The folded-root is a compromise between the difference of mortality rates and the ratio of mortality rates in the Box-Cox transformations[1, 2] because the difference is $m_j - c_j$ and the ratio $m_j/c_j = \exp(\log(m_j)-\log(c_j))$, and these correspond to 1 for the difference, ½ for the folded-root, and 0 for the log, in the Box-Cox family. Differences in mortality are appropriate for high-risk populations (e.g., multimorbid patients) and ratios for low-risk patients (e.g., patients without multimorbidity), and we found that grades based on the folded root work with both populations.

Supplemental Digital Content 4. Balance Tables

Supplemental Digital Content Table 4. Balance Table for Focal Hospitals A and B by Matched Control Patients Treated at Well-Resourced and Typical Hospitals

		Matched Controls for Focal Hospital A			Matched Controls for Focal Hospital B	
Variable Labels	Focal Hospital (A)	Well-Resourced Controls	Typical Controls	Focal Hospital (B)	Well-Resourced Controls	Typical Controls
N patients	2,063	20,630	20,630	2,251	22,510	22,510
**3 Divisions inside each Hospital Surgery Department: Divisions of General Surgery, Orthopedic Surgery and Vascular Surgery**						
General Surgery (%)	53.5	53.5	53.5	31.2	31.2	31.2
Orthopedics (%)	19.8	19.8	19.8	48.3	48.3	48.3
Vascular Surgery (%)	26.7	26.7	26.7	20.5	20.5	20.5
**32 General Surgery Categories**						
Colectomy (%)	12.7	12.7	12.7	7.5	7.5	7.5
Pancreatectomy (%)	6.7	6.7	6.7	1.6	1.6	1.6
Hernia Abdomen (%)	4.9	4.9	4.9	2.8	2.8	2.8
Cholecystectomy (%)	4.7	4.7	4.7	6.0	6.0	6.0
Enterectomy (%)	3.4	3.4	3.4	1.8	1.8	1.8
Liver (%)	3.4	3.4	3.4	0.8	0.8	0.8
Esophagectomy (%)	2.3	2.3	2.3	#	#	#
Gastrectomy (%)	2.3	2.3	2.3	#	#	#
Adrenal (%)	1.6	1.6	1.6	#	#	#
Ostomy Creation/Reversal (%)	1.4	1.4	1.4	1.2	1.2	1.2
Thyroid/Parathyroid (%)	1.4	1.4	1.4	0.6	0.6	0.6
Hernia Diaphragm (%)	1.3	1.3	1.3	2.4	2.4	2.4
Gastric Bypass (%)	1.1	1.1	1.1	#	#	#
Bariatric (%)	0.9	0.9	0.9	0.6	0.6	0.6
Hernia Groin (%)	0.9	0.9	0.9	0.6	0.6	0.6
Splenectomy (%)	0.8	0.8	0.8	#	#	#
Appendectomy (%)	0.8	0.8	0.8	#	#	#
Proctectomy (%)	0.8	0.8	0.8	0.7	0.7	0.7
Mastectomy (%)	0.7	0.7	0.7	#	#	#
Esophagomyotomy (%)	0.0	0.0	0.0	#	#	#

Variable Labels	Focal Hospital (A)	Matched Controls for Focal Hospital A		Focal Hospital (B)	Matched Controls for Focal Hospital B	
		Well-Resourced Controls	Typical Controls		Well-Resourced Controls	Typical Controls
Large Bowel, Other (%)	0.0	0.0	0.0	#	#	#
Pancreas, Other (%)	0.0	0.0	0.0	#	#	#
Biliary Common Duct (%)	#	#	#	0.0	0.0	0.0
Adhesiolysis (%)	#	#	#	0.9	0.9	0.9
Biliary Other (%)	#	#	#	#	#	#
Diagnostic Lap (%)	#	#	#	#	#	#
Lymphadenectomy (%)	#	#	#	0.0	0.0	0.0
Proctoplexy (%)	#	#	#	0.0	0.0	0.0
Small Bowel, Other (%)	#	#	#	#	#	#
Stomach Anti-Reflux (%)	#	#	#	0.6	0.6	0.6
Stomach, Other (%)	#	#	#	0.0	0.0	0.0
Ulcer (%)	#	#	#	#	#	#
**33 Orthopedic Surgery Categories**						
Thoracic/lumbar/sacral fusion (%)	8.8	8.8	8.8	6.3	6.3	6.3
Spinal decompression (%)	3.5	3.5	3.5	1.6	1.6	1.6
Cervical fusion (%)	3.0	3.0	3.0	2.3	2.3	2.3
Spinal cord/spinal meninges lesion excision/destruction (%)	1.0	1.0	1.0	#	#	#
Other spinal procedures (%)	0.9	0.9	0.9	#	#	#
Femur repair (%)	0.6	0.6	0.6	8.8	8.8	8.8
Ankle procedures (%)	0.0	0.0	0.0	#	#	#
Elbow procedures (%)	0.0	0.0	0.0	#	#	#
Femur revision (%)	0.0	0.0	0.0	0.0	0.0	0.0
Forearm procedures (%)	0.0	0.0	0.0	0.6	0.6	0.6
Ostectomy, knee (%)	0.0	0.0	0.0	#	#	#
Other fracture repair (%)	0.0	0.0	0.0	#	#	#
Other knee procedures (%)	0.0	0.0	0.0	0.7	0.7	0.7
Other shoulder procedures (%)	0.0	0.0	0.0	#	#	#
Other tendon repair (%)	0.0	0.0	0.0	0.0	0.0	0.0
Partial knee replacement (%)	0.0	0.0	0.0	#	#	#
Partial shoulder replacement (%)	0.0	0.0	0.0	#	#	#
Shoulder revision (%)	0.0	0.0	0.0	#	#	#

Variable Labels	Focal Hospital (A)	Matched Controls for Focal Hospital A		Focal Hospital (B)	Matched Controls for Focal Hospital B	
		Well-Resourced Controls	Typical Controls		Well-Resourced Controls	Typical Controls
Tibia/Fibula repair (%)	0.0	0.0	0.0	1.9	1.9	1.9
Tibia/Fibula revision (%)	0.0	0.0	0.0	#	#	#
Total shoulder replacement (%)	0.0	0.0	0.0	#	#	#
Partial hip replacement (%)	#	#	#	4.4	4.4	4.4
Total hip replacement (%)	#	#	#	7.2	7.2	7.2
Total knee replacement (%)	#	#	#	6.9	6.9	6.9
Foot procedures (%)	#	#	#	0.5	0.5	0.5
Hip revision (%)	#	#	#	0.5	0.5	0.5
Humerus procedures (%)	#	#	#	0.7	0.7	0.7
Knee revision (%)	#	#	#	1.9	1.9	1.9
Ostectomy, femur (%)	#	#	#	0.5	0.5	0.5
Ostectomy, tibia/fibula (%)	#	#	#	#	#	#
Other hip procedures (%)	#	#	#	#	#	#
Spinal revision (%)	#	#	#	#	#	#
Vertebral fracture repair (%)	#	#	#	#	#	#
**22 Vascular Surgery Categories**						
Carotid endarterectomy (%)	5.0	5.0	5.0	3.9	3.9	3.9
EVAR (Endovascular Aneurysm Repair) (%)	4.7	4.7	4.7	2.8	2.8	2.8
Open infrainguinal revascularization (%)	2.6	2.6	2.6	2.0	2.0	2.0
Infrainguinal PVI (peripheral vascular interventions) (%)	2.0	2.0	2.0	1.3	1.3	1.3
TEVAR (Thoracic endovascular aortic repair) (%)	1.7	1.7	1.7	#	#	#
Open AAA (Abdominal Aortic Aneurysm) repair (%)	1.6	1.6	1.6	0.6	0.6	0.6
Carotid artery stent (%)	1.5	1.5	1.5	1.6	1.6	1.6
Lower extremity amputation (%)	1.3	1.3	1.3	2.1	2.1	2.1
Endo abdominal artery revascularization (%)	1.1	1.1	1.1	0.7	0.7	0.7
IVC Filter (%)	1.0	1.0	1.0	1.7	1.7	1.7
Endo venous procedures - lower (%)	0.5	0.5	0.5	#	#	#
Open abdominal artery revascularization (%)	0.5	0.5	0.5	#	#	#
Endo abdominal artery repair (%)	#	#	#	0.6	0.6	0.6
Open aortic/iliac revascularization (%)	#	#	#	0.5	0.5	0.5
Dialysis Access (%)	#	#	#	#	#	#

Variable Labels	Focal Hospital (A)	Matched Controls for Focal Hospital A<br>Well-Resourced Controls	Matched Controls for Focal Hospital A<br>Typical Controls	Focal Hospital (B)	Matched Controls for Focal Hospital B<br>Well-Resourced Controls	Matched Controls for Focal Hospital B<br>Typical Controls
Endo aortic/iliac revascularization (%)	#	#	#	#	#	#
Endo upper extremity revascularization (%)	#	#	#	#	#	#
Endo venous procedures - upper (%)	#	#	#	0.6	0.6	0.6
Extra-anatomic bypass (%)	#	#	#	0.0	0.0	0.0
Open TAA (Thoracic Aortic Aneurysm) Repair (%)	#	#	#	0.0	0.0	0.0
Open upper extremity revascularization (%)	#	#	#	#	#	#
Other carotid procedures (%)	#	#	#	#	#	#
**55 Comorbidities**						
Hypertension (%)	85.4	85.7	85.7	84.6	84.9	84.9
Lipid Metabolism Disorders (%)	83.3	83.2	83.6	79.5	79.9	79.9
Vascular Diseases (%)	52.7	52.2	52.2	49.6	49.1	49.1
Endocrine and Metabolic Disorders (%)	43.3	43.4	43.3	37.2	37.7	37.6
Coronary Artery Disease (%)	41.3	41.3	41.3	40.2	40.3	40.2
Cerebrovascular Diseases (%)	31.2	30.8	30.8	30.5	30.1	30.2
Other Cancers (%)	30.5	30.2	30.3	19.1	19.5	19.5
Chronic Pulmonary Diseases (%)	28.2	28.4	28.4	28.5	28.3	28.4
Cardiac Arrhythmias (%)	27.8	27.4	27.4	28.6	28.2	28.2
Diabetes with Complications (%)	26.1	26.5	26.5	28.1	28.3	28.2
Heart Failure (%)	24.7	24.3	24.3	27.1	27.0	26.7
Severe Cancers (%)	22.3	22.1	22.1	6.8	6.7	6.6
Protein-Calorie Malnutrition (%)	21.6	21.3	21.3	13.8	13.5	13.5
Thrombocytopenia and Other Hematological Disorders (%)	21.4	21.1	21.2	12.3	12.3	12.3
Metastatic Cancers (%)	21.4	21.2	21.2	7.4	7.2	7.2
Acute Renal Failure (%)	14.0	13.7	13.7	14.8	14.5	14.5
CKD Stage 1-3 and Nephritis (%)	13.2	12.9	12.9	21.0	20.6	20.6
Other Depressive Disorders (%)	12.9	13.2	13.2	17.9	17.9	17.9
Asthma (%)	12.6	12.9	12.9	12.4	12.7	12.7
Rheumatoid Arthritis and Inflammatory Connective Tissue Disease (%)	12.6	12.9	12.9	14.7	14.9	14.8
Sepsis or Septic Shock (%)	11.6	11.3	11.4	12.4	12.2	12.4
Diabetes without Complication (%)	10.8	11.1	10.9	8.0	8.2	8.1

Variable Labels	Focal Hospital (A)	Matched Controls for Focal Hospital A		Focal Hospital (B)	Matched Controls for Focal Hospital B	
		Well-Resourced Controls	Typical Controls		Well-Resourced Controls	Typical Controls
Complications of Implants or Grafts (%)	9.6	9.9	9.7	9.5	9.8	9.8
Disorders of Immunity (%)	9.4	9.4	9.4	3.9	4.0	3.9
Morbid Obesity (%)	9.0	9.2	9.2	15.8	15.5	15.5
Acute Heart or Respiratory Failure (%)	8.7	8.5	8.5	11.2	10.9	11.0
Major Depressive, Bipolar, and Paranoid Disorders (%)	8.1	8.4	8.4	12.1	12.1	12.1
CKD Stage 4-5 or Dialysis (%)	8.0	7.8	7.8	8.5	8.3	8.3
Chronic Non-Pressure Skin Ulcers (%)	7.3	7.3	7.3	11.2	11.0	11.0
Spinal Cord and Paralytic Disorders (%)	7.3	7.1	7.1	4.2	4.2	4.0
Alzheimer's Disease and Related Dementias (%)	6.2	6.0	5.9	16.5	16.2	16.1
Other Trauma (%)	5.9	6.2	5.9	20.6	20.2	20.2
Acute Myocardial Infarction (%)	5.7	5.6	5.6	6.8	6.6	6.6
Angina Pectoris (%)	5.1	5.3	5.2	4.5	4.7	4.7
Artificial Openings for Feeding or Elimination (%)	4.8	5.0	5.0	4.1	4.2	4.1
Chronic Pancreatitis (%)	4.7	4.6	4.6	1.2	1.2	1.2
Liver Diseases (%)	4.7	4.6	4.6	3.6	3.5	3.5
Major Organ Transplant (%)	4.4	4.3	4.3	1.1	1.2	1.1
Pneumonias (%)	4.3	4.4	4.4	5.7	5.6	5.6
Home Oxygen Use (%)	4.2	4.0	4.0	6.9	6.7	6.7
Inflammatory Bowel Disease (%)	4.1	4.0	4.0	1.9	2.0	1.9
Substance Use Disorder (%)	3.8	4.0	4.0	4.2	4.4	4.4
Unstable Angina and Other Acute Ischemic Heart Disease (%)	3.3	3.4	3.3	3.4	3.4	3.4
Other Neurological Disorders (%)	3.1	3.2	3.2	3.4	3.4	3.4
Pressure Ulcer of Skin (%)	3.0	3.0	3.0	4.4	4.3	4.4
Seizure Disorders and Convulsions (%)	3.0	3.1	3.0	3.8	3.8	3.8
Home Hospital Bed or Wheelchair Use (%)	2.6	2.6	2.6	4.0	3.9	3.9
Opportunistic Infections (%)	2.3	2.2	2.2	0.9	0.9	0.9
Severe Hematological Disorders (%)	1.6	1.5	1.5	0.9	0.9	0.9
Amputation and Complications (%)	1.4	1.5	1.4	2.8	2.8	2.8
Parkinson’s and Huntington’s Disease (%)	1.4	1.5	1.4	2.4	2.4	2.4
Schizophrenia and Psychosis (%)	1.0	0.9	0.9	0.9	1.0	1.0

Variable Labels	Focal Hospital (A)	Matched Controls for Focal Hospital A		Focal Hospital (B)	Matched Controls for Focal Hospital B	
		Well-Resourced Controls	Typical Controls		Well-Resourced Controls	Typical Controls
Head Trauma (%)	1.0	1.1	1.0	2.4	2.4	2.4
HIV/AIDS (%)	#	#	#	#	#	#
Respirator Dependence and Tracheostomy (%)	#	#	#	#	#	#
**10 Most Common Multimorbidity Types: General Surgery**						
Protein-Calorie Malnutrition	13.9	14.1	14.1	6.0	6.2	6.2
Thrombocytopenia and Other Hematological Disorders | Hypertension	9.7	9.5	9.6	2.7	2.8	2.7
Sepsis or Septic Shock	6.8	6.7	6.7	5.6	5.4	5.4
Heart Failure | Vascular Diseases	6.4	6.5	6.4	4.7	4.7	4.7
Cardiac Arrhythmias | Chronic Pulmonary Diseases	4.7	4.6	4.6	3.1	3.1	3.1
Heart Failure | Chronic Pulmonary Diseases	4.3	4.3	4.3	3.2	3.2	3.2
Diabetes with Complications | Heart Failure	4.1	4.0	4.1	2.8	2.8	2.8
Acute Heart or Respiratory Failure	4.0	4.0	4.0	4.0	3.9	3.9
Vascular Diseases | Chronic Pulmonary Diseases | Coronary Artery Disease	3.9	3.9	3.9	3.0	3.0	3.0
Heart Failure | Cardiac Arrhythmias | Coronary Artery Disease	3.5	3.7	3.6	3.2	3.1	3.2
**10 Most Common Multimorbidity Types: Orthopedics**						
Heart Failure | Vascular Diseases	2.3	2.2	2.3	7.0	7.0	7.0
Heart Failure | Coronary Artery Disease	2.2	2.2	2.2	7.7	7.6	7.6
Endocrine and Metabolic Disorders | Heart Failure	1.7	1.8	1.7	5.8	5.7	5.8
Heart Failure | Chronic Pulmonary Diseases	1.6	1.4	1.5	5.1	5.1	5.1
Diabetes with Complications | Heart Failure	1.6	1.5	1.6	4.5	4.5	4.5
Diabetes with Complications | Chronic Pulmonary Diseases	1.6	1.4	1.4	3.3	3.3	3.4
Heart Failure | Cardiac Arrhythmias	1.5	1.7	1.5	6.8	6.7	6.7
Cardiac Arrhythmias | Chronic Pulmonary Diseases	1.2	1.2	1.2	4.6	4.5	4.6
Diabetes with Complications | Cardiac Arrhythmias	1.2	1.3	1.2	4.2	4.2	4.2
Heart Failure | Other Trauma	0.8	1.0	0.9	6.0	6.0	6.0

Variable Labels	Focal Hospital (A)	Matched Controls for Focal Hospital A: Well-Resourced Controls	Matched Controls for Focal Hospital A: Typical Controls	Focal Hospital (B)	Matched Controls for Focal Hospital B: Well-Resourced Controls	Matched Controls for Focal Hospital B: Typical Controls
**10 Most Common Multimorbidity Types: Vascular Surgery**						
Thrombocytopenia and Other Hematological Disorders	7.5	7.3	7.3	3.4	3.4	3.4
Protein-Calorie Malnutrition	5.5	5.5	5.6	3.2	3.2	3.2
CKD Stage 4-5 or Dialysis	3.8	3.9	3.9	3.2	3.4	3.4
Acute Heart or Respiratory Failure	3.3	3.4	3.3	3.0	3.1	3.1
Sepsis or Septic Shock	3.2	3.2	3.2	3.2	3.1	3.2
Heart Failure | Cardiac Arrhythmias | Chronic Pulmonary Diseases	3.1	3.1	3.1	2.3	2.3	2.3
Heart Failure | Cardiac Arrhythmias | Chronic Non-Pressure Skin Ulcers	1.8	1.8	1.8	1.9	1.8	1.8
Endocrine and Metabolic Disorders | Heart Failure | Complications of Implants or Grafts	1.8	1.8	1.8	1.2	1.2	1.2
Heart Failure | Acute Myocardial Infarction	1.7	1.7	1.7	1.7	1.7	1.7
Endocrine and Metabolic Disorders | Heart Failure | Chronic Non-Pressure Skin Ulcers	1.4	1.4	1.4	1.3	1.4	1.4
**4 Risk Summaries using 55 comorbidities and 226 multimorbidity types**						
# Comorbidities of 55 types (mean)	8.0	8.0	8.0	7.8	7.8	7.8
# Multimorbidities of 226 types (mean)	2.9	2.8	2.8	3.3	3.3	3.3
Any Multimorbidity of 226 types (%)	58.1	58.1	58.1	56.2	56.2	56.2
Risk Score (logit scale) mean	-3.8	-3.7	-3.7	-4.1	-4.1	-4.1
**21 Socioeconomic, Demographic, and Admission Variables**						
Age at Date of Surgery (years, mean)	74.2	74.2	74.2	75.8	75.7	75.7
Age 65-69 (%)	26.0	26.4	26.4	22.7	23.2	23.2
Age 70-74 (%)	33.3	33.4	33.5	28.3	28.6	28.7
Age 75-79 (%)	20.9	21.0	21.0	20.1	20.1	20.0
Age 80-84 (%)	12.0	11.7	11.7	15.2	14.8	14.8
Age 85+ (%)	7.8	7.4	7.5	13.6	13.3	13.3
Male (%)	50.5	50.4	50.1	44.0	43.5	43.5
White non-Hispanic (%)	84.0	84.1	84.1	97.9	97.6	97.6
Black (%)	10.2	10.1	10.0	#	#	#

Variable Labels	Focal Hospital (A)	Matched Controls for Focal Hospital A		Focal Hospital (B)	Matched Controls for Focal Hospital B	
		Well-Resourced Controls	Typical Controls		Well-Resourced Controls	Typical Controls
Hispanic (%)	#	#	#	#	#	#
"Other," Asian, or Native American race (%)	#	#	#	1.5	1.5	1.5
Dual-eligible (%)	7.8	8.1	8.0	19.1	18.9	19.0
Not dual-eligible (%)	92.2	91.9	92.0	80.9	81.1	81.0
High poverty status (%)	7.1	7.0	7.0	1.9	2.1	2.2
Unknown poverty status (%)	#	#	#	#	#	#
Low poverty status (%)	#	#	#	#	#	#
Low education status (Less than 80% with high school diploma) (%)[b]	5.2	5.5	5.5	3.6	3.9	3.9
Unknown education status (%)	#	#	#	#	#	#
High education status (Greater than 20% below poverty level) (%)	#	#	#	#	#	#
Emergent type of admission (%)	29.4	29.0	29.0	32.6	32.2	32.2
ED Different Hospital (%)	12.5	12.2	12.2	14.8	14.6	14.6

[a] High poverty status.

[b] Low education status (Less than 80% with high school diploma).

Not reportable due to CMS cell size requirements.

Note: All 200 Standardized Differences in covariate means were less than 0.01 in absolute value for each match, i.e., extremely small.

Supplemental Digital Content 5. Hospital Balance Tables

Supplemental Digital Content Table 5a. Balance Table of Hospital Characteristics for Hospital A

Hospital Information	Focal	Analogous	Typical	Well-Resourced
Non-Teaching	0%	0%	44%	0%
Minor Teaching	0%	0%	25%	0%
Major Teaching	0%	8%	16%	57%
Very Major Teaching	100%	91%	15%	43%
Hospital bed count	724	762	469	666
GS Volume	1104	947	526	755
Orthopedic Volume	409	1357	1421	1783
Vascular Volume	550	522	343	458
High-tech hospital status *	100%	94%	80%	93%
Nurse-to-bed ratio highest 1/3**	100%	86%	49%	71%
Nurse-to-bed ratio middle 1/3**	0%	9%	33%	23%
Nurse-to-bed ratio lowest 1/3**	0%	6%	19%	7%
Nursing skill mix in highest 1/3***	100%	64%	48%	77%
Nursing skill mix in middle 1/3***	0%	32%	38%	23%
Nursing skill mix in lowest 1/3***	0%	4%	14%	0%

*The proportion of patients in hospitals that perform either open heart surgery or organ transplantation.
NR=not reportable due to CMS restrictions.
**Nurse-to-bed = RN/Beds
***Nursing skill mix = RN/(RN+LPN)

Supplemental Digital Content Table 5b. Balance Table of Hospital Characteristics for Hospital B

Hospital Information	Focal	Analogous	Typical	Well-Resourced
Non-Teaching	0%	8%	52%	0%
Minor Teaching	0%	7%	25%	0%
Major Teaching	0%	23%	13%	62%
Very Major Teaching	100%	63%	9%	38%
Hospital bed count	515	532	396	625
GS Volume	703	685	442	690
Orthopedic Volume	1087	1150	1348	1931
Vascular Volume	461	450	293	445
High-tech hospital status*	100%	98%	73%	93%
Nurse-to-bed ratio highest 1/3**	100%	75%	46%	70%
Nurse-to-bed ratio middle 1/3**	0%	16%	34%	24%
Nurse-to-bed ratio lowest 1/3**	0%	9%	20%	6%
Nursing skill mix in highest 1/3***	0%	58%	46%	76%
Nursing skill mix in middle 1/3***	100%	35%	37%	24%
Nursing skill mix in lowest 1/3***	0%	7%	17%	0%

*The proportion of patients in hospitals that perform either open heart surgery or organ transplantation.
NR=not reportable due to CMS restrictions.
**Nurse-to-bed = RN/Beds
***Nursing skill mix = RN/(RN+LPN)

Supplemental Digital Content 6: Discussion of Multiple Testing Issues When Using Report Cards

Table 2 in the paper provided summary results for the 20 hospitals with the largest surgical volume in Pennsylvania based on our study procedures. In particular, we show the Mantel-Haenzel P-value and odds ratio estimate (and 95% CI) in comparison with 10 well-resourced controls and 10 typical controls from throughout the US. Also, we show the corresponding grades and confidence intervals for the grades.

As is evident, some hospitals are significantly and substantially worse in their mortality rates than well-resourced controls. In particular, Hospital B is worse (it is hospital number 10 in this table). Also, some hospitals are significantly better than well-resourced hospitals, and in particular this is true of Hospital A (hospital number 13 in the table).

In a scientific article an evaluation of a scientific hypothesis is typically judged significant if the P-value is $\leq$ 0.05 after a correction for testing as many hypotheses as were tested. In grading hospitals, this is perhaps not the appropriate standard, but we included such a correction in this article to demonstrate that the variation in mortality rates far exceeds what can be produced by chance. No correction for multiple testing would be required by a hospital administrator evaluating their own hospital for quality improvement purposes. Similarly, a patient choosing between a few nearby hospitals has no need of a correction for a comparison involving all the hospitals in Pennsylvania.

The correction we report is the most conservative correction in widespread use, namely the Bonferroni-Holm correction.[3] That is, among commonly used adjustments, it is least likely to report a hospital as deviant if it is not. More precisely, in 20 tests, the chance that any hospital that is not truly deviant will be reported as deviant is $\leq$ 0.05; that is, the family-wise error rate is strongly controlled. Again, we do not recommend this adjustment when evaluating one or a few hospitals because the standard gets more and more conservative as we evaluate more and more hospitals; so, it would be much more difficult for the same hospital to be judged deviant in California than Wyoming.

To illustrate this distinction in Table 2, compare hospital 4 and Hospital 10 (Hospital B in main manuscript). Compared to well-resourced hospitals, hospital 10 has an odds ratio for mortality compared to well-resourced hospitals of 1.50 while hospital 4 has an odds ratio of 1.40, and the corresponding P-values are <0.0001 and the other is 0.0046. Viewed as individual hospitals, their mortality rate is high relative to matched patients treated at well-resourced hospitals, and this is relevant to hospital administrators and patients. However, if we adjust for testing 20 hypotheses using a conservative procedure the adjusted P-value is < 0.01 for Hospital 10 but is above 0.05 for Hospital 4. So, given the extremely poor performance of hospitals 10, 16, and 18, there are far too many poor performing hospitals.

Supplemental Digital Content 7: Results for Readmissions and Revisits For Example Hospitals A and B

Supplemental Digital Content Figure 1. Hospital A report cards on Readmission and Revisits for combined general, orthopedics and vascular surgery

All Surgical Admissions

Readmission Rates

	Focal (F)	Well-Resourced (WR)	Typical (T)	Analogous (A)
N	2063	20630	20630	20630
Rate (%)	14.98	17.18	17.61	16.84
95% CI	(13.50, 16.58)	(16.68, 17.70)	(17.10, 18.14)	(16.34, 17.36)
2/3 CI	(14.23, 15.75)	(16.93, 17.44)	(17.36, 17.87)	(16.59, 17.10)

Comparaing Hospitals

	MH odds ratio	95% CI	P-value
Focal vs. Well-Resourced	0.837	(0.733, 0.954)	0.0077
Focal vs. Typical	0.809	(0.709, 0.923)	0.0016
Focal vs. Analogous	0.858	(0.752, 0.979)	0.0234
Analogous vs. Well-Resourced	0.974	(0.923, 1.028)	0.3377
Analogous vs. Typical	0.943	(0.894, 0.994)	0.0302
Well-Resourced vs. Typical	0.968	(0.917, 1.021)	0.2260

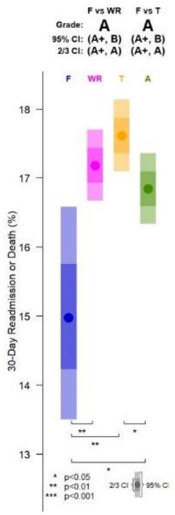

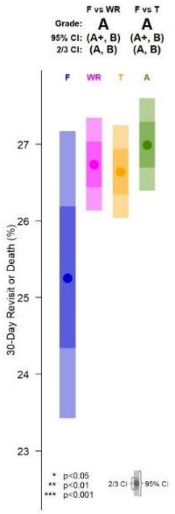

All Surgical Admissions

Revisit Rates

	Focal (F)	Well-Resourced (WR)	Typical (T)	Analogous (A)
N	2063	20630	20630	20630
Rate (%)	25.25	26.74	26.64	27.00
95% CI	(23.43, 27.17)	(26.14, 27.35)	(26.04, 27.25)	(26.39, 27.60)
2/3 CI	(24.34, 26.19)	(26.44, 27.04)	(26.34, 26.94)	(26.70, 27.29)

Comparaing Hospitals

	MH odds ratio	95% CI	P-value
Focal vs. Well-Resourced	0.920	(0.825, 1.025)	0.1305
Focal vs. Typical	0.925	(0.830, 1.031)	0.1573
Focal vs. Analogous	0.907	(0.814, 1.010)	0.0766
Analogous vs. Well-Resourced	1.014	(0.969, 1.061)	0.5406
Analogous vs. Typical	1.020	(0.975, 1.067)	0.3989
Well-Resourced vs. Typical	1.005	(0.961, 1.052)	0.8170

Supplemental Digital Content Figure 2. Hospital B report cards on Readmission and Revisits for combined general, orthopedics and vascular surgery

All Surgical Admissions

Readmission Rates

	Focal (F)	Well-Resourced (WR)	Typical (T)	Analogous (A)
N	2251	22510	22510	22510
Rate (%)	15.90	14.65	14.28	15.03
95% CI	(14.45, 17.47)	(14.20, 15.12)	(13.83, 14.75)	(14.57, 15.51)
2/3 CI	(15.17, 16.66)	(14.42, 14.88)	(14.06, 14.51)	(14.80, 15.27)

Comparaing Hospitals

	MH odds ratio	95% CI	P-value
Focal vs. Well-Resourced	1.113	(0.982, 1.261)	0.0951
Focal vs. Typical	1.151	(1.015, 1.304)	0.0286
Focal vs. Analogous	1.076	(0.950, 1.218)	0.2513
Analogous vs. Well-Resourced	1.033	(0.979, 1.091)	0.2343
Analogous vs. Typical	1.068	(1.011, 1.127)	0.0187
Well-Resourced vs. Typical	1.033	(0.978, 1.092)	0.2448

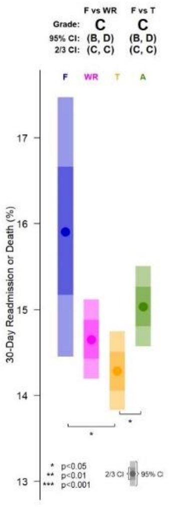

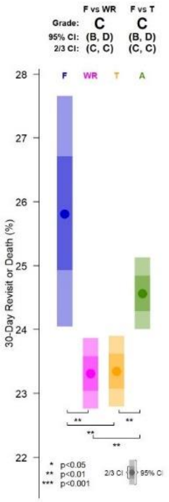

All Surgical Admissions

Revisit Rates

	Focal (F)	Well-Resourced (WR)	Typical (T)	Analogous (A)
N	2251	22510	22510	22510
Rate (%)	25.81	23.31	23.34	24.56
95% CI	(24.05, 27.66)	(22.76, 23.87)	(22.80, 23.90)	(24.00, 25.13)
2/3 CI	(24.93, 26.71)	(23.04, 23.58)	(23.07, 23.62)	(24.29, 24.84)

Comparaing Hospitals

	MH odds ratio	95% CI	P-value
Focal vs. Well-Resourced	1.159	(1.045, 1.286)	0.0055
Focal vs. Typical	1.155	(1.042, 1.281)	0.0062
Focal vs. Analogous	1.074	(0.969, 1.191)	0.1739
Analogous vs. Well-Resourced	1.077	(1.030, 1.127)	0.0012
Analogous vs. Typical	1.075	(1.028, 1.124)	0.0017
Well-Resourced vs. Typical	0.998	(0.953, 1.044)	0.9260

References

1. Box GE, Cox DR. An analysis of transformations. *J R Stat Soc Series B Stat Methodol.* 1964;26:211-243.

2. Stoto MA. The accuracy of population projections. *J Am Stat Assoc.* 1983;78:13-20.

3. Holm S. A simple sequentially rejective multiple test procedure. *Scand J Stat.* 1979;6:65-70.
